# Liver Regeneration Signature in Hepatitis B Virus (HBV)-Associated Acute Liver Failure Identified by Gene Expression Profiling

**DOI:** 10.1371/journal.pone.0049611

**Published:** 2012-11-21

**Authors:** Oriel Nissim, Marta Melis, Giacomo Diaz, David E. Kleiner, Ashley Tice, Giovanni Fantola, Fausto Zamboni, Lopa Mishra, Patrizia Farci

**Affiliations:** 1 Hepatic Pathogenesis Section, Laboratory of Infectious Diseases, National Institute of Allergy and Infectious Diseases, National Institutes of Health, Bethesda, Maryland, United States of America; 2 Department of Biomedical Sciences, University of Cagliari, Cagliari, Italy; 3 Laboratory of Pathology, National Cancer Institute, National Institutes of Health, Bethesda, Maryland, United States of America; 4 Liver Transplantation Center, Brotzu Hospital, Cagliari, Italy; 5 Department of Gastroenterology, Hepatology, and Nutrition, The University of Texas MD Anderson Cancer Center, Houston, Texas, United States of America; Drexel University College of Medicine, United States of America

## Abstract

**Introduction:**

The liver has inherent regenerative capacity via mitotic division of mature hepatocytes or, when the hepatic loss is massive or hepatocyte proliferation is impaired, through activation of hepatic stem/progenitor cells (HSPC). The dramatic clinical course of acute liver failure (ALF) has posed major limitations to investigating the molecular mechanisms of liver regeneration and the role of HSPC in this setting. We investigated the molecular mechanisms of liver regeneration in 4 patients who underwent liver transplantation for hepatitis B virus (HBV)-associated ALF.

**Methods and Findings:**

Gene expression profiling of 17 liver specimens from the 4 ALF cases and individual specimens from 10 liver donors documented a distinct gene signature for ALF. However, unsupervised multidimensional scaling and hierarchical clustering identified two clusters of ALF that segregated according to histopathological severity massive hepatic necrosis (MHN; 2 patients) and submassive hepatic necrosis (SHN; 2 patients). We found that ALF is characterized by a strong HSPC gene signature, along with ductular reaction, both of which are more prominent in MHN. Interestingly, no evidence of further lineage differentiation was seen in MHN, whereas in SHN we detected cells with hepatocyte-like morphology. Strikingly, ALF was associated with a strong tumorigenesis gene signature. MHN had the greatest upregulation of stem cell genes (EpCAM, CK19, CK7), whereas the most up-regulated genes in SHN were related to cellular growth and proliferation. The extent of liver necrosis correlated with an overriding fibrogenesis gene signature, reflecting the wound-healing process.

**Conclusion:**

Our data provide evidence for a distinct gene signature in HBV-associated ALF whose intensity is directly correlated with the histopathological severity. HSPC activation and fibrogenesis positively correlated with the extent of liver necrosis. Moreover, we detected a tumorigenesis gene signature in ALF, emphasizing the close relationship between liver regeneration and liver cancer.

## Introduction

The inherent ability of the liver to self-regenerate is attracting increasing interest because of the healing prospects that it may offer to patients with liver disorders [1]. Although significant advances have been made over the past decade in the study of liver regeneration, the molecular mechanisms of this process have yet to be elucidated [2]. Following liver damage or partial hepatectomy, the liver is restored to its proper size and function by mitotic division of mature hepatocytes [1]. However, if the hepatic loss is massive or mature hepatocyte proliferation is impaired by chronic liver disease, a reserve population of hepatic stem/progenitor cells (HSPC) localized within the canals of Hering is activated to support liver regeneration [3]. These cells have the potential to differentiate into hepatocytes and cholangiocytes, thus contributing to liver regeneration [4]. Expansion of HSPC gives rise to a characteristic histopathological alteration known as ductular reaction at the interface between the hepatic parenchyma and the portal area [5]. Studies performed by immunohistochemistry have documented that ductular reaction is a very heterogeneous process, with a remarkable cellular diversity that ranges from cells with an immature phenotype typical of stem cells to progenitor cells or “intermediate hepatobiliary cells”, to more committed cells expressing markers of hepatocyte-like or cholangiocyte-like cells [5]. HSPC express several phenotypic markers, including EpCAM, CK8, CK18, CK19, Sox9, CD44, CD24, CD133, whereas NCAM is a distinctive marker of hepatic stem cells and ICAM, AFP, and CK7 are specifically expressed by hepatic progenitor cells [6], [7].

Ductular reaction is not usually seen in normal liver, whereas in chronic liver disease it may be prominent, appearing during the late stages of disease or in conditions of chronic cholestasis [5]. This suggests that hepatocytes lose their replicative ability only after decades of chronic liver injury, which activates the HSPC response [8]. The extent of ductular reaction seems to correlate with the severity of liver disease [9], [10]. In a recent study in patients with alcoholic hepatitis, liver progenitor cell markers were found to correlate with the severity of liver disease and short-term mortality [10].

Acute liver failure (ALF) is a dramatic clinical syndrome characterized by massive necrosis of hepatic cells leading to multiorgan failure [11]. Patients who survive ALF are probably the best examples of the extraordinary ability of the liver to regenerate, a phenomenon recognized since the ancient times in the myth of Prometheus [12]. Histopathologically, ALF is characterized by extensive ductular reaction that, together with the residual portal tracts, constitutes the fundamental regenerative unit in which liver regeneration takes place [13]. However, the dramatic clinical course of ALF and the difficulties in obtaining liver samples have posed major limitations to elucidating the molecular mechanisms of liver regeneration and the role of HSPC in this setting.

The advent of gene array technologies has provided a powerful tool to study the pathogenesis of complex diseases. Nevertheless, studies of gene expression profiling of ALF in humans are very limited. Access to well-preserved explanted liver specimens from four patients with HBV-associated ALF who underwent orthotopic liver transplantation (OLT) provided us with a unique opportunity to investigate the molecular mechanisms of liver regeneration in humans by performing a systematic study of gene expression profiling. Recently, by combining microarray analysis and phage display technology, we demonstrated that HBV-associated ALF is characterized by an overwhelming intrahepatic B-cell response against the core antigen of HBV, providing evidence for a primary role of humoral immunity in the pathogenesis of this disease [14]. The aim of our study was to establish a molecular definition of liver regeneration associated with ALF, and to correlate molecular analysis with the severity of liver necrosis and the clinical features in this dramatic clinical syndrome. Our results demonstrate that HBV-related ALF is associated with expression of genes related to HSPC, hepatic stellate cell (HSC) activation and fibrogenesis, along with an overriding cell proliferation and tumorigenesis gene signature, which was not previously reported in ALF. Remarkably, our study provides evidence that the intensity of the gene signature in ALF is directly correlated with the histopathological severity.

## Materials and Methods

### Patients

Four patients with HBV-associated ALF were studied. All were previously healthy, young adult individuals who suddenly developed ALF. Two of the 4 individuals analyzed here were previously selected for a study aimed at investigating the pathogenesis of HBV-associated ALF [14]. The diagnosis of ALF was based on the occurrence of liver failure and hepatic encephalopathy within 8 weeks of the onset of the first symptoms in individuals without prior liver disease, according to previously established criteria [15]. The diagnosis of HBV- associated ALF was based on the presence of serum hepatitis B surface antigen (HBsAg) and/or IgM anti-hepatitis B core antibody (anti-HBc IgM). The 4 patients, two males and two females, had a mean age ±SD of 42.0±7 years; none of them had a history of intravenous drug addiction, alcohol abuse or tattooing. All patients suddenly developed all or some of the following symptoms, including fever, abdominal pain, vomiting, nausea, and increasing malaise, and at admission to the hospital all were jaundiced. On examination, there was no evidence of chronic liver disease, such as clubbing, spider angiomas, and splenomegaly. The clinical, biochemical, and virologic course was similar in the 4 patients (Table 1). All developed progressive encephalopathy, reaching coma grade IV and underwent OLT within 8 days from the onset of symptoms.

**Table 1:** Results of laboratory tests in four patients with HBV-associated acute liver failure.

	Massive Hepatic Necrosis (MHN)				Submassive Hepatic Necrosis (MHN)			
	Patient 241		Patient 31		Patient 219		Patient 32	
Variable	On Admission	Before OLT	On Admission	Before OLT	On Admission	Before OLT	On Admission	Before OLT
Platelet count (per mm^3^)	90,000	93,000	119,000	196,000	192,000	91,000	109,000	141,000
Creatinine (mg/dL)	0.6	1.0	0.9	0.8	2.6	3.7	1.0	1.0
Prothrombin time (INR) †	11.0	4.5	6.7	4.6	11.5	4.6	4.4	2.5
Bilirubin (mg/dL)								
Total	9.8	20.6	8.9	10.8	5.2	10.4	11.7	13.6
Conjugated		2.2	4.3	2.3	3.2	4.5	10.2	6.9
Aspartate aminotransferase (U/L)	4,000	34	9,793	924	4,565	194	6,544	1,114
Alanine aminotransferase (U/L)	5,700	252	7,573	2,537	4,710	762	11,630	2,402
Alkaline phosphatase (U/L)		167	542	401	326	291	296	290
Lactate dehydrogenase (U/L)	1,460	603	4,972	449	1,411	822	6,481	846
γ-Glutamyltransferase (U/L)		27	80	47	39	36	165	109
Alphafetoprotein (ng/mL) ‡		2.5		1.21		3.5		105.5
Serology for viral hepatitis A, C, D	Negative		Negative		Negative		Negative	
HBsAg	Positive	Negative	Positive	Positive	Positive	NA	Borderline	Negative
Concentration (µg/mL)				1.74				
Anti-HBs (mIU/mL)	0.6	67.7	0	0	15.3		5.9	8.0
Anti-HBc	Positive	Positive	Positive	Positive	Positive	NA	Positive	Positive
IgM anti-HBc	Positive	Positive	Positive	Positive	Positive	NA	Positive	Positive
HBeAg	NA	Negative	Negative	Negative	Positive	NA	Negative	Negative
Anti-HBe	NA	Positive	Borderline	Positive	Positive	NA	Positive	Positive
HBV DNA (copies/mL)	760	291		218,000	19,284		185,000	7,500
HDV RNA	Negative		Negative		Negative		Negative	
HCV RNA	Negative		Negative		Negative		Negative	

To convert the values for creatinine to µmol/L, multiply by 88.4; to convert the values for total and conjugated bilirubin to µmol/L, multiply by 17.1.

† Normal range, 0.80 to 1.20 international normalized ratio (INR).

‡ Normal range, <10.0 ng/mL. NA denotes not available.

In all patients, at admission, blood was tested for serology against hepatitis A, C and D, as well as for antibodies to cytomegalovirus, Epstein Barr virus and human immunodeficiency virus (HIV), and all were negative. To exclude coinfection with hepatitis C virus (HCV) or hepatitis D virus (HDV), serum samples were also tested for the presence of HCV RNA and HDV RNA by PCR, and all were negative (Table 1). Serial serum samples were tested for serum HBV DNA and HBV serology.

The control group was composed of 10 liver donors, 5 females and 5 males, with a mean age ±SD of 35.0±13 years. None of them had evidence of active infection with hepatitis A, B, C, and D as well as for other known viral infections that were analyzed as part of the mandatory liver donor screening. Four cases had no serological evidence of prior exposure to HBV infection, four had isolated anti-HBs and two had both anti-HBs and anti-HBc. The results of liver enzyme levels, HBV serology and liver histology of 8 out of 10 liver donors have been previously reported [14]. Histologically, the liver was normal in 6, three had very scanty fat or mild fatty changes, and one had mild portal inflammation. Liver and serum specimens along with the clinical data were received under code to protect the identity of the subjects. Written informed consent was obtained from each patient or the next of kin. The study was approved by the Office of Human Subjects Research of the National Institutes of Health, granted on the condition that all samples be made anonymous.

### Liver Specimens for Gene Expression Profiling

For each of the 4 patients (Patients 241, 31, 219, 32) who underwent OLT for HBV-associated ALF, 4 liver specimens (or 5 in Patient 219) were obtained at the time of OLT, while individual specimens were obtained from 10 liver donors. Each liver piece was divided into two pieces; one was snap-frozen and the other was fixed in formalin, as previously reported [14]. All snap-frozen liver specimens were used for gene expression profiling by means of microarray; formalin-fixed liver tissues were used for liver histology and immunohistochemistry.

### Gene Expression Profiling

Gene expression profiling was performed on all liver specimens using Affymetrix Human U133 Plus 2 arrays containing 54,675 probe sets representing approximately 29,000 human genes. Microarray analysis in Patient 31 and Patient 32 was performed previously [14]. Total RNA was extracted from frozen liver specimens using Trizol (Invitrogen), and the RNA quality and integrity were assessed using the RNA 6000 Nano assay on the Agilent 2100 Bioanalyzer, as previously reported [14]. Total liver RNA (50 ng) was subjected to two successive rounds of amplification [16]. Standard Affymetrix protocols were used for RNA labeling and hybridization, staining, washing, and scanning procedures (available at www.Affymetrix.com).

### Statistical Analysis

Microarray data obtained from the 27 liver specimens, including 17 derived from the 4 patients with ALF and 10 from normal liver donors, were analyzed using BRB-Array Tools Version 4.2 (http://linus.nci.nih.gov/BRB-ArrayTools.html) as previously reported [14]. Briefly, raw microarray data were summarized and normalized by the RMA method. Genes showing minimal variation across the set of arrays were excluded from the analysis. Genes whose expression differed by at least 1.5 fold from the median in at least 20% of the arrays were retained. A total of 11,597 transcripts passed these filtering criteria. Following normalization and filtering, unsupervised multidimensional scaling (MDS) and hierarchical clustering were performed using individual specimens from the 10 liver donors and 17 specimens from the 4 patients with ALF. The unweighted pair-group average method and Euclidean distances were used for clustering analysis. To identify genes that were differentially expressed in ALF, data were analyzed by a multivariate permutation F-test with a maximum false discovery rate of <1% with 80% confidence level [17]. The level of gene up- or down-regulation was expressed as the ratio between the geometric means of ALF and normal livers (fold change) conventionally inverted and negatively signed for ratios <1. The differential expression of single transcripts in all 27 liver specimens was also visualized by heat map. Genes were organized into functional categories according to IPA (Ingenuity® Systems, http://www.ingenuity.com) and Gene Ontology database (http://www.geneontology.org/). The complete microarray dataset has been deposited at the Gene Expression Omnibus (GEO) database, http://www.ncbi.nlm.nih.gov/geo/(accession no. GSE38941).

## Results

To investigate the mechanisms of liver regeneration, we studied 4 previously healthy young adults who underwent OLT because they suddenly developed HBV-associated ALF documented by clinical and histopathological criteria (Table 1). The clinical, biochemical, serological, and virological features were similar among the 4 patients with the exception of serum α-fetoprotein, which was elevated only in Patient 32 (105.5 ng/ml; normal range <10.0 ng/mL). At presentation, all patients were positive for IgM and IgG anti-HBc, while HBsAg was positive in Patients 241, 31 and 219, but borderline in Patient 32. At the time of OLT, HBsAg became negative in Patients 241 and 32, both of whom seroconverted to anti-HBs (Table 1). Serum HBV DNA was detectable at low levels at admission in all cases (ranging from 760 to 185,000 IU/ml), and the levels further decreased at the time of OLT in the two patients in whom serum HBV DNA was tested (Table 1).

**Figure 1:**
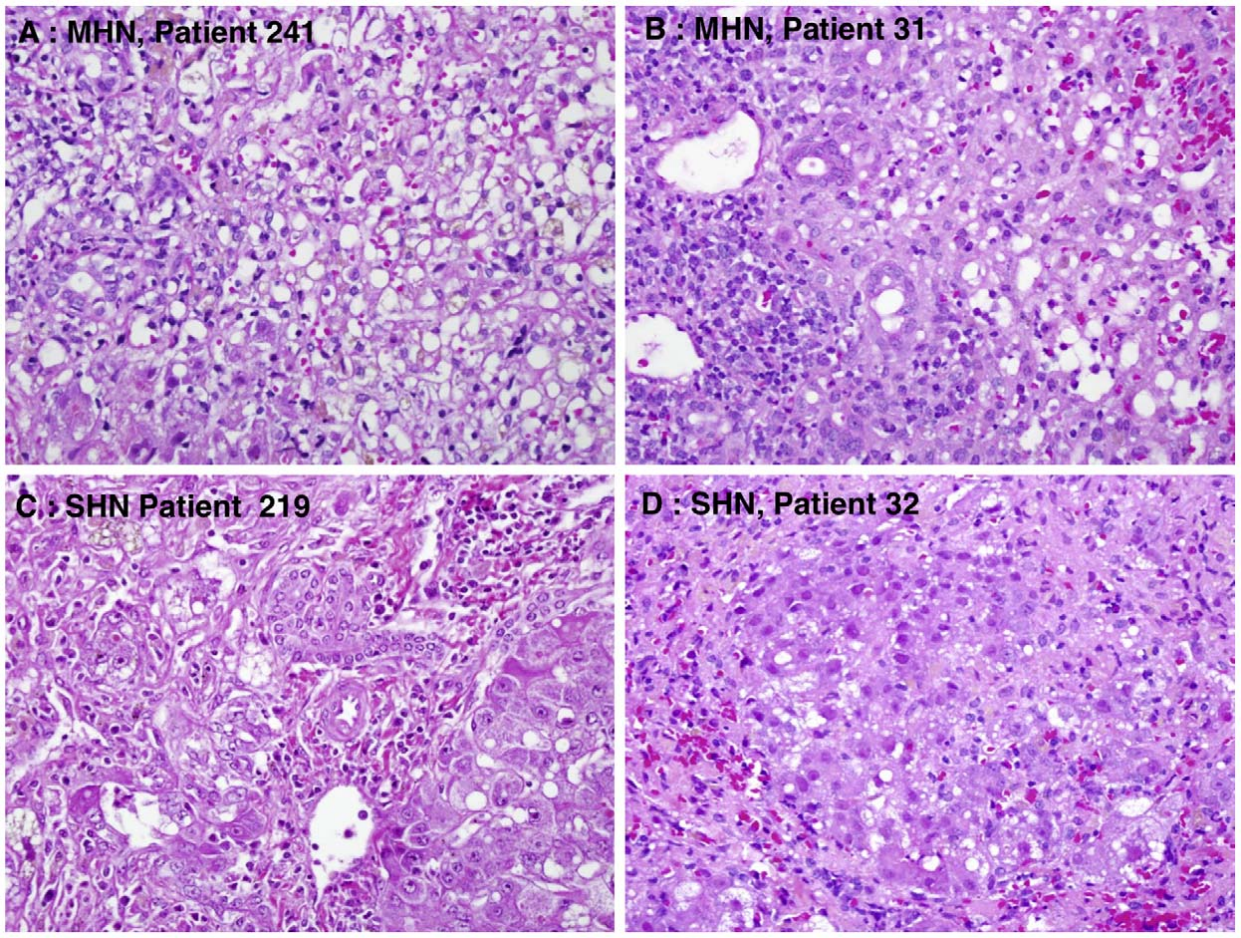
Histopathologic features of HBV-associated acute liver failure (ALF) showing the severity of hepatic necrosis in 4 patients. At the time of liver transplantation there was massive hepatic necrosis (MHN) with no viable hepatocytes in Patient 241, MHN (nearly 100%) with few scattered hepatocytes in Patient 31, and submassive hepatic necrosis (SHN) in Patients 219 (70 to 80% necrosis) and 32 (60% necrosis) (hematoxylin and eosin, x400).

**Figure 2:**
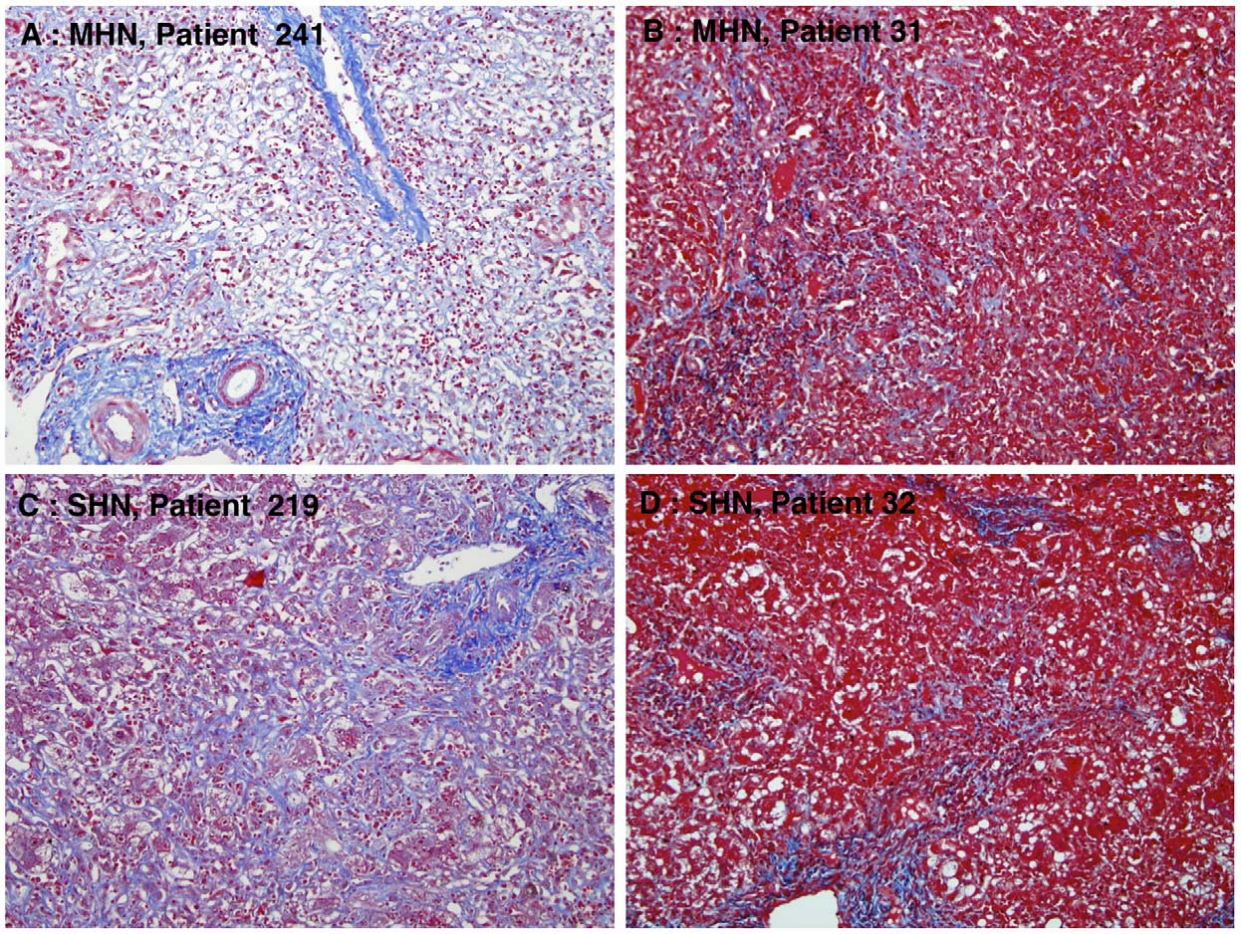
Liver fibrosis in HBV-associated ALF. Delicate fibrosis is evident in the zones of collapsed necrotic parenchyma. The mature collagen of portal areas stains dark blue, while the newer collagen stains light blue (Masson’s trichrome, x200).

The native livers were analyzed macroscopically. Their size was reduced (range, from 1,090 g to 1,160 g), and all appeared smooth without nodules and well preserved. Microscopic examination of the native liver revealed massive hepatic necrosis (MHN) with no viable hepatocytes in Patient 241, MHN (nearly 100%) with few scattered hepatocytes in Patient 31, and submassive hepatic necrosis (SHN) in Patients 219 (70 to 80% necrosis) and 32 (60% necrosis) (Figure 1). In cases with submassive necrosis there was hepatocellular preservation mainly in zone 1, with necrosis of zone 2 and 3 hepatocytes. Case 32 showed early regenerative nodule formation. All cases showed moderate portal inflammation consisting of lymphocytes and plasma cells, while in the necrotic areas there was a prominent infiltrate of hypertrophied, pigmented macrophages with scattered lymphocytes. All patients showed ductular reaction mainly confined to zone 1. Review of the Masson trichrome stains showed evidence of early and delicate parenchymal fibrosis in the necrotic zones of all cases (Figure 2).

### Gene Expression Profiling

**Figure 3:**
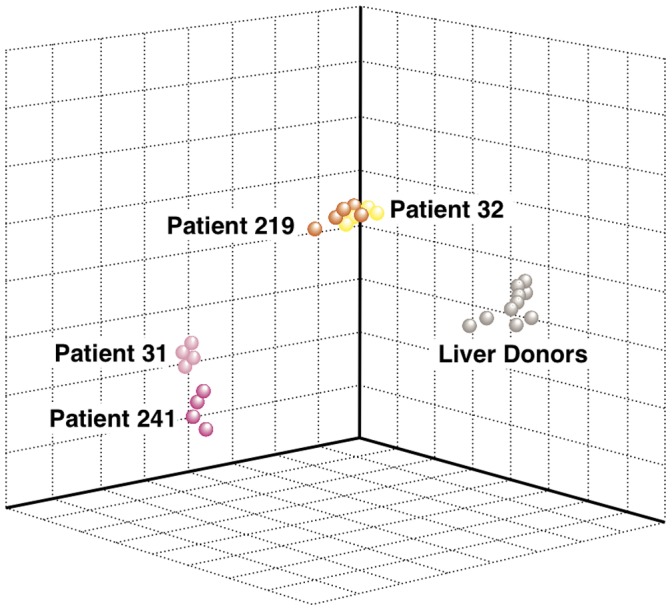
Multidimensional scaling. Three-dimensional (3D) projection of 17 liver specimens from 4 patients with ALF and 10 specimens from individual liver donors by multidimensional scaling using all 11,597 transcripts that passed the filtering criteria. In the 3D projection, each point represents an individual liver specimen, and the distance between points is proportional to the overall dissimilarity of gene expression profiling. The plot illustrates how the gene expression profiles differentiate between ALF and liver donors, as well as among ALF patients. ALF patients with massive hepatic necrosis (MHN) (Patients 241 and 31) and those with submassive hepatic necrosis (SHN) (Patients 219 and 32) form two distinct clusters whose distance from liver donors reflects the extent of liver injury.

**Figure 4:**
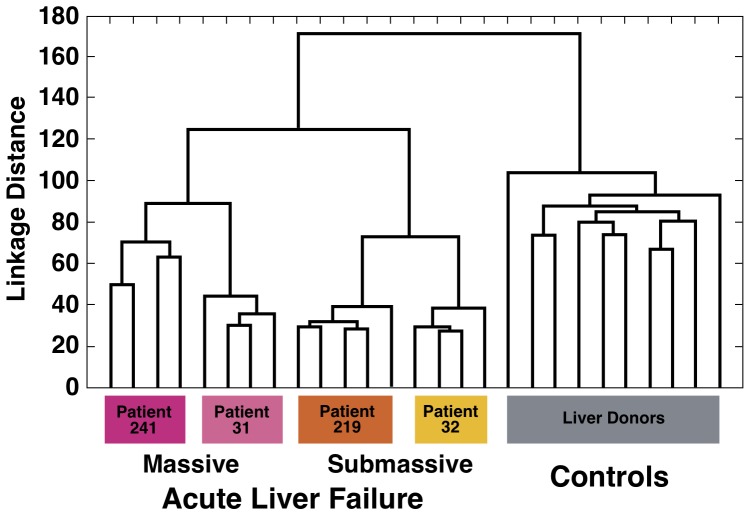
Hierarchical clustering. Hierarchical cluster analysis of 17 liver specimens from 4 patients with acute liver failure and 10 specimens from individual liver donors using all 11,597 transcripts that passed the filtering criteria. All specimens were grouped into 3 main clusters, which correspond to the 3 liver conditions (ALF with MHN, ALF with SHN and liver donors). The unsupervised nature of the analysis and the 100% correct classification of samples suggest a high specificity and sensitivity of gene expression differences between ALF and normal livers.

**Figure 5:**
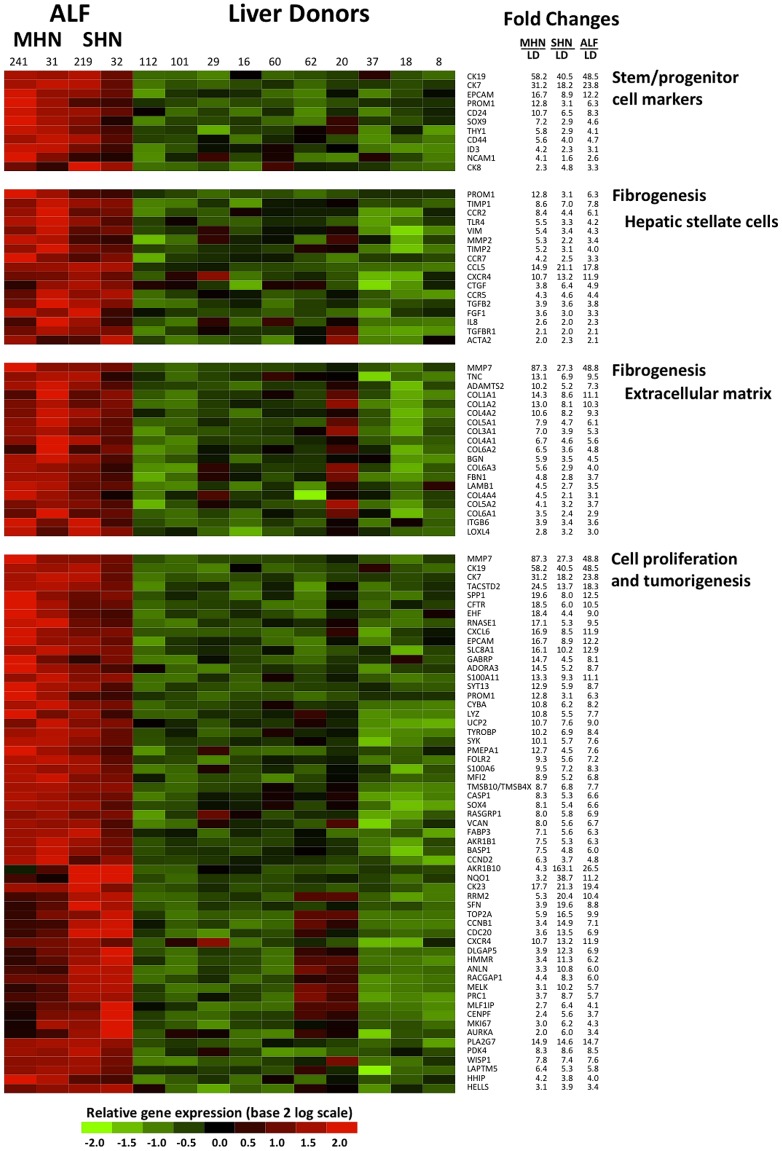
Differential gene expression between HBV-associated ALF and liver donors showing the up-regulated transcripts related to liver regeneration. Data of the 10 liver donors represent individual samples, whereas data of the 4 ALF patients represent the average of multiple samples analyzed (up to 5 liver specimens for each patient). Each cell represents the expression of a particular transcript (rows) of a particular liver specimen (columns). The color in each cell reflects the level of expression of the corresponding gene in the corresponding sample, relative to its mean level of expression in the entire set of 14 samples. Ratios were log2-transformed and row-wise standardized. According to the color scale, up-regulated genes are shown in shades of red, down-regulated genes in shades of green. MHN denotes massive hepatic necrosis. SHN denotes submassive hepatic necrosis. LD denotes liver donors.

The unsupervised MDS of the 27 liver specimens demonstrated a complete separation between ALF and liver donors (Figure 3). Remarkably, when different liver specimens from the same patient were analyzed, they were invariably found to be clustered next to each other. However, among patients with ALF, those with SHN (60% to 80% necrosis) formed a separate cluster intermediate between liver donors and patients with MHN (nearly 100% necrosis). Thus, there was a correlation between the degree of liver necrosis and the separation observed by MDS. Importantly, these data were confirmed by the unsupervised hierarchical clustering, based on all 11,597 transcripts that passed the filtering criteria. Although no information on the identity of the samples was used in the clustering, the algorithm showed an ordination of all 27 individual liver specimens in perfect agreement with the degree of histopathological severity (MHN and SHN and liver donors) (Figure 4). Multivariate permutation analysis with a false discovery rate of <1% identified 4,553 transcripts differentially expressed in ALF, which corresponded to 2,533 unique genes. Of these, 1,385 were up-regulated and 1,148 were down-regulated (Tables S1 and S2). The 2,533 unique genes were considered for functional analysis using Ingenuity and Gene Ontology. The main focus of this study was to define a gene signature of liver regeneration by using gene expression profiling. Genes associated with HSPC, liver fibrogenesis, cell growth and proliferation, and tumorigenesis were highly represented and overexpressed in ALF versus liver donors (Figure 5). Consistent with the ductular reaction seen in both MHN and SHN, we found expression of HSPC markers [6], [7]. The most up-regulated were CK19 and CK7, EPCAM, CD133/PROM1, CD24, SOX9, THY1, CD44, NCAM1, and CK8. Interestingly, although these genes were found to be up-regulated in each of the four patients, those with MHN had the greatest up-regulation of stem/progenitor cell markers (up to 58 fold changes) (Figure 5), while patient 32, with the lowest extent of liver necrosis, had the lowest degree of up-regulation. Only CK8 was found to be more up-regulated in SHN. Another gene that stimulates fetal hepatoblast proliferation and may contribute to tumorigenesis, the transcriptional regulator Id3 [18], [19], was found to be up-regulated especially in MHN. Consistent with previous studies in patients with acute and chronic liver diseases [20] and in mouse models [21], our data also showed up-regulated genes related to the Wnt pathway (FZD2, SFRP, LEF1) [22] and the Notch pathway (MAML2, HEYL, JAG1, and DXT3) [23].

Concurrent with HSPC activation, in all ALF patients we observed the expression of a remarkable number of up-regulated genes associated with liver fibrogenesis (Figure 5). These comprised a high number of genes related to activation of HSC [24], which are major producers of collagen and key players in liver fibrogenesis [25]. These include specific chemokines and chemokine receptors [24], [25], such as CCL5, IL8, CCR2, CCR5, CCR7, and CXCR4, cytokine and cytokine receptors (TGFB2, TGFBR1), and toll-like receptor 4 (TLR4) (Figure 5). Up-regulated genes produced by HSC include matrix metalloproteinase 2 (MMP-2) and tissue inhibitors of matrix metalloproteinases (TIMP-1 and TIMP-2), which are responsible for preventing the degradation of the fibrotic liver matrix [24]. In addition, there was a marked up-regulation of CD133/PROM1, a gene initially associated with HSPC but more recently shown to be also expressed by a subset of HSC [26]. Several of these HSC-associated genes (CD133/PROM1, TIMP1, CCR2, TLR4, MMP2, TIMP2, and CCR7, ACTA2/αSMA, VIM) had a greater fold change in MHN than in SHN (Figure 5). Consistent with HSC activation, positive staining for smooth-muscle actin (αSMA) was observed by immunohistochemistry in the necrotic parenchyma and around proliferating ductules (data not shown). Extracellular matrix genes were also highly expressed, including two key molecules of liver fibrogenesis, COL1A1 and COL1A2, as well as additional collagen and extracellular matrix components [25]. MMP-7, a gene found overexpressed in idiopathic pulmonary and liver fibrosis [27], [28], was also among the up-regulated, most prominently in patient 241 with MHN. Our data show that all genes related to hepatic fibrogenesis were more highly expressed in MHN than in SHN.

Interestingly, we found in ALF an abundant signature of genes related to cell growth, proliferation and tumorigenesis, with a total number of 937 genes identified by Ingenuity. In MHN, the most up-regulated genes (with a fold-change >10) included several genes that also identify hepatic cancer stem cells, most notably EpCAM, CK19 and CK7 (Figure 5) [29]. Additional genes related to hepatic cancer stem cells were more expressed in MHN than in SHN, although their fold-changes were lower than 10 (CD133, THY-1, CD44) [29]. Conversely, the most up-regulated genes (with a fold change >10) in SHN comprised genes related to cell growth and proliferation (AKR1B10, NQO1, CK23, RRM2, SFN, TOP2A, CCNB1, CDC20, CXCR4, DLGAP5, HMMR, ANLN and MELK) (Figure 5). Likewise, genes related to cell proliferation, such as RACGAP1, PRC1, MLF1IP, KI67, AURKA and CENPF were more expressed in SHN than in MHN, but their fold-changes were lower than 10. Ki67, a typical marker of proliferation [30] was up-regulated in all ALF cases, with the greatest up-regulation in patient 32, who had evidence of early regenerative nodules (Figure 1D). Consistent with an ongoing liver regeneration effort, several growth factors were also up-regulated, including CTGF [31], FGF [6] and GRN [32]. A complete list of the up- and down-regulated genes is reported in Tables S1 and S2.

## Discussion

Studies of liver regeneration following acute liver injury in humans have been limited and mostly focused on events that occur after partial hepatectomy, when mature hepatocyte proliferation is the primary driver of liver repopulation [1], [12]. The availability of multiple specimens from the explanted livers of 4 patients who developed HBV-associated ALF gave us a unique opportunity to study the role and extent of HSPC proliferation in liver regeneration associated with this dramatic clinical syndrome. By combining gene expression profiling with liver pathology and clinical data, we provide a molecular definition of liver regeneration following HBV-associated ALF in humans. Although the number of patients included in this study was limited and only a single time point was available for study (at the time of OLT when the native liver was explanted), all patients included had ALF due to the same etiology, all underwent OLT within one week of the clinical onset, and for each of them we analyzed up to 5 liver specimens by microarray and liver histology. Remarkably, we found that different liver specimens from the same patient clustered together when analyzed by MDS and clustering analysis, suggesting the lack of important individual variances among different liver specimens obtained from the same patient.

Gene expression analysis documented a distinct signature associated with ALF. Strikingly, however, when we analyzed all 11,570 genes that passed the filtering criteria by using an unsupervised clustering analysis and MDS, we identified two well-defined clusters that segregated according to the histopathological severity, i.e., MHN vs. SHN. It is important to emphasize that the majority of differentially expressed genes were shared by the two clusters, but they varied in their intensity of expression following a gradient from MHN to SHN to liver donors. One of the most prominent features of the gene signature of HBV-associated ALF was the activation of HSPC, reflecting the extreme conditions under which liver regeneration must occur following massive destruction of the hepatic parenchyma. Interestingly, in agreement with the separation observed between MHN and SHN by MDS and clustering analysis, genes associated with HSPC, most notably CK19, CK7, EPCAM, CD133/PROM1, CD24, and SOX9, showed the highest expression in MHN, whereas the patient with the lowest degree of liver necrosis had the least up-regulation. These findings were corroborated by histopathology, which documented a remarkable ductular reaction in MHN without evidence of hepatocyte-like cells. Thus, our data demonstrate that the strong ductular reaction present in MHN was not associated with further differentiation toward hepatocyte-like cells, a finding also supported by the lack of alpha-fetoprotein gene expression in the liver, as well as by the lack of alpha-fetoprotein increase in serum. In contrast, in SHN besides a prominent ductular reaction, we observed the presence of early regenerative nodules with a hepatocyte-like morphology, reflecting an intermediate phenotype between progenitor cells and hepatocytes. Moreover, the association of intermediate hepatocyte-like cells with elevated serum alpha-fetoprotein levels in Patient 32 reflects the importance of this marker in liver regeneration, as previously reported [33]. Recent studies have reported that, in patients with acute and chronic liver disease [20] and in mouse models [21], the Wnt and Notch signaling pathways are important in the regulation of HSPC being implicated in the lineage specification of hepatocytes and cholangiocytes, respectively, during the process of liver regeneration [34]. In ALF, we found up-regulated genes associated with the Wnt [22] and Notch [23] pathways, although key components of these pathways were not detected.

Another major finding of our study was a strong gene signature of hepatic fibrogenesis in ALF. Interestingly, genes associated with HSC activation and extracellular matrix components were again more up-regulated in MHN than in SHN, indicating that the extent of hepatic fibrosis positively correlated with the histopathological severity. Consistent with our gene expression profiles, recent data by immunohistochemistry have documented HSC activation in patients with ALF, along with an increase in serum levels of matrix metalloproteinase (MMP-1), MMP-2, MMP-9, and tissue inhibitors of MMP-1 (TIMP-1), TIMP-2 [35]. Strikingly, MMP-7, whose expression has been found up-regulated in idiopathic pulmonary fibrosis [27] as well as in biliary atresia-associated liver fibrosis [28], was the most up-regulated gene in MHN. However, evidence is accumulating to suggest that MMP-7, like other MMPs, has also a major role in cancer [36], although it has also found to be expressed by oval cells in rats models [37]. It has been recently hypothesized that the occurrence of fibrosis in ALF may be a physiological and possibly beneficial response of the liver to support the collapsing parenchymal structure through the deposition of new extracellular matrix [35]. Our study confirms and further extends this concept providing evidence that the degree of liver fibrosis positively correlates with the histopathological severity. Indeed, the highest expression of genes related to HSC activation and extracellular matrix was detected in patients with MHN.

Cancer has been described as a wound that does not heal [38]. Previous studies have investigated similarities and differences between tissue regeneration (wound healing) and tumorigenesis [38], [39]. A remarkable feature that we documented in ALF was an overriding tumorigenesis gene signature. Functional analysis using IPA identified more than 900 differentially expressed genes related to cell growth, proliferation and tumorigenesis, most of which were never previously associated with liver regeneration. Interestingly, our analysis also provided evidence that the most-up-regulated genes in MHN reflect a stem/progenitor cell signature, which shares phenotypic traits with cancer stem cells [40], whereas the most up-regulated genes in SHN likely reflect proliferation of more committed hepatocyte-like cells as those present in the early regenerative nodules. One of the most up-regulated genes in ALF, more prominently expressed in SHN, was AKR1B10, a detoxifying enzyme associated with the control of cell growth and proliferation [41]. Although the molecular mechanisms underlying its role in cell proliferation and cancer have yet to be elucidated, this gene was found overexpressed in several tumors [42] including hepatocellular carcinoma [43]. The identification of common genes between liver regeneration and liver cancer underlines the overlaps in the biology and regulation of these processes, emphasizing the need to further dissect the role of these genes as well as the role of stem and progenitor cells in both of these settings [44].

In summary, gene expression profiling provided new insights into the molecular pathogenesis of liver regeneration in humans. Our study demonstrated that liver regeneration in ALF is characterized by a prominent ductular reaction both in MHN and in SHN, associated with extensive fibrogenesis to repair damaged liver tissue. However, the development of grade IV coma within a few days from the onset of clinical symptoms required an immediate OLT, which prevented the evaluation of whether the regenerative response would have resulted in an effective liver regeneration. Whereas in MHN we have observed a marked HSPC gene signature with no evidence of further differentiation, in SHN the HSPC signature was associated with evidence of more committed hepatocyte-like cells and evidence of highly expressed genes associated with cell growth and proliferation, as well as tumorigenesis, most of them never reported in liver regeneration. Our data provided evidence that the intensity of the gene signature in HBV-associated ALF is directly correlated with the histopathological severity, raising the question whether molecular analysis of tissue samples obtained from patients with ALF prior to transplant could provide prognostic information. Finally, the results of our study underline the importance of dissecting the relationship between liver regeneration and liver cancer.

## Supporting Information

Table S1 **Up-regulated Transcripts in HBV-Associated Acute Liver Failure.**
(DOCX)

Table S2 **Down-regulated Transcripts in HBV-Associated Acute Liver Failure.**
(DOCX)
