# Supplementary material for: Liver Regeneration Signature in Hepatitis B Virus (HBV)-Associated Acute Liver Failure Identified by Gene Expression Profiling
Source: PLoS One. 2012 Nov 21;7(11):e49611. doi: 10.1371/journal.pone.0049611 (PMC3504149; doi:10.1371/journal.pone.0049611)
Supplement: Table S1 — Up-regulated Transcripts in HBV-Associated Acute Liver Failure. (DOCX) [file pone.0049611.s001.docx]

| **Table S1. Up-regulated Transcripts in HBV-Associated Acute Liver Failure** | | | | | |
| --- | --- | --- | --- | --- | --- |
|  |  |  |  |  |  |
|  |  |  | **Fold Change** | | |
| **Affymetrix Probe ID** | **Transcript Name** | **Gene Symbol** | **MHN/LD** | **SHN/LD** | **All ALF Cases/LD** |
| 204259_at | matrix metallopeptidase 7 (matrilysin, uterine) | MMP7 | 87.3 | 27.3 | 48.8 |
| 201650_at | keratin 19 | KRT19 | 58.2 | 40.5 | 48.5 |
| 215176_x_at | immunoglobulin kappa locus  immunoglobulin kappa constant | IGK@  IGKC | 40.9 | 24.4 | 31.6 |
| 209374_s_at | immunoglobulin heavy constant mu | IGHM | 39.6 | 23.2 | 30.3 |
| 206561_s_at | aldo-keto reductase family 1, member B10 (aldose reductase) | AKR1B10 | 4.3 | 163.1 | 26.5 |
| 209016_s_at | keratin 7 | KRT7 | 31.2 | 18.2 | 23.8 |
| 205267_at | POU class 2 associating factor 1 | POU2AF1 | 29.4 | 17.7 | 22.8 |
| 216401_x_at | partial IGKV gene for immunoglobulin kappa chain variable region, clone 38* | IGKV | 28.5 | 15.0 | 20.7 |
| 218963_s_at | keratin 23 (histone deacetylase inducible) | KRT23 | 17.7 | 21.3 | 19.4 |
| 231647_s_at | Fc receptor-like 5 | FCRL5 | 27.9 | 13.4 | 19.3 |
| 202286_s_at | tumor-associated calcium signal transducer 2 | TACSTD2 | 24.5 | 13.7 | 18.3 |
| 223565_at | plasma cell-induced ER protein 1 | MGC29506 | 23.9 | 13.7 | 18.1 |
| 34210_at | CD52 molecule | CD52 | 22.0 | 14.8 | 18.1 |
| 1569040_s_at | ankyrin repeat domain 36B pseudogene 2 | ANKRD36BP2 | 26.1 | 12.2 | 17.9 |
| 1405_i_at | chemokine (C-C motif) ligand 5 | CCL5 | 14.9 | 21.1 | 17.8 |
| 217179_x_at | mRNA for IG lambda light chain* | IGL | 25.6 | 11.5 | 17.1 |
| 202953_at | complement component 1, q subcomponent, B chain | C1QB | 19.8 | 12.8 | 15.9 |
| 209138_x_at | Immunoglobulin lambda locus | IGL@ | 15.9 | 14.5 | 15.2 |
| 203290_at | major histocompatibility complex, class II, DQ alpha 1 | HLA-DQA1 | 15.5 | 14.6 | 15.1 |
| 210164_at | granzyme B (granzyme 2, cytotoxic T-lymphocyte-associated serine esterase 1) | GZMB | 10.7 | 20.5 | 14.8 |
| 206214_at | phospholipase A2, group VII (platelet-activating factor acetylhydrolase, plasma) | PLA2G7 | 14.9 | 14.6 | 14.7 |
| 213566_at | ribonuclease, RNase A family, k6 | RNASE6 | 18.1 | 10.3 | 13.7 |
| 1561615_s_at | solute carrier family 8 (sodium/calcium exchanger), member 1 | SLC8A1 | 16.1 | 10.2 | 12.9 |
| 211798_x_at | immunoglobulin lambda joining 3 | IGLJ3 | 14.8 | 11.1 | 12.8 |
| 212998_x_at | major histocompatibility complex, class II, DQ beta 1  HLA class II histocompatibility antigen, DQ beta 1 chain-like | HLA-DQB1  LOC100133583 | 11.7 | 13.6 | 12.6 |
| 217235_x_at | immunoglobulin lambda-like polypeptide 5 immunoglobulin lambda variable 2-11 | IGLL5  IGLV2-11 | 13.0 | 12.3 | 12.6 |
| 218232_at | complement component 1, q subcomponent, A chain | C1QA | 15.6 | 10.1 | 12.6 |
| 209875_s_at | secreted phosphoprotein 1 | SPP1 | 19.6 | 8.0 | 12.5 |
| 205488_at | granzyme A (granzyme 1, cytotoxic T-lymphocyte-associated serine esterase 3) | GZMA | 11.9 | 12.8 | 12.3 |
| 201839_s_at | epithelial cell adhesion molecule | EPCAM | 16.7 | 8.9 | 12.2 |
| 214974_x_at | chemokine (C-X-C motif) ligand 5 | CXCL5 | 11.7 | 12.7 | 12.2 |
| 235751_s_at | vitelline membrane outer layer 1 homolog (chicken) | VMO1 | 14.2 | 10.2 | 12.0 |
| 206336_at | chemokine (C-X-C motif) ligand 6 (granulocyte chemotactic protein 2) | CXCL6 | 16.9 | 8.5 | 11.9 |
| 217028_at | chemokine (C-X-C motif) receptor 4 | CXCR4 | 10.7 | 13.2 | 11.9 |
| 219386_s_at | SLAM family member 8 | SLAMF8 | 15.9 | 8.7 | 11.8 |
| 201141_at | glycoprotein (transmembrane) nmb | GPNMB | 11.7 | 11.6 | 11.6 |
| 204787_at | V-set and immunoglobulin domain containing 4 | VSIG4 | 15.4 | 8.5 | 11.4 |
| 212592_at | immunoglobulin J polypeptide, linker protein for immunoglobulin alpha and mu polypeptides | IGJ | 13.3 | 9.5 | 11.3 |
| 201137_s_at | major histocompatibility complex, class II, DP beta 1 | HLA-DPB1 | 11.3 | 11.1 | 11.2 |
| 206641_at | tumor necrosis factor receptor superfamily, member 17 | TNFRSF17 | 19.2 | 6.6 | 11.2 |
| 210519_s_at | NAD(P)H dehydrogenase, quinone 1 | NQO1 | 3.2 | 38.7 | 11.2 |
| 200660_at | S100 calcium binding protein A11 | S100A11 | 13.3 | 9.3 | 11.1 |
| 202310_s_at | collagen, type I, alpha 1 | COL1A1 | 14.3 | 8.6 | 11.1 |
| 217478_s_at | major histocompatibility complex, class II, DM alpha | HLA-DMA | 12.4 | 9.9 | 11.1 |
| 211990_at | major histocompatibility complex, class II, DP alpha 1 | HLA-DPA1 | 10.8 | 11.2 | 11.0 |
| 215193_x_at | major histocompatibility complex, class II, DR beta 1  major histocompatibility complex, class II, DR beta 3  major histocompatibility complex, class II, DR beta 4 | HLA-DRB1 HLA-DRB3 HLA-DRB4 | 11.3 | 10.3 | 10.8 |
| 214768_x_at | immunoglobulin kappa variable 1-5 | IGKV1-5 | 13.9 | 8.3 | 10.7 |
| 202206_at | ADP-ribosylation factor-like 4C | ARL4C | 13.1 | 8.4 | 10.5 |
| 205043_at | cystic fibrosis transmembrane conductance regulator (ATP-binding cassette sub-family C, member 7) | CFTR | 18.5 | 6.0 | 10.5 |
| 216207_x_at | immunoglobulin kappa constant  immunoglobulin kappa variable 1-5  immunoglobulin kappa variable 1D-8  ig kappa chain V-I region HK102-like  similar to Ig kappa chain V-I region HK102 precursor | IGKC  IGKV1-5  IGKV1D-8  LOC652493 LOC652694 | 13.0 | 8.4 | 10.5 |
| 209773_s_at | ribonucleotide reductase M2 | RRM2 | 5.3 | 20.4 | 10.4 |
| 202404_s_at | collagen, type I, alpha 2 | COL1A2 | 13.0 | 8.1 | 10.3 |
| 215946_x_at | immunoglobulin lambda-like polypeptide 3, pseudogene | IGLL3P | 10.2 | 10.3 | 10.3 |
| 225681_at | collagen triple helix repeat containing 1 | CTHRC1 | 14.6 | 7.3 | 10.3 |
| 212311_at | sel-1 suppressor of lin-12-like 3 (C. elegans) | SEL1L3 | 11.7 | 8.6 | 10.0 |
| 201291_s_at | topoisomerase (DNA) II alpha 170kDa | TOP2A | 5.9 | 16.5 | 9.9 |
| 219505_at | cat eye syndrome chromosome region, candidate 1 | CECR1 | 9.8 | 9.7 | 9.8 |
| 202075_s_at | phospholipid transfer protein | PLTP | 13.6 | 6.9 | 9.7 |
| 201645_at | tenascin C | TNC | 13.1 | 6.9 | 9.5 |
| 201785_at | ribonuclease, RNase A family, 1 (pancreatic) | RNASE1 | 17.1 | 5.3 | 9.5 |
| 218729_at | latexin | LXN | 12.9 | 7.0 | 9.5 |
| 206380_s_at | complement factor properdin | CFP | 11.3 | 7.9 | 9.4 |
| 211964_at | collagen, type IV, alpha 2 | COL4A2 | 10.6 | 8.2 | 9.3 |
| 205382_s_at | complement factor D (adipsin) | CFD | 9.9 | 8.4 | 9.1 |
| 223344_s_at | membrane-spanning 4-domains, subfamily A, member 7 | MS4A7 | 11.2 | 7.4 | 9.1 |
| 208998_at | uncoupling protein 2 (mitochondrial, proton carrier) | UCP2 | 10.7 | 7.6 | 9.0 |
| 210146_x_at | leukocyte immunoglobulin-like receptor, subfamily B (with TM and ITIM domains), member 2 | LILRB2 | 10.2 | 7.9 | 9.0 |
| 225645_at | ets homologous factor | EHF | 18.4 | 4.4 | 9.0 |
| 226878_at | major histocompatibility complex, class II, DO alpha | HLA-DOA | 9.7 | 8.1 | 8.9 |
| 228532_at | chromosome 1 open reading frame 162 | C1orf162 | 11.7 | 6.8 | 8.9 |
| 206584_at | lymphocyte antigen 96 | LY96 | 11.9 | 6.5 | 8.8 |
| 222925_at | doublecortin domain containing 2 | DCDC2 | 12.6 | 6.2 | 8.8 |
| 242665_at | formin-like 2 | FMNL2 | 10.0 | 7.7 | 8.8 |
| 33323_r_at | stratifin | SFN | 3.9 | 19.6 | 8.8 |
| 223660_at | adenosine A3 receptor | ADORA3 | 14.5 | 5.2 | 8.7 |
| 226086_at | synaptotagmin XIII | SYT13 | 12.9 | 5.9 | 8.7 |
| 1562321_at | pyruvate dehydrogenase kinase, isozyme 4 | PDK4 | 8.3 | 8.6 | 8.5 |
| 209619_at | CD74 molecule, major histocompatibility complex, class II invariant chain | CD74 | 8.4 | 8.5 | 8.5 |
| 204122_at | TYRO protein tyrosine kinase binding protein | TYROBP | 10.2 | 6.9 | 8.4 |
| 217022_s_at | immunoglobulin heavy constant alpha 1  immunoglobulin heavy constant alpha 2 (A2m marker) hypothetical LOC100126583 | IGHA1  IGHA2 LOC100126583 | 7.4 | 9.5 | 8.4 |
| 208650_s_at | CD24 molecule | CD24 | 10.7 | 6.5 | 8.3 |
| 217728_at | S100 calcium binding protein A6 | S100A6 | 9.5 | 7.2 | 8.3 |
| 219452_at | dipeptidase 2 | DPEP2 | 10.5 | 6.6 | 8.3 |
| 244313_at | complement component (3b/4b) receptor 1 (Knops blood group) | CR1 | 10.6 | 6.4 | 8.3 |
| 203028_s_at | cytochrome b-245, alpha polypeptide | CYBA | 10.8 | 6.2 | 8.2 |
| 205044_at | gamma-aminobutyric acid (GABA) A receptor, pi | GABRP | 14.7 | 4.5 | 8.1 |
| 206295_at | interleukin 18 (interferon-gamma-inducing factor) | IL18 | 7.9 | 8.4 | 8.1 |
| 209606_at | cytohesin 1 interacting protein | CYTIP | 7.6 | 8.7 | 8.1 |
| 219159_s_at | SLAM family member 7 | SLAMF7 | 10.1 | 6.5 | 8.1 |
| 219519_s_at | sialic acid binding Ig-like lectin 1, sialoadhesin | SIGLEC1 | 12.5 | 5.3 | 8.1 |
| 235885_at | purinergic receptor P2Y, G-protein coupled, 12 | P2RY12 | 12.5 | 5.3 | 8.1 |
| 205819_at | macrophage receptor with collagenous structure | MARCO | 9.8 | 6.5 | 8.0 |
| 209626_s_at | oxysterol binding protein-like 3 | OSBPL3 | 9.2 | 7.0 | 8.0 |
| 225353_s_at | complement component 1, q subcomponent, C chain | C1QC | 8.1 | 7.8 | 8.0 |
| 208894_at | major histocompatibility complex, class II, DR alpha | HLA-DRA | 7.4 | 8.4 | 7.9 |
| 218002_s_at | chemokine (C-X-C motif) ligand 14 | CXCL14 | 14.5 | 4.3 | 7.9 |
| 219607_s_at | membrane-spanning 4-domains, subfamily A, member 4 | MS4A4A | 9.8 | 6.4 | 7.9 |
| 201666_at | TIMP metallopeptidase inhibitor 1 | TIMP1 | 8.6 | 7.0 | 7.8 |
| 205831_at | CD2 molecule | CD2 | 8.1 | 7.5 | 7.8 |
| 209924_at | chemokine (C-C motif) ligand 18 (pulmonary and activation-regulated) | CCL18 | 9.0 | 6.8 | 7.8 |
| 1555745_a_at | lysozyme | LYZ | 10.8 | 5.5 | 7.7 |
| 217733_s_at | thymosin beta 10 | TMSB10 | 8.7 | 6.8 | 7.7 |
| 203932_at | major histocompatibility complex, class II, DM beta | HLA-DMB | 8.8 | 6.6 | 7.6 |
| 204205_at | apolipoprotein B mRNA editing enzyme, catalytic polypeptide-like 3G | APOBEC3G | 10.2 | 5.7 | 7.6 |
| 204912_at | interleukin 10 receptor, alpha | IL10RA | 10.0 | 5.8 | 7.6 |
| 219768_at | V-set domain containing T cell activation inhibitor 1 | VTCN1 | 10.5 | 5.5 | 7.6 |
| 222450_at | prostate transmembrane protein, androgen induced 1 | PMEPA1 | 12.7 | 4.5 | 7.6 |
| 226068_at | spleen tyrosine kinase | SYK | 10.1 | 5.7 | 7.6 |
| 227314_at | integrin, alpha 2 (CD49B, alpha 2 subunit of VLA-2 receptor) | ITGA2 | 8.3 | 7.0 | 7.6 |
| 228273_at | proline rich 11 | PRR11 | 6.2 | 9.2 | 7.6 |
| 229802_at | --- | --- | 7.8 | 7.4 | 7.6 |
| 229937_x_at | Leukocyte immunoglobulin-like receptor, subfamily B (with TM and ITIM domains), member 1 | LILRB1 | 9.0 | 6.4 | 7.6 |
| 206420_at | immunoglobulin superfamily, member 6 | IGSF6 | 9.0 | 6.2 | 7.4 |
| 235458_at | hepatitis A virus cellular receptor 2 | HAVCR2 | 7.3 | 7.5 | 7.4 |
| 207571_x_at | chromosome 1 open reading frame 38 | C1orf38 | 7.9 | 6.7 | 7.3 |
| 213915_at | natural killer cell group 7 sequence | NKG7 | 7.7 | 6.9 | 7.3 |
| 225105_at | chromosome 12 open reading frame 75 | C12orf75 | 8.9 | 5.9 | 7.3 |
| 226311_at | ADAM metallopeptidase with thrombospondin type 1 motif, 2 | ADAMTS2 | 10.2 | 5.2 | 7.3 |
| 227480_at | sushi domain containing 2 | SUSD2 | 10.6 | 5.1 | 7.3 |
| 204829_s_at | folate receptor 2 (fetal) | FOLR2 | 9.3 | 5.6 | 7.2 |
| 205495_s_at | granulysin | GNLY | 7.0 | 7.5 | 7.2 |
| 210321_at | granzyme H (cathepsin G-like 2, protein h-CCPX) | GZMH | 6.2 | 8.4 | 7.2 |
| 216557_x_at | immunoglobulin heavy constant alpha 1  immunoglobulin heavy constant delta  immunoglobulin heavy constant gamma 1 (G1m marker) immunoglobulin heavy constant gamma 3 (G3m marker) immunoglobulin heavy constant mu  immunoglobulin heavy variable 3-48  immunoglobulin heavy variable 4-31  hypothetical protein LOC100291917 | IGHA1  IGHD  IGHG1  IGHG3  IGHM  IGHV3-48 IGHV4-31  LOC100291917 | 8.9 | 5.8 | 7.2 |
| 225799_at | hypothetical LOC541471  non-protein coding RNA 152 | LOC541471 NCRNA00152 | 7.1 | 7.4 | 7.2 |
| 214710_s_at | cyclin B1 | CCNB1 | 3.4 | 14.9 | 7.1 |
| 228167_at | kelch-like 6 (Drosophila) | KLHL6 | 6.8 | 7.2 | 7.0 |
| 202870_s_at | cell division cycle 20 homolog (S. cerevisiae) | CDC20 | 3.6 | 13.5 | 6.9 |
| 203764_at | discs, large (Drosophila) homolog-associated protein 5 | DLGAP5 | 3.9 | 12.3 | 6.9 |
| 205590_at | RAS guanyl releasing protein 1 (calcium and DAG-regulated) | RASGRP1 | 8.0 | 5.8 | 6.9 |
| 209933_s_at | CD300a molecule | CD300A | 8.2 | 5.8 | 6.9 |
| 227742_at | chloride intracellular channel 6 | CLIC6 | 10.1 | 4.7 | 6.9 |
| 235385_at | membrane-associated ring finger (C3HC4) 1 | MARCH1 | 9.0 | 5.3 | 6.9 |
| 206632_s_at | apolipoprotein B mRNA editing enzyme, catalytic polypeptide-like 3B | APOBEC3B | 6.6 | 7.1 | 6.8 |
| 213160_at | dedicator of cytokinesis 2 | DOCK2 | 8.0 | 5.8 | 6.8 |
| 235911_at | antigen p97 (melanoma associated) identified by monoclonal antibodies 133.2 and 96.5 | MFI2 | 8.9 | 5.2 | 6.8 |
| 203104_at | colony stimulating factor 1 receptor | CSF1R | 8.5 | 5.2 | 6.7 |
| 204891_s_at | lymphocyte-specific protein tyrosine kinase | LCK | 7.2 | 6.3 | 6.7 |
| 209879_at | selectin P ligand | SELPLG | 8.4 | 5.4 | 6.7 |
| 212587_s_at | protein tyrosine phosphatase, receptor type, C | PTPRC | 7.6 | 6.0 | 6.7 |
| 218376_s_at | microtubule associated monoxygenase, calponin and LIM domain containing 1 | MICAL1 | 7.6 | 6.0 | 6.7 |
| 221731_x_at | versican | VCAN | 8.0 | 5.6 | 6.7 |
| 1552703_s_at | caspase recruitment domain family, member 16  caspase 1, apoptosis-related cysteine peptidase (interleukin 1, beta, convertase) | CARD16 CASP1 | 8.3 | 5.3 | 6.6 |
| 1553986_at | RAS and EF-hand domain containing | RASEF | 7.0 | 6.2 | 6.6 |
| 201416_at | SRY (sex determining region Y)-box 4 | SOX4 | 8.1 | 5.4 | 6.6 |
| 205674_x_at | FXYD domain containing ion transport regulator 2 | FXYD2 | 11.7 | 3.8 | 6.6 |
| 205686_s_at | CD86 molecule | CD86 | 8.2 | 5.2 | 6.6 |
| 207651_at | G protein-coupled receptor 171 | GPR171 | 6.2 | 7.1 | 6.6 |
| 210397_at | defensin, beta 1 | DEFB1 | 7.2 | 6.0 | 6.6 |
| 213888_s_at | TRAF3 interacting protein 3 | TRAF3IP3 | 7.6 | 5.7 | 6.6 |
| 214777_at | immunoglobulin kappa variable 4-1 | IGKV4-1 | 7.3 | 6.0 | 6.6 |
| 226818_at | macrophage expressed 1 | MPEG1 | 8.4 | 5.0 | 6.5 |
| 242268_at | CUGBP, Elav-like family member 2 | CELF2 | 6.9 | 6.1 | 6.5 |
| 1552316_a_at | GTPase, IMAP family member 1 | GIMAP1 | 7.8 | 5.2 | 6.4 |
| 1559584_a_at | chromosome 16 open reading frame 54 | C16orf54 | 7.5 | 5.4 | 6.4 |
| 1562255_at | synaptotagmin-like 3 | SYTL3 | 5.0 | 8.3 | 6.4 |
| 202345_s_at | fatty acid binding protein 5 (psoriasis-associated) | FABP5 | 6.1 | 6.6 | 6.4 |
| 202411_at | interferon, alpha-inducible protein 27 | IFI27 | 9.0 | 4.5 | 6.4 |
| 208146_s_at | carboxypeptidase, vitellogenic-like | CPVL | 7.7 | 5.3 | 6.4 |
| 213502_x_at | glucuronidase, beta/immunoglobulin lambda-like polypeptide 1 pseudogene | LOC91316 | 6.9 | 6.0 | 6.4 |
| 224583_at | coactosin-like 1 (Dictyostelium) | COTL1 | 7.5 | 5.5 | 6.4 |
| 201272_at | aldo-keto reductase family 1, member B1 (aldose reductase) | AKR1B1 | 7.5 | 5.3 | 6.3 |
| 204162_at | NDC80 homolog, kinetochore complex component (S. cerevisiae) | NDC80 | 4.5 | 8.9 | 6.3 |
| 204304_s_at | prominin 1 | PROM1 | 12.8 | 3.1 | 6.3 |
| 205798_at | interleukin 7 receptor | IL7R | 7.1 | 5.6 | 6.3 |
| 214285_at | fatty acid binding protein 3, muscle and heart (mammary-derived growth inhibitor) | FABP3 | 6.6 | 5.9 | 6.3 |
| 207165_at | hyaluronan-mediated motility receptor (RHAMM) | HMMR | 3.4 | 11.3 | 6.2 |
| 204232_at | Fc fragment of IgE, high affinity I, receptor for; gamma polypeptide | FCER1G | 7.4 | 5.0 | 6.1 |
| 206978_at | chemokine (C-C motif) receptor 2 | CCR2 | 8.4 | 4.4 | 6.1 |
| 209683_at | family with sequence similarity 49, member A | FAM49A | 6.3 | 5.9 | 6.1 |
| 209949_at | neutrophil cytosolic factor 2 | NCF2 | 7.2 | 5.1 | 6.1 |
| 211794_at | FYN binding protein | FYB | 8.3 | 4.6 | 6.1 |
| 212488_at | collagen, type V, alpha 1 | COL5A1 | 7.9 | 4.7 | 6.1 |
| 225647_s_at | cathepsin C | CTSC | 8.1 | 4.6 | 6.1 |
| 225763_at | RCSD domain containing 1 | RCSD1 | 7.4 | 5.1 | 6.1 |
| 202391_at | brain abundant, membrane attached signal protein 1 | BASP1 | 7.5 | 4.8 | 6.0 |
| 206478_at | KIAA0125 | KIAA0125 | 10.1 | 3.6 | 6.0 |
| 209083_at | coronin, actin binding protein, 1A | CORO1A | 7.1 | 5.2 | 6.0 |
| 222077_s_at | Rac GTPase activating protein 1 | RACGAP1 | 4.4 | 8.3 | 6.0 |
| 222608_s_at | anillin, actin binding protein | ANLN | 3.3 | 10.8 | 6.0 |
| 202901_x_at | cathepsin S | CTSS | 6.2 | 5.5 | 5.9 |
| 215925_s_at | CD72 molecule | CD72 | 5.6 | 6.3 | 5.9 |
| 201721_s_at | lysosomal protein transmembrane 5 | LAPTM5 | 6.4 | 5.3 | 5.8 |
| 204026_s_at | ZW10 interactor | ZWINT | 4.3 | 7.7 | 5.8 |
| 205269_at | lymphocyte cytosolic protein 2 (SH2 domain containing leukocyte protein of 76kDa) | LCP2 | 7.0 | 4.9 | 5.8 |
| 205758_at | CD8a molecule | CD8A | 5.5 | 6.1 | 5.8 |
| 208323_s_at | annexin A13 | ANXA13 | 7.8 | 4.4 | 5.8 |
| 208965_s_at | interferon, gamma-inducible protein 16 | IFI16 | 7.9 | 4.3 | 5.8 |
| 214617_at | perforin 1 (pore forming protein) | PRF1 | 4.7 | 7.0 | 5.8 |
| 218872_at | tescalcin | TESC | 7.5 | 4.4 | 5.8 |
| 219630_at | PDZK1 interacting protein 1 | PDZK1IP1 | 6.9 | 4.8 | 5.8 |
| 222245_s_at | fer-1-like 4 (C. elegans) pseudogene | FER1L4 | 7.1 | 4.7 | 5.8 |
| 225847_at | neutral cholesterol ester hydrolase 1 | NCEH1 | 6.3 | 5.4 | 5.8 |
| 1559263_s_at | peptidylprolyl isomerase (cyclophilin)-like 4  zinc finger CCCH-type containing 12D | PPIL4  ZC3H12D | 7.0 | 4.6 | 5.7 |
| 201008_s_at | thioredoxin interacting protein | TXNIP | 6.7 | 4.8 | 5.7 |
| 201744_s_at | lumican | LUM | 7.7 | 4.2 | 5.7 |
| 203083_at | thrombospondin 2 | THBS2 | 6.7 | 4.9 | 5.7 |
| 204825_at | maternal embryonic leucine zipper kinase | MELK | 3.1 | 10.2 | 5.7 |
| 206785_s_at | killer cell lectin-like receptor subfamily C, member 1  killer cell lectin-like receptor subfamily C, member 2 | KLRC1  KLRC2 | 6.7 | 4.8 | 5.7 |
| 216041_x_at | granulin | GRN | 6.3 | 5.1 | 5.7 |
| 218009_s_at | protein regulator of cytokinesis 1 | PRC1 | 3.7 | 8.7 | 5.7 |
| 223553_s_at | docking protein 3 | DOK3 | 7.6 | 4.4 | 5.7 |
| 226435_at | papilin, proteoglycan-like sulfated glycoprotein | PAPLN | 8.6 | 3.7 | 5.7 |
| 227180_at | ELOVL family member 7, elongation of long chain fatty acids (yeast) | ELOVL7 | 8.0 | 4.1 | 5.7 |
| 202664_at | WAS/WASL interacting protein family, member 1 | WIPF1 | 6.4 | 5.0 | 5.6 |
| 203755_at | budding uninhibited by benzimidazoles 1 homolog beta (yeast) | BUB1B | 3.7 | 8.5 | 5.6 |
| 204319_s_at | regulator of G-protein signaling 10 | RGS10 | 6.5 | 4.9 | 5.6 |
| 205821_at | killer cell lectin-like receptor subfamily K, member 1 | KLRK1 | 5.9 | 5.3 | 5.6 |
| 206680_at | CD5 molecule-like | CD5L | 5.6 | 5.5 | 5.6 |
| 211981_at | collagen, type IV, alpha 1 | COL4A1 | 6.7 | 4.6 | 5.6 |
| 220301_at | coiled-coil domain containing 102B | CCDC102B | 7.5 | 4.2 | 5.6 |
| 221210_s_at | N-acetylneuraminate pyruvate lyase (dihydrodipicolinate synthase) | NPL | 6.8 | 4.6 | 5.6 |
| 222253_s_at | POM121 membrane glycoprotein-like 9, pseudogene | POM121L9P | 7.4 | 4.3 | 5.6 |
| 229860_x_at | chromosome 4 open reading frame 48 | C4orf48 | 6.6 | 4.7 | 5.6 |
| 201037_at | phosphofructokinase, platelet | PFKP | 4.1 | 7.4 | 5.5 |
| 202291_s_at | matrix Gla protein | MGP | 8.3 | 3.7 | 5.5 |
| 203761_at | Src-like-adaptor | SLA | 5.7 | 5.3 | 5.5 |
| 205859_at | lymphocyte antigen 86 | LY86 | 6.6 | 4.5 | 5.5 |
| 210715_s_at | serine peptidase inhibitor, Kunitz type, 2 | SPINT2 | 7.6 | 3.9 | 5.5 |
| 213429_at | bicaudal C homolog 1 (Drosophila) | BICC1 | 6.7 | 4.5 | 5.5 |
| 217763_s_at | RAB31, member RAS oncogene family | RAB31 | 7.0 | 4.4 | 5.5 |
| 219476_at | chromosome 1 open reading frame 116 | C1orf116 | 7.7 | 3.9 | 5.5 |
| 229041_s_at | --- | --- | 7.1 | 4.2 | 5.5 |
| 230422_at | formyl peptide receptor 3 | FPR3 | 6.9 | 4.5 | 5.5 |
| 231747_at | cysteinyl leukotriene receptor 1 | CYSLTR1 | 7.3 | 4.1 | 5.5 |
| 201850_at | capping protein (actin filament), gelsolin-like | CAPG | 7.0 | 4.1 | 5.4 |
| 202957_at | hematopoietic cell-specific Lyn substrate 1 | HCLS1 | 6.4 | 4.5 | 5.4 |
| 204444_at | kinesin family member 11 | KIF11 | 4.3 | 6.9 | 5.4 |
| 204588_s_at | solute carrier family 7 (cationic amino acid transporter, y+ system), member 7 | SLC7A7 | 7.2 | 4.0 | 5.4 |
| 204698_at | interferon stimulated exonuclease gene 20kDa | ISG20 | 5.8 | 5.0 | 5.4 |
| 205927_s_at | cathepsin E | CTSE | 6.4 | 4.5 | 5.4 |
| 208018_s_at | hemopoietic cell kinase | HCK | 6.6 | 4.3 | 5.4 |
| 224451_x_at | Rho GTPase activating protein 9 | ARHGAP9 | 5.9 | 4.9 | 5.4 |
| 226210_s_at | maternally expressed 3 (non-protein coding) | MEG3 | 7.2 | 4.0 | 5.4 |
| 226997_at | ADAM metallopeptidase with thrombospondin type 1 motif, 12 | ADAMTS12 | 7.1 | 4.1 | 5.4 |
| 201852_x_at | collagen, type III, alpha 1 | COL3A1 | 7.0 | 3.9 | 5.3 |
| 202898_at | syndecan 3 | SDC3 | 6.0 | 4.6 | 5.3 |
| 204774_at | ecotropic viral integration site 2A | EVI2A | 6.2 | 4.5 | 5.3 |
| 209369_at | annexin A3 | ANXA3 | 7.0 | 4.0 | 5.3 |
| 212651_at | Rho-related BTB domain containing 1 | RHOBTB1 | 5.6 | 5.0 | 5.3 |
| 215049_x_at | CD163 molecule | CD163 | 7.0 | 3.9 | 5.3 |
| 215602_at | FYVE, RhoGEF and PH domain containing 2 | FGD2 | 6.3 | 4.5 | 5.3 |
| 230391_at | CD84 molecule | CD84 | 6.5 | 4.3 | 5.3 |
| 201105_at | lectin, galactoside-binding, soluble, 1 | LGALS1 | 5.9 | 4.6 | 5.2 |
| 203186_s_at | S100 calcium binding protein A4 | S100A4 | 7.3 | 3.7 | 5.2 |
| 203561_at | Fc fragment of IgG, low affinity IIa, receptor (CD32) | FCGR2A | 6.9 | 3.9 | 5.2 |
| 204184_s_at | adrenergic, beta, receptor kinase 2 | ADRBK2 | 5.9 | 4.6 | 5.2 |
| 204198_s_at | runt-related transcription factor 3 | RUNX3 | 5.3 | 5.1 | 5.2 |
| 206974_at | chemokine (C-X-C motif) receptor 6 | CXCR6 | 4.7 | 5.8 | 5.2 |
| 208949_s_at | lectin, galactoside-binding, soluble, 3 | LGALS3 | 5.9 | 4.6 | 5.2 |
| 209555_s_at | CD36 molecule (thrombospondin receptor) | CD36 | 5.6 | 4.8 | 5.2 |
| 209901_x_at | allograft inflammatory factor 1 | AIF1 | 7.0 | 4.0 | 5.2 |
| 213539_at | CD3d molecule, delta (CD3-TCR complex) | CD3D | 5.6 | 4.8 | 5.2 |
| 214181_x_at | leukocyte specific transcript 1 | LST1 | 6.3 | 4.3 | 5.2 |
| 216920_s_at | TCR gamma alternate reading frame protein  T cell receptor gamma constant 2 | TARP  TRGC2 | 6.3 | 4.2 | 5.2 |
| 217983_s_at | ribonuclease T2 | RNASET2 | 5.9 | 4.6 | 5.2 |
| 219506_at | chromosome 1 open reading frame 54 | C1orf54 | 6.7 | 4.0 | 5.2 |
| 226219_at | Rho GTPase activating protein 30 | ARHGAP30 | 5.9 | 4.5 | 5.2 |
| 228376_at | glycoprotein, alpha-galactosyltransferase 1 pseudogene | GGTA1 | 6.5 | 4.2 | 5.2 |
| 228964_at | PR domain containing 1, with ZNF domain | PRDM1 | 5.3 | 5.0 | 5.2 |
| 230836_at | ST8 alpha-N-acetyl-neuraminide alpha-2,8-sialyltransferase 4 | ST8SIA4 | 6.1 | 4.4 | 5.2 |
| 49306_at | Ras association (RalGDS/AF-6) domain family member 4 | RASSF4 | 6.9 | 3.9 | 5.2 |
| 1552553_a_at | NLR family, CARD domain containing 4 | NLRC4 | 6.6 | 3.9 | 5.1 |
| 201792_at | AE binding protein 1 | AEBP1 | 7.5 | 3.5 | 5.1 |
| 202803_s_at | integrin, beta 2 (complement component 3 receptor 3 and 4 subunit) | ITGB2 | 6.1 | 4.2 | 5.1 |
| 205547_s_at | transgelin | TAGLN | 5.4 | 4.7 | 5.1 |
| 209685_s_at | protein kinase C, beta | PRKCB | 6.0 | 4.3 | 5.1 |
| 224356_x_at | membrane-spanning 4-domains, subfamily A, member 6A | MS4A6A | 6.4 | 4.0 | 5.1 |
| 225846_at | epithelial splicing regulatory protein 1 | ESRP1 | 8.8 | 3.0 | 5.1 |
| 226017_at | CKLF-like MARVEL transmembrane domain containing 7 | CMTM7 | 6.0 | 4.3 | 5.1 |
| 227645_at | phosphoinositide-3-kinase, regulatory subunit 5 | PIK3R5 | 5.5 | 4.7 | 5.1 |
| 235421_at | Mitogen-activated protein kinase kinase kinase 8 | MAP3K8 | 5.7 | 4.5 | 5.1 |
| 202083_s_at | SEC14-like 1 (S. cerevisiae) | SEC14L1 | 5.6 | 4.4 | 5.0 |
| 203729_at | epithelial membrane protein 3 | EMP3 | 5.6 | 4.5 | 5.0 |
| 207734_at | lymphocyte transmembrane adaptor 1 | LAX1 | 7.0 | 3.6 | 5.0 |
| 212681_at | erythrocyte membrane protein band 4.1-like 3 | EPB41L3 | 5.8 | 4.3 | 5.0 |
| 214723_x_at | ankyrin repeat domain 36 | ANKRD36 | 6.4 | 3.9 | 5.0 |
| 219010_at | chromosome 1 open reading frame 106 | C1orf106 | 5.9 | 4.2 | 5.0 |
| 223592_s_at | ring finger protein 135 | RNF135 | 7.5 | 3.3 | 5.0 |
| 227353_at | transmembrane channel-like 8 | TMC8 | 6.6 | 3.8 | 5.0 |
| 228986_at | oxysterol binding protein-like 8 | OSBPL8 | 5.7 | 4.4 | 5.0 |
| 229228_at | cAMP responsive element binding protein 5 | CREB5 | 4.7 | 5.4 | 5.0 |
| 229686_at | purinergic receptor P2Y, G-protein coupled, 8 | P2RY8 | 6.5 | 3.8 | 5.0 |
| 201288_at | Rho GDP dissociation inhibitor (GDI) beta | ARHGDIB | 5.8 | 4.1 | 4.9 |
| 202998_s_at | lysyl oxidase-like 2 | LOXL2 | 4.3 | 5.5 | 4.9 |
| 203300_x_at | adaptor-related protein complex 1, sigma 2 subunit | AP1S2 | 6.2 | 3.8 | 4.9 |
| 204352_at | TNF receptor-associated factor 5 | TRAF5 | 5.8 | 4.1 | 4.9 |
| 209101_at | connective tissue growth factor | CTGF | 3.8 | 6.4 | 4.9 |
| 209670_at | T cell receptor alpha constant | TRAC | 5.0 | 4.8 | 4.9 |
| 210059_s_at | mitogen-activated protein kinase 13 | MAPK13 | 4.1 | 5.7 | 4.9 |
| 210427_x_at | annexin A2 | ANXA2 | 4.7 | 5.0 | 4.9 |
| 211597_s_at | HOP homeobox | HOPX | 5.8 | 4.2 | 4.9 |
| 212190_at | serpin peptidase inhibitor, clade E (nexin, plasminogen activator inhibitor type 1), member 2 | SERPINE2 | 5.4 | 4.4 | 4.9 |
| 213603_s_at | ras-related C3 botulinum toxin substrate 2 (rho family, small GTP binding protein Rac2) | RAC2 | 5.6 | 4.3 | 4.9 |
| 219478_at | WAP four-disulfide core domain 1 | WFDC1 | 5.7 | 4.2 | 4.9 |
| 224374_s_at | elastin microfibril interfacer 2 | EMILIN2 | 5.4 | 4.4 | 4.9 |
| 225496_s_at | synaptotagmin-like 2 | SYTL2 | 5.7 | 4.2 | 4.9 |
| 226051_at | selenoprotein M | SELM | 7.1 | 3.4 | 4.9 |
| 227014_at | aspartate beta-hydroxylase domain containing 2 | ASPHD2 | 6.3 | 3.8 | 4.9 |
| 1552701_a_at | caspase recruitment domain family, member 16 | CARD16 | 6.1 | 3.8 | 4.8 |
| 1567107_s_at | tropomyosin 4 | TPM4 | 5.4 | 4.4 | 4.8 |
| 200951_s_at | cyclin D2 | CCND2 | 6.3 | 3.7 | 4.8 |
| 203963_at | carbonic anhydrase XII | CA12 | 1.9 | 12.1 | 4.8 |
| 204404_at | solute carrier family 12 (sodium/potassium/chloride transporters), member 2 | SLC12A2 | 8.1 | 2.8 | 4.8 |
| 204639_at | adenosine deaminase | ADA | 5.4 | 4.4 | 4.8 |
| 205499_at | sushi-repeat-containing protein, X-linked 2 | SRPX2 | 5.7 | 4.0 | 4.8 |
| 206666_at | granzyme K (granzyme 3; tryptase II) | GZMK | 5.1 | 4.4 | 4.8 |
| 207224_s_at | sialic acid binding Ig-like lectin 7 | SIGLEC7 | 6.9 | 3.4 | 4.8 |
| 209156_s_at | collagen, type VI, alpha 2 | COL6A2 | 6.5 | 3.6 | 4.8 |
| 218856_at | tumor necrosis factor receptor superfamily, member 21 | TNFRSF21 | 5.5 | 4.2 | 4.8 |
| 221530_s_at | basic helix-loop-helix family, member e41 | BHLHE41 | 6.8 | 3.4 | 4.8 |
| 227677_at | Janus kinase 3 | JAK3 | 5.2 | 4.5 | 4.8 |
| 227811_at | FYVE, RhoGEF and PH domain containing 3 | FGD3 | 5.9 | 4.0 | 4.8 |
| 236656_s_at | hypothetical LOC100288911 | LOC100288911 | 6.8 | 3.4 | 4.8 |
| 238462_at | ubiquitin associated and SH3 domain containing B | UBASH3B | 5.6 | 4.2 | 4.8 |
| 241525_at | hypothetical LOC200772 | LOC200772 | 5.4 | 4.3 | 4.8 |
| 242946_at | --- | --- | 5.1 | 4.5 | 4.8 |
| 244780_at | sphingosine-1-phosphate phosphatase 2 | SGPP2 | 6.5 | 3.6 | 4.8 |
| 40420_at | serine/threonine kinase 10 | STK10 | 5.7 | 4.0 | 4.8 |
| 1552807_a_at | sialic acid binding Ig-like lectin 10 | SIGLEC10 | 7.7 | 2.8 | 4.7 |
| 204490_s_at | CD44 molecule (Indian blood group) | CD44 | 5.6 | 4.0 | 4.7 |
| 204961_s_at | neutrophil cytosolic factor 1  neutrophil cytosolic factor 1B pseudogene  neutrophil cytosolic factor 1C pseudogene | NCF1  NCF1B  NCF1C | 5.2 | 4.2 | 4.7 |
| 206111_at | ribonuclease, RNase A family, 2 (liver, eosinophil-derived neurotoxin) | RNASE2 | 6.6 | 3.3 | 4.7 |
| 209921_at | solute carrier family 7, (cationic amino acid transporter, y+ system) member 11 | SLC7A11 | 3.0 | 7.3 | 4.7 |
| 215440_s_at | brain expressed, X-linked 4 | BEX4 | 6.2 | 3.6 | 4.7 |
| 216222_s_at | myosin X | MYO10 | 6.4 | 3.4 | 4.7 |
| 223502_s_at | tumor necrosis factor (ligand) superfamily, member 13b | TNFSF13B | 5.5 | 4.1 | 4.7 |
| 225285_at | branched chain amino-acid transaminase 1, cytosolic | BCAT1 | 4.2 | 5.3 | 4.7 |
| 229390_at | family with sequence similarity 26, member F | FAM26F | 5.8 | 3.8 | 4.7 |
| 229510_at | membrane-spanning 4-domains, subfamily A, member 14 | MS4A14 | 5.2 | 4.2 | 4.7 |
| 234987_at | SAM domain and HD domain 1 | SAMHD1 | 5.5 | 4.0 | 4.7 |
| 235291_s_at | hypothetical LOC643977 | FLJ32255 | 5.4 | 4.0 | 4.7 |
| 240413_at | pyrin and HIN domain family, member 1 | PYHIN1 | 4.9 | 4.6 | 4.7 |
| 201422_at | interferon, gamma-inducible protein 30 | IFI30 | 5.0 | 4.2 | 4.6 |
| 201917_s_at | solute carrier family 25, member 36 | SLC25A36 | 6.4 | 3.3 | 4.6 |
| 202935_s_at | SRY (sex determining region Y)-box 9 | SOX9 | 7.2 | 2.9 | 4.6 |
| 206210_s_at | cholesteryl ester transfer protein, plasma | CETP | 5.0 | 4.2 | 4.6 |
| 211538_s_at | heat shock 70kDa protein 2 | HSPA2 | 5.0 | 4.2 | 4.6 |
| 211709_s_at | C-type lectin domain family 11, member A | CLEC11A | 6.6 | 3.3 | 4.6 |
| 213241_at | plexin C1 | PLXNC1 | 5.9 | 3.5 | 4.6 |
| 214084_x_at | neutrophil cytosolic factor 1C pseudogene | NCF1C | 5.4 | 3.9 | 4.6 |
| 222895_s_at | B-cell CLL/lymphoma 11B (zinc finger protein) | BCL11B | 5.2 | 4.1 | 4.6 |
| 242871_at | progestin and adipoQ receptor family member V | PAQR5 | 4.5 | 4.6 | 4.6 |
| 202464_s_at | 6-phosphofructo-2-kinase/fructose-2,6-biphosphatase 3 | PFKFB3 | 4.9 | 4.0 | 4.5 |
| 202503_s_at | KIAA0101 | KIAA0101 | 3.0 | 6.8 | 4.5 |
| 202786_at | serine threonine kinase 39 | STK39 | 5.4 | 3.7 | 4.5 |
| 203650_at | protein C receptor, endothelial | PROCR | 8.7 | 2.3 | 4.5 |
| 204446_s_at | arachidonate 5-lipoxygenase | ALOX5 | 6.8 | 3.0 | 4.5 |
| 205098_at | chemokine (C-C motif) receptor 1 | CCR1 | 5.9 | 3.4 | 4.5 |
| 205237_at | ficolin (collagen/fibrinogen domain containing) 1 | FCN1 | 4.6 | 4.4 | 4.5 |
| 207979_s_at | CD8b molecule | CD8B | 3.9 | 5.1 | 4.5 |
| 208982_at | platelet/endothelial cell adhesion molecule | PECAM1 | 6.3 | 3.2 | 4.5 |
| 213905_x_at | biglycan | BGN | 5.9 | 3.5 | 4.5 |
| 217867_x_at | beta-site APP-cleaving enzyme 2 | BACE2 | 6.2 | 3.2 | 4.5 |
| 225502_at | dedicator of cytokinesis 8 | DOCK8 | 5.2 | 3.8 | 4.5 |
| 227006_at | protein phosphatase 1, regulatory (inhibitor) subunit 14A | PPP1R14A | 6.1 | 3.4 | 4.5 |
| 228094_at | adhesion molecule, interacts with CXADR antigen 1 | AMICA1 | 5.4 | 3.7 | 4.5 |
| 232231_at | runt-related transcription factor 2 | RUNX2 | 5.0 | 4.0 | 4.5 |
| 238581_at | guanylate binding protein 5 | GBP5 | 5.1 | 4.0 | 4.5 |
| 1557080_s_at | integrin, beta-like 1 (with EGF-like repeat domains) | ITGBL1 | 7.3 | 2.7 | 4.4 |
| 204006_s_at | Fc fragment of IgG, low affinity IIIa, receptor (CD16a)  Fc fragment of IgG, low affinity IIIb, receptor (CD16b) | FCGR3A FCGR3B | 4.8 | 4.0 | 4.4 |
| 204083_s_at | tropomyosin 2 (beta) | TPM2 | 5.9 | 3.3 | 4.4 |
| 204430_s_at | solute carrier family 2 (facilitated glucose/fructose transporter), member 5 | SLC2A5 | 4.0 | 4.9 | 4.4 |
| 206150_at | CD27 molecule | CD27 | 5.2 | 3.7 | 4.4 |
| 206991_s_at | chemokine (C-C motif) receptor 5 | CCR5 | 4.3 | 4.6 | 4.4 |
| 208396_s_at | phosphodiesterase 1A, calmodulin-dependent | PDE1A | 7.1 | 2.7 | 4.4 |
| 209047_at | aquaporin 1 (Colton blood group) | AQP1 | 7.1 | 2.7 | 4.4 |
| 214973_x_at | immunoglobulin heavy constant delta | IGHD | 6.3 | 3.1 | 4.4 |
| 221269_s_at | SH3 domain binding glutamic acid-rich protein like 3 | SH3BGRL3 | 4.4 | 4.3 | 4.4 |
| 226142_at | GLI pathogenesis-related 1 | GLIPR1 | 5.4 | 3.7 | 4.4 |
| 226930_at | fibronectin type III domain containing 1 | FNDC1 | 7.6 | 2.6 | 4.4 |
| 228869_at | sorting nexin 20 | SNX20 | 5.6 | 3.4 | 4.4 |
| 244033_at | chromosome 14 open reading frame 145 | C14orf145 | 5.0 | 3.9 | 4.4 |
| 1553043_a_at | CD300 molecule-like family member f | CD300LF | 5.0 | 3.7 | 4.3 |
| 200701_at | Niemann-Pick disease, type C2 | NPC2 | 5.2 | 3.5 | 4.3 |
| 200824_at | glutathione S-transferase pi 1 | GSTP1 | 6.0 | 3.1 | 4.3 |
| 201426_s_at | vimentin | VIM | 5.4 | 3.4 | 4.3 |
| 203362_s_at | MAD2 mitotic arrest deficient-like 1 (yeast) | MAD2L1 | 2.7 | 6.8 | 4.3 |
| 204220_at | glia maturation factor, gamma | GMFG | 5.7 | 3.3 | 4.3 |
| 205328_at | claudin 10 | CLDN10 | 8.4 | 2.2 | 4.3 |
| 205901_at | prepronociceptin | PNOC | 6.1 | 3.1 | 4.3 |
| 206804_at | CD3g molecule, gamma (CD3-TCR complex) | CD3G | 4.8 | 3.8 | 4.3 |
| 210889_s_at | Fc fragment of IgG, low affinity IIb, receptor (CD32) | FCGR2B | 7.0 | 2.6 | 4.3 |
| 211742_s_at | ecotropic viral integration site 2B | EVI2B | 4.8 | 3.8 | 4.3 |
| 212022_s_at | antigen identified by monoclonal antibody Ki-67 | MKI67 | 3.0 | 6.2 | 4.3 |
| 219403_s_at | heparanase | HPSE | 4.8 | 3.8 | 4.3 |
| 221666_s_at | PYD and CARD domain containing | PYCARD | 5.4 | 3.4 | 4.3 |
| 225175_s_at | solute carrier family 44, member 2 | SLC44A2 | 4.9 | 3.9 | 4.3 |
| 226363_at | ATP-binding cassette, sub-family C (CFTR/MRP), member 5 | ABCC5 | 5.1 | 3.7 | 4.3 |
| 228608_at | sodium leak channel, non-selective | NALCN | 6.3 | 2.9 | 4.3 |
| 238356_at | dedicator of cytokinesis 11 | DOCK11 | 5.3 | 3.4 | 4.3 |
| 204480_s_at | chromosome 9 open reading frame 16 | C9orf16 | 4.3 | 4.2 | 4.2 |
| 205639_at | acyloxyacyl hydrolase (neutrophil) | AOAH | 5.0 | 3.6 | 4.2 |
| 205668_at | lymphocyte antigen 75 | LY75 | 5.6 | 3.2 | 4.2 |
| 207677_s_at | neutrophil cytosolic factor 4, 40kDa | NCF4 | 5.9 | 3.0 | 4.2 |
| 207777_s_at | SP140 nuclear body protein | SP140 | 4.8 | 3.6 | 4.2 |
| 207857_at | leukocyte immunoglobulin-like receptor, subfamily A (with TM domain), member 2 | LILRA2 | 5.7 | 3.1 | 4.2 |
| 208816_x_at | annexin A2 pseudogene 2 | ANXA2P2 | 4.0 | 4.3 | 4.2 |
| 209360_s_at | runt-related transcription factor 1 | RUNX1 | 4.9 | 3.6 | 4.2 |
| 212737_at | GM2 ganglioside activator | GM2A | 4.5 | 3.8 | 4.2 |
| 212873_at | histocompatibility (minor) HA-1 | HMHA1 | 5.2 | 3.4 | 4.2 |
| 213446_s_at | IQ motif containing GTPase activating protein 1 | IQGAP1 | 5.3 | 3.3 | 4.2 |
| 213703_at | hypothetical LOC150759 | LOC150759 | 4.6 | 3.9 | 4.2 |
| 218084_x_at | FXYD domain containing ion transport regulator 5 | FXYD5 | 4.9 | 3.6 | 4.2 |
| 218662_s_at | non-SMC condensin I complex, subunit G | NCAPG | 2.7 | 6.4 | 4.2 |
| 220446_s_at | carbohydrate (N-acetylglucosamine 6-O) sulfotransferase 4 | CHST4 | 6.4 | 2.7 | 4.2 |
| 222281_s_at | hypothetical LOC100505650 | LOC100505650 | 5.8 | 3.0 | 4.2 |
| 223551_at | protein kinase (cAMP-dependent, catalytic) inhibitor beta | PKIB | 2.5 | 7.1 | 4.2 |
| 232068_s_at | toll-like receptor 4 | TLR4 | 5.5 | 3.3 | 4.2 |
| 238949_at | ring finger protein 145 | RNF145 | 4.9 | 3.6 | 4.2 |
| 31845_at | E74-like factor 4 (ets domain transcription factor) | ELF4 | 5.0 | 3.6 | 4.2 |
| 202804_at | ATP-binding cassette, sub-family C (CFTR/MRP), member 1 | ABCC1 | 4.6 | 3.7 | 4.1 |
| 202910_s_at | CD97 molecule | CD97 | 4.4 | 3.8 | 4.1 |
| 203903_s_at | hephaestin | HEPH | 6.7 | 2.5 | 4.1 |
| 204007_at | Fc fragment of IgG, low affinity IIIb, receptor (CD16b) | FCGR3B | 4.6 | 3.6 | 4.1 |
| 204118_at | CD48 molecule | CD48 | 4.8 | 3.5 | 4.1 |
| 204137_at | G protein-coupled receptor 137B | GPR137B | 5.1 | 3.2 | 4.1 |
| 208438_s_at | Gardner-Rasheed feline sarcoma viral (v-fgr) oncogene homolog | FGR | 4.3 | 3.9 | 4.1 |
| 208850_s_at | Thy-1 cell surface antigen | THY1 | 5.8 | 2.9 | 4.1 |
| 209651_at | transforming growth factor beta 1 induced transcript 1 | TGFB1I1 | 4.7 | 3.7 | 4.1 |
| 209708_at | monooxygenase, DBH-like 1 | MOXD1 | 5.8 | 2.9 | 4.1 |
| 210448_s_at | purinergic receptor P2X, ligand-gated ion channel, 5 | P2RX5 | 4.7 | 3.6 | 4.1 |
| 213982_s_at | RAB GTPase activating protein 1-like | RABGAP1L | 5.1 | 3.3 | 4.1 |
| 218585_s_at | denticleless homolog (Drosophila) | DTL | 4.0 | 4.3 | 4.1 |
| 218883_s_at | MLF1 interacting protein | MLF1IP | 2.7 | 6.4 | 4.1 |
| 226525_at | serine/threonine kinase 17b | STK17B | 4.5 | 3.8 | 4.1 |
| 226603_at | sterile alpha motif domain containing 9-like | SAMD9L | 5.4 | 3.1 | 4.1 |
| 228592_at | membrane-spanning 4-domains, subfamily A, member 1 | MS4A1 | 4.9 | 3.4 | 4.1 |
| 229560_at | toll-like receptor 8 | TLR8 | 5.6 | 3.0 | 4.1 |
| 235030_at | family with sequence similarity 55, member C | FAM55C | 5.0 | 3.4 | 4.1 |
| 235085_at | homolog of rat pragma of Rnd2 | SGK223 | 4.1 | 4.1 | 4.1 |
| 235593_at | zinc finger E-box binding homeobox 2 | ZEB2 | 5.6 | 3.0 | 4.1 |
| 236341_at | cytotoxic T-lymphocyte-associated protein 4 | CTLA4 | 3.3 | 5.1 | 4.1 |
| 236429_at | zinc finger protein 83 | ZNF83 | 5.1 | 3.3 | 4.1 |
| 1554240_a_at | integrin, alpha L (antigen CD11A (p180), lymphocyte function-associated antigen 1; alpha polypeptide) | ITGAL | 4.4 | 3.6 | 4.0 |
| 201438_at | collagen, type VI, alpha 3 | COL6A3 | 5.6 | 2.9 | 4.0 |
| 203167_at | TIMP metallopeptidase inhibitor 2 | TIMP2 | 5.2 | 3.1 | 4.0 |
| 203554_x_at | pituitary tumor-transforming 1 | PTTG1 | 2.9 | 5.5 | 4.0 |
| 204014_at | dual specificity phosphatase 4 | DUSP4 | 4.6 | 3.4 | 4.0 |
| 204638_at | acid phosphatase 5, tartrate resistant | ACP5 | 4.9 | 3.3 | 4.0 |
| 204834_at | fibrinogen-like 2 | FGL2 | 5.5 | 2.9 | 4.0 |
| 205466_s_at | heparan sulfate (glucosamine) 3-O-sulfotransferase 1 | HS3ST1 | 5.6 | 2.9 | 4.0 |
| 206513_at | absent in melanoma 2 | AIM2 | 4.6 | 3.4 | 4.0 |
| 209457_at | dual specificity phosphatase 5 | DUSP5 | 3.3 | 4.8 | 4.0 |
| 209682_at | Cas-Br-M (murine) ecotropic retroviral transforming sequence b | CBLB | 4.2 | 3.8 | 4.0 |
| 211434_s_at | chemokine (C-C motif) receptor-like 2 | CCRL2 | 4.3 | 3.8 | 4.0 |
| 217529_at | ORAI calcium release-activated calcium modulator 2 | ORAI2 | 5.6 | 2.8 | 4.0 |
| 219279_at | dedicator of cytokinesis 10 | DOCK10 | 5.1 | 3.1 | 4.0 |
| 219358_s_at | ArfGAP with dual PH domains 2 | ADAP2 | 6.0 | 2.7 | 4.0 |
| 219387_at | coiled-coil domain containing 88A | CCDC88A | 4.4 | 3.7 | 4.0 |
| 220005_at | purinergic receptor P2Y, G-protein coupled, 13 | P2RY13 | 6.0 | 2.7 | 4.0 |
| 222877_at | --- | --- | 5.7 | 2.8 | 4.0 |
| 225706_at | glucocorticoid induced transcript 1 | GLCCI1 | 5.0 | 3.3 | 4.0 |
| 227091_at | coiled-coil domain containing 146 | CCDC146 | 4.6 | 3.5 | 4.0 |
| 227194_at | family with sequence similarity 3, member B | FAM3B | 6.8 | 2.3 | 4.0 |
| 227276_at | plexin domain containing 2 | PLXDC2 | 5.5 | 2.9 | 4.0 |
| 228821_at | ST6 beta-galactosamide alpha-2,6-sialyltranferase 2 | ST6GAL2 | 7.2 | 2.2 | 4.0 |
| 232315_at | zinc finger protein 880 | ZNF880 | 5.5 | 2.9 | 4.0 |
| 236782_at | sterile alpha motif domain containing 3 | SAMD3 | 4.3 | 3.8 | 4.0 |
| 237466_s_at | hedgehog interacting protein | HHIP | 6.2 | 2.5 | 4.0 |
| 238013_at | pleckstrin homology domain containing, family A (phosphoinositide binding specific) member 2 | PLEKHA2 | 4.6 | 3.4 | 4.0 |
| 1552398_a_at | C-type lectin domain family 12, member A | CLEC12A | 4.4 | 3.4 | 3.9 |
| 202705_at | cyclin B2 | CCNB2 | 2.5 | 6.3 | 3.9 |
| 203332_s_at | inositol polyphosphate-5-phosphatase, 145kDa | INPP5D | 4.7 | 3.2 | 3.9 |
| 203528_at | sema domain, immunoglobulin domain (Ig), transmembrane domain (TM) and short cytoplasmic domain, (semaphorin) 4D | SEMA4D | 4.7 | 3.2 | 3.9 |
| 203665_at | heme oxygenase (decycling) 1 | HMOX1 | 4.6 | 3.4 | 3.9 |
| 204272_at | lectin, galactoside-binding, soluble, 4 | LGALS4 | 3.5 | 4.4 | 3.9 |
| 204882_at | Rho GTPase activating protein 25 | ARHGAP25 | 4.9 | 3.1 | 3.9 |
| 208268_at | ADAM metallopeptidase domain 28 | ADAM28 | 5.4 | 2.8 | 3.9 |
| 209714_s_at | cyclin-dependent kinase inhibitor 3 | CDKN3 | 2.1 | 7.3 | 3.9 |
| 210644_s_at | leukocyte-associated immunoglobulin-like receptor 1 | LAIR1 | 4.2 | 3.7 | 3.9 |
| 213275_x_at | cathepsin B | CTSB | 4.4 | 3.5 | 3.9 |
| 213906_at | v-myb myeloblastosis viral oncogene homolog (avian)-like 1 | MYBL1 | 4.1 | 3.8 | 3.9 |
| 215241_at | anoctamin 3 | ANO3 | 4.9 | 3.1 | 3.9 |
| 218051_s_at | 5'-nucleotidase domain containing 2 | NT5DC2 | 4.8 | 3.2 | 3.9 |
| 218186_at | RAB25, member RAS oncogene family | RAB25 | 6.1 | 2.6 | 3.9 |
| 218324_s_at | spermatogenesis associated, serine-rich 2 | SPATS2 | 4.9 | 3.1 | 3.9 |
| 220577_at | GTPase, very large interferon inducible pseudogene 1 | GVINP1 | 4.5 | 3.4 | 3.9 |
| 222218_s_at | paired immunoglobin-like type 2 receptor alpha | PILRA | 4.2 | 3.6 | 3.9 |
| 224901_at | stearoyl-CoA desaturase 5 | SCD5 | 6.3 | 2.4 | 3.9 |
| 225720_at | synaptopodin 2 | SYNPO2 | 4.4 | 3.5 | 3.9 |
| 226279_at | protease, serine, 23 | PRSS23 | 6.2 | 2.5 | 3.9 |
| 226372_at | carbohydrate (chondroitin 4) sulfotransferase 11 | CHST11 | 4.2 | 3.5 | 3.9 |
| 226811_at | family with sequence similarity 46, member C | FAM46C | 5.1 | 3.1 | 3.9 |
| 227184_at | platelet-activating factor receptor | PTAFR | 4.2 | 3.5 | 3.9 |
| 228071_at | GTPase, IMAP family member 7 | GIMAP7 | 4.7 | 3.2 | 3.9 |
| 235765_at | transducin-like enhancer of split 4 (E(sp1) homolog, Drosophila) | TLE4 | 5.3 | 2.9 | 3.9 |
| 240304_s_at | transmembrane channel-like 5 | TMC5 | 5.9 | 2.6 | 3.9 |
| 200736_s_at | glutathione peroxidase 1 | GPX1 | 4.0 | 3.6 | 3.8 |
| 200923_at | lectin, galactoside-binding, soluble, 3 binding protein | LGALS3BP | 4.1 | 3.4 | 3.8 |
| 203741_s_at | adenylate cyclase 7 | ADCY7 | 4.7 | 3.2 | 3.8 |
| 203879_at | phosphoinositide-3-kinase, catalytic, delta polypeptide | PIK3CD | 5.0 | 2.8 | 3.8 |
| 204269_at | pim-2 oncogene | PIM2 | 4.2 | 3.4 | 3.8 |
| 204527_at | myosin VA (heavy chain 12, myoxin) | MYO5A | 4.7 | 3.0 | 3.8 |
| 204923_at | SAM and SH3 domain containing 3 | SASH3 | 4.6 | 3.1 | 3.8 |
| 205081_at | cysteine-rich protein 1 (intestinal) | CRIP1 | 4.3 | 3.3 | 3.8 |
| 208837_at | transmembrane emp24 protein transport domain containing 3 | TMED3 | 4.2 | 3.5 | 3.8 |
| 209529_at | phosphatidic acid phosphatase type 2C | PPAP2C | 5.3 | 2.7 | 3.8 |
| 209909_s_at | transforming growth factor, beta 2 | TGFB2 | 3.9 | 3.6 | 3.8 |
| 213593_s_at | transformer 2 alpha homolog (Drosophila) | TRA2A | 4.8 | 3.1 | 3.8 |
| 215894_at | prostaglandin D2 receptor (DP) | PTGDR | 5.5 | 2.6 | 3.8 |
| 224710_at | RAB34, member RAS oncogene family | RAB34 | 5.1 | 2.9 | 3.8 |
| 225955_at | meteorin, glial cell differentiation regulator-like | METRNL | 3.8 | 3.8 | 3.8 |
| 242352_at | Nipped-B homolog (Drosophila) | NIPBL | 4.7 | 3.1 | 3.8 |
| 242560_at | Fanconi anemia, complementation group D2 | FANCD2 | 2.8 | 5.0 | 3.8 |
| 1558972_s_at | thymocyte selection associated | THEMIS | 3.8 | 3.5 | 3.7 |
| 200975_at | palmitoyl-protein thioesterase 1 | PPT1 | 4.3 | 3.2 | 3.7 |
| 201058_s_at | myosin, light chain 9, regulatory | MYL9 | 4.8 | 2.8 | 3.7 |
| 201818_at | lysophosphatidylcholine acyltransferase 1 | LPCAT1 | 3.9 | 3.5 | 3.7 |
| 202766_s_at | fibrillin 1 | FBN1 | 4.8 | 2.8 | 3.7 |
| 203753_at | transcription factor 4 | TCF4 | 4.5 | 3.0 | 3.7 |
| 204682_at | latent transforming growth factor beta binding protein 2 | LTBP2 | 5.1 | 2.7 | 3.7 |
| 207828_s_at | centromere protein F, 350/400kDa (mitosin) | CENPF | 2.4 | 5.6 | 3.7 |
| 211339_s_at | IL2-inducible T-cell kinase | ITK | 3.7 | 3.6 | 3.7 |
| 213564_x_at | lactate dehydrogenase B | LDHB | 4.0 | 3.5 | 3.7 |
| 216237_s_at | minichromosome maintenance complex component 5 | MCM5 | 3.3 | 4.1 | 3.7 |
| 221698_s_at | C-type lectin domain family 7, member A | CLEC7A | 4.1 | 3.3 | 3.7 |
| 221729_at | collagen, type V, alpha 2 | COL5A2 | 4.1 | 3.2 | 3.7 |
| 223303_at | fermitin family member 3 | FERMT3 | 4.8 | 2.9 | 3.7 |
| 225602_at | GLI pathogenesis-related 2 | GLIPR2 | 4.3 | 3.1 | 3.7 |
| 228581_at | potassium inwardly-rectifying channel, subfamily J, member 10 | KCNJ10 | 4.8 | 2.9 | 3.7 |
| 231202_at | aldehyde dehydrogenase 1 family, member L2 | ALDH1L2 | 4.1 | 3.4 | 3.7 |
| 236295_s_at | NLR family, CARD domain containing 3 | NLRC3 | 4.5 | 3.0 | 3.7 |
| 237753_at | interleukin 21 receptor | IL21R | 4.7 | 2.8 | 3.7 |
| 40687_at | gap junction protein, alpha 4, 37kDa | GJA4 | 6.0 | 2.3 | 3.7 |
| 201954_at | actin related protein 2/3 complex, subunit 1B, 41kDa | ARPC1B | 4.0 | 3.3 | 3.6 |
| 202877_s_at | CD93 molecule | CD93 | 4.5 | 2.9 | 3.6 |
| 202965_s_at | calpain 6 | CAPN6 | 5.8 | 2.3 | 3.6 |
| 204750_s_at | desmocollin 2 | DSC2 | 4.1 | 3.1 | 3.6 |
| 205532_s_at | cadherin 6, type 2, K-cadherin (fetal kidney) | CDH6 | 4.7 | 2.8 | 3.6 |
| 206247_at | MHC class I polypeptide-related sequence B | MICB | 3.9 | 3.4 | 3.6 |
| 206296_x_at | mitogen-activated protein kinase kinase kinase kinase 1 | MAP4K1 | 4.2 | 3.1 | 3.6 |
| 206391_at | retinoic acid receptor responder (tazarotene induced) 1 | RARRES1 | 5.3 | 2.4 | 3.6 |
| 206500_s_at | chromosome 14 open reading frame 106 | C14orf106 | 4.3 | 3.1 | 3.6 |
| 207610_s_at | egf-like module containing, mucin-like, hormone receptor-like 2 | EMR2 | 3.7 | 3.6 | 3.6 |
| 210140_at | cystatin F (leukocystatin) | CST7 | 3.6 | 3.6 | 3.6 |
| 216905_s_at | suppression of tumorigenicity 14 (colon carcinoma) | ST14 | 3.7 | 3.4 | 3.6 |
| 218355_at | kinesin family member 4A | KIF4A | 2.2 | 6.0 | 3.6 |
| 219014_at | placenta-specific 8 | PLAC8 | 5.0 | 2.6 | 3.6 |
| 221538_s_at | plexin A1 | PLXNA1 | 3.7 | 3.4 | 3.6 |
| 223640_at | hematopoietic cell signal transducer | HCST | 4.0 | 3.2 | 3.6 |
| 226535_at | integrin, beta 6 | ITGB6 | 3.9 | 3.4 | 3.6 |
| 227862_at | TMF1-regulated nuclear protein 1 | TRNP1 | 2.9 | 4.5 | 3.6 |
| 227863_at | CD225 family protein FLJ76511 | LOC402778 | 4.9 | 2.6 | 3.6 |
| 229715_at | --- | --- | 2.4 | 5.5 | 3.6 |
| 235457_at | mastermind-like 2 (Drosophila) | MAML2 | 4.8 | 2.7 | 3.6 |
| 37408_at | mannose receptor, C type 2 | MRC2 | 4.5 | 2.8 | 3.6 |
| 1558569_at | Hypothetical LOC100131541 | LOC100131541 | 4.6 | 2.7 | 3.5 |
| 1568964_x_at | sialophorin | SPN | 3.8 | 3.2 | 3.5 |
| 201294_s_at | WD repeat and SOCS box-containing 1 | WSB1 | 4.1 | 3.1 | 3.5 |
| 201505_at | laminin, beta 1 | LAMB1 | 4.5 | 2.7 | 3.5 |
| 202267_at | laminin, gamma 2 | LAMC2 | 4.2 | 2.9 | 3.5 |
| 202806_at | drebrin 1 | DBN1 | 4.2 | 3.0 | 3.5 |
| 203819_s_at | insulin-like growth factor 2 mRNA binding protein 3 | IGF2BP3 | 3.3 | 3.7 | 3.5 |
| 204061_at | protein kinase, X-linked | PRKX | 4.1 | 3.0 | 3.5 |
| 206715_at | transcription factor EC | TFEC | 4.8 | 2.6 | 3.5 |
| 206723_s_at | lysophosphatidic acid receptor 2 | LPAR2 | 4.1 | 3.0 | 3.5 |
| 209211_at | Kruppel-like factor 5 (intestinal) | KLF5 | 4.2 | 2.9 | 3.5 |
| 209568_s_at | ral guanine nucleotide dissociation stimulator-like 1 | RGL1 | 4.6 | 2.6 | 3.5 |
| 209846_s_at | butyrophilin, subfamily 3, member A2 | BTN3A2 | 4.0 | 3.0 | 3.5 |
| 210220_at | frizzled homolog 2 (Drosophila) | FZD2 | 4.6 | 2.6 | 3.5 |
| 211911_x_at | major histocompatibility complex, class I, B | HLA-B | 3.7 | 3.4 | 3.5 |
| 213007_at | Fanconi anemia, complementation group I | FANCI | 2.9 | 4.2 | 3.5 |
| 213620_s_at | intercellular adhesion molecule 2 | ICAM2 | 4.6 | 2.7 | 3.5 |
| 214470_at | killer cell lectin-like receptor subfamily B, member 1 | KLRB1 | 4.1 | 3.0 | 3.5 |
| 214735_at | interaction protein for cytohesin exchange factors 1 | IPCEF1 | 3.7 | 3.2 | 3.5 |
| 214830_at | solute carrier family 38, member 6 | SLC38A6 | 3.4 | 3.5 | 3.5 |
| 217138_x_at | partial mRNA for human Ig lambda light chain variable region, clone MB91 (331 bp)* | IGLV | 3.9 | 3.0 | 3.5 |
| 218217_at | serine carboxypeptidase 1 | SCPEP1 | 4.5 | 2.7 | 3.5 |
| 218796_at | fermitin family member 1 | FERMT1 | 3.6 | 3.4 | 3.5 |
| 218802_at | coiled-coil domain containing 109B | CCDC109B | 3.9 | 3.2 | 3.5 |
| 219243_at | GTPase, IMAP family member 4 | GIMAP4 | 4.0 | 3.0 | 3.5 |
| 219799_s_at | dehydrogenase/reductase (SDR family) member 9 | DHRS9 | 3.9 | 3.2 | 3.5 |
| 220177_s_at | transmembrane protease, serine 3 | TMPRSS3 | 5.1 | 2.4 | 3.5 |
| 221765_at | UDP-glucose ceramide glucosyltransferase | UGCG | 3.5 | 3.4 | 3.5 |
| 224694_at | anthrax toxin receptor 1 | ANTXR1 | 4.6 | 2.6 | 3.5 |
| 226828_s_at | hairy/enhancer-of-split related with YRPW motif-like | HEYL | 3.9 | 3.2 | 3.5 |
| 227297_at | integrin, alpha 9 | ITGA9 | 4.8 | 2.6 | 3.5 |
| 228258_at | TBC1 domain family, member 10C | TBC1D10C | 3.9 | 3.2 | 3.5 |
| 228754_at | solute carrier family 6 (neurotransmitter transporter, taurine), member 6 | SLC6A6 | 3.6 | 3.4 | 3.5 |
| 229010_at | Cas-Br-M (murine) ecotropic retroviral transforming sequence | CBL | 3.7 | 3.3 | 3.5 |
| 229242_at | --- | --- | 3.7 | 3.3 | 3.5 |
| 229614_at | zinc finger protein 320 | ZNF320 | 4.8 | 2.5 | 3.5 |
| 236539_at | protein tyrosine phosphatase, non-receptor type 22 (lymphoid) | PTPN22 | 3.6 | 3.3 | 3.5 |
| 1552302_at | transmembrane protein 106A | TMEM106A | 4.4 | 2.6 | 3.4 |
| 1557051_s_at | --- | --- | 3.8 | 3.0 | 3.4 |
| 1557227_s_at | translocated promoter region (to activated MET oncogene) | TPR | 3.8 | 3.0 | 3.4 |
| 200648_s_at | glutamate-ammonia ligase | GLUL | 3.4 | 3.5 | 3.4 |
| 201069_at | matrix metallopeptidase 2 (gelatinase A, 72kDa gelatinase, 72kDa type IV collagenase) | MMP2 | 5.3 | 2.2 | 3.4 |
| 201136_at | proteolipid protein 2 (colonic epithelium-enriched) | PLP2 | 3.2 | 3.7 | 3.4 |
| 201462_at | secernin 1 | SCRN1 | 4.8 | 2.4 | 3.4 |
| 201670_s_at | myristoylated alanine-rich protein kinase C substrate | MARCKS | 3.7 | 3.0 | 3.4 |
| 202746_at | integral membrane protein 2A | ITM2A | 4.4 | 2.5 | 3.4 |
| 203685_at | B-cell CLL/lymphoma 2 | BCL2 | 4.1 | 2.8 | 3.4 |
| 205101_at | class II, major histocompatibility complex, transactivator | CIITA | 3.8 | 3.0 | 3.4 |
| 205213_at | ArfGAP with coiled-coil, ankyrin repeat and PH domains 1 | ACAP1 | 3.8 | 3.1 | 3.4 |
| 205559_s_at | proprotein convertase subtilisin/kexin type 5 | PCSK5 | 3.7 | 3.1 | 3.4 |
| 208079_s_at | aurora kinase A | AURKA | 2.0 | 6.0 | 3.4 |
| 211908_x_at | Immunoglobulin kappa variable 3-20 | IGKV3-20 | 3.7 | 3.0 | 3.4 |
| 212070_at | G protein-coupled receptor 56 | GPR56 | 4.2 | 2.8 | 3.4 |
| 212188_at | potassium channel tetramerisation domain containing 12 | KCTD12 | 4.8 | 2.3 | 3.4 |
| 213438_at | neurofascin | NFASC | 4.9 | 2.4 | 3.4 |
| 213661_at | peptidase domain containing associated with muscle regeneration 1 | PAMR1 | 4.5 | 2.6 | 3.4 |
| 214196_s_at | tripeptidyl peptidase I | TPP1 | 4.2 | 2.8 | 3.4 |
| 214467_at | G protein-coupled receptor 65 | GPR65 | 4.7 | 2.4 | 3.4 |
| 214500_at | H2A histone family, member Y | H2AFY | 3.9 | 2.9 | 3.4 |
| 215199_at | caldesmon 1 | CALD1 | 3.0 | 3.8 | 3.4 |
| 218380_at | hypothetical protein LOC728392  NLR family, pyrin domain containing 1 | LOC728392 NLRP1 | 4.1 | 2.9 | 3.4 |
| 218451_at | CUB domain containing protein 1 | CDCP1 | 4.1 | 2.8 | 3.4 |
| 218870_at | Rho GTPase activating protein 15 | ARHGAP15 | 4.5 | 2.6 | 3.4 |
| 219825_at | cytochrome P450, family 26, subfamily B, polypeptide 1 | CYP26B1 | 4.3 | 2.6 | 3.4 |
| 220085_at | helicase, lymphoid-specific | HELLS | 3.1 | 3.9 | 3.4 |
| 220112_at | ankyrin repeat domain 55 | ANKRD55 | 4.7 | 2.4 | 3.4 |
| 220252_x_at | chromosome X open reading frame 21 | CXorf21 | 4.2 | 2.8 | 3.4 |
| 221978_at | major histocompatibility complex, class I, F | HLA-F | 3.4 | 3.5 | 3.4 |
| 223038_s_at | family with sequence similarity 60, member A | FAM60A | 3.7 | 3.2 | 3.4 |
| 223358_s_at | phosphodiesterase 7A | PDE7A | 4.1 | 2.8 | 3.4 |
| 224909_s_at | phosphatidylinositol-3,4,5-trisphosphate-dependent Rac exchange factor 1 | PREX1 | 3.9 | 3.0 | 3.4 |
| 226084_at | microtubule-associated protein 1B | MAP1B | 4.0 | 2.8 | 3.4 |
| 226659_at | differentially expressed in FDCP 6 homolog (mouse) | DEF6 | 3.8 | 3.0 | 3.4 |
| 227812_at | tumor necrosis factor receptor superfamily, member 19 | TNFRSF19 | 6.4 | 1.9 | 3.4 |
| 228372_at | chromosome 10 open reading frame 128 | C10orf128 | 4.3 | 2.7 | 3.4 |
| 230076_at | PITPNM family member 3 | PITPNM3 | 5.5 | 2.1 | 3.4 |
| 232034_at | Hypothetical protein LOC203274 | LOC203274 | 4.0 | 3.0 | 3.4 |
| 238066_at | retinol binding protein 7, cellular | RBP7 | 4.5 | 2.5 | 3.4 |
| 239237_at | --- | --- | 3.8 | 3.1 | 3.4 |
| 201930_at | minichromosome maintenance complex component 6 | MCM6 | 2.8 | 4.0 | 3.3 |
| 202524_s_at | sparc/osteonectin, cwcv and kazal-like domains proteoglycan (testican) 2 | SPOCK2 | 3.4 | 3.2 | 3.3 |
| 202659_at | proteasome (prosome, macropain) subunit, beta type, 10 | PSMB10 | 3.5 | 3.1 | 3.3 |
| 202719_s_at | testis derived transcript (3 LIM domains) | TES | 3.3 | 3.3 | 3.3 |
| 202800_at | solute carrier family 1 (glial high affinity glutamate transporter), member 3 | SLC1A3 | 3.5 | 3.0 | 3.3 |
| 203508_at | tumor necrosis factor receptor superfamily, member 1B | TNFRSF1B | 3.4 | 3.3 | 3.3 |
| 203921_at | carbohydrate (N-acetylglucosamine-6-O) sulfotransferase 2 | CHST2 | 3.2 | 3.4 | 3.3 |
| 203922_s_at | cytochrome b-245, beta polypeptide | CYBB | 4.3 | 2.5 | 3.3 |
| 204470_at | chemokine (C-X-C motif) ligand 1 (melanoma growth stimulating activity, alpha) | CXCL1 | 4.2 | 2.7 | 3.3 |
| 205117_at | fibroblast growth factor 1 (acidic) | FGF1 | 3.6 | 3.0 | 3.3 |
| 205692_s_at | CD38 molecule | CD38 | 4.7 | 2.3 | 3.3 |
| 205882_x_at | adducin 3 (gamma) | ADD3 | 4.6 | 2.3 | 3.3 |
| 206337_at | chemokine (C-C motif) receptor 7 | CCR7 | 4.2 | 2.5 | 3.3 |
| 207691_x_at | ectonucleoside triphosphate diphosphohydrolase 1 | ENTPD1 | 4.6 | 2.4 | 3.3 |
| 209008_x_at | keratin 8 | KRT8 | 2.3 | 4.8 | 3.3 |
| 209154_at | Tax1 (human T-cell leukemia virus type I) binding protein 3 | TAX1BP3 | 3.7 | 3.1 | 3.3 |
| 209283_at | crystallin, alpha B | CRYAB | 4.0 | 2.7 | 3.3 |
| 210116_at | SH2 domain containing 1A | SH2D1A | 3.7 | 3.1 | 3.3 |
| 212415_at | septin 6 | SEPT6 | 3.2 | 3.3 | 3.3 |
| 213416_at | integrin, alpha 4 (antigen CD49D, alpha 4 subunit of VLA-4 receptor) | ITGA4 | 3.6 | 3.0 | 3.3 |
| 214850_at | glucuronidase, beta pseudogene | LOC100170939 | 4.6 | 2.4 | 3.3 |
| 217362_x_at | major histocompatibility complex, class II, DR beta 6 (pseudogene) | HLA-DRB6 | 3.5 | 3.2 | 3.3 |
| 218501_at | Rho guanine nucleotide exchange factor (GEF) 3 | ARHGEF3 | 4.3 | 2.6 | 3.3 |
| 218755_at | kinesin family member 20A | KIF20A | 2.1 | 5.3 | 3.3 |
| 220088_at | complement component 5a receptor 1 | C5AR1 | 3.0 | 3.8 | 3.3 |
| 221004_s_at | integral membrane protein 2C | ITM2C | 4.0 | 2.7 | 3.3 |
| 222062_at | interleukin 27 receptor, alpha | IL27RA | 3.9 | 2.8 | 3.3 |
| 225530_at | MOB1, Mps One Binder kinase activator-like 2A (yeast) | MOBKL2A | 3.6 | 3.1 | 3.3 |
| 226423_at | progestin and adipoQ receptor family member VIII | PAQR8 | 4.3 | 2.5 | 3.3 |
| 227346_at | IKAROS family zinc finger 1 (Ikaros) | IKZF1 | 4.0 | 2.8 | 3.3 |
| 227850_x_at | CDC42 effector protein (Rho GTPase binding) 5 | CDC42EP5 | 4.9 | 2.2 | 3.3 |
| 228658_at | myocardial infarction associated transcript (non-protein coding) | MIAT | 3.6 | 3.0 | 3.3 |
| 232179_at | Hypothetical protein LOC158863 | LOC158863 | 3.9 | 2.9 | 3.3 |
| 235852_at | Stonin 2 | STON2 | 4.3 | 2.4 | 3.3 |
| 243981_at | serine/threonine kinase 4 | STK4 | 3.5 | 3.0 | 3.3 |
| 244050_at | protein tyrosine phosphatase-like A domain containing 2 | PTPLAD2 | 4.3 | 2.6 | 3.3 |
| 244864_at | hypothetical LOC100505562 | LOC100505562 | 2.3 | 4.5 | 3.3 |
| 1570515_a_at | filamin A interacting protein 1 | FILIP1 | 4.7 | 2.1 | 3.2 |
| 200003_s_at | ribosomal protein L28 | RPL28 | 3.5 | 3.0 | 3.2 |
| 201280_s_at | disabled homolog 2, mitogen-responsive phosphoprotein (Drosophila) | DAB2 | 4.3 | 2.5 | 3.2 |
| 202191_s_at | growth arrest-specific 7 | GAS7 | 4.2 | 2.4 | 3.2 |
| 203423_at | retinol binding protein 1, cellular | RBP1 | 5.2 | 2.0 | 3.2 |
| 203773_x_at | biliverdin reductase A | BLVRA | 3.9 | 2.6 | 3.2 |
| 203915_at | chemokine (C-X-C motif) ligand 9 | CXCL9 | 2.6 | 3.8 | 3.2 |
| 205608_s_at | angiopoietin 1 | ANGPT1 | 4.3 | 2.4 | 3.2 |
| 206082_at | HLA complex P5 | HCP5 | 3.3 | 3.1 | 3.2 |
| 206102_at | GINS complex subunit 1 (Psf1 homolog) | GINS1 | 2.1 | 4.6 | 3.2 |
| 206881_s_at | leukocyte immunoglobulin-like receptor, subfamily A (without TM domain), member 3 | LILRA3 | 3.6 | 2.9 | 3.2 |
| 207277_at | CD209 molecule | CD209 | 4.3 | 2.4 | 3.2 |
| 208659_at | chloride intracellular channel 1 | CLIC1 | 3.3 | 3.1 | 3.2 |
| 209906_at | complement component 3a receptor 1 | C3AR1 | 4.2 | 2.4 | 3.2 |
| 209955_s_at | fibroblast activation protein, alpha | FAP | 4.1 | 2.4 | 3.2 |
| 211947_s_at | HLA-B associated transcript 2-like 2 | BAT2L2 | 3.5 | 2.9 | 3.2 |
| 213415_at | chloride intracellular channel 2 | CLIC2 | 4.2 | 2.5 | 3.2 |
| 218953_s_at | prenylcysteine oxidase 1 like | PCYOX1L | 3.8 | 2.7 | 3.2 |
| 219522_at | four jointed box 1 (Drosophila) | FJX1 | 2.9 | 3.5 | 3.2 |
| 220646_s_at | killer cell lectin-like receptor subfamily F, member 1 | KLRF1 | 3.3 | 3.2 | 3.2 |
| 221974_at | imprinted in Prader-Willi syndrome (non-protein coding) | IPW | 4.6 | 2.2 | 3.2 |
| 222508_s_at | arginine and glutamate rich 1 | ARGLU1 | 3.3 | 3.0 | 3.2 |
| 223307_at | cell division cycle associated 3 | CDCA3 | 2.2 | 4.9 | 3.2 |
| 223499_at | C1q and tumor necrosis factor related protein 5  membrane frizzled-related protein | C1QTNF5 MFRP | 4.1 | 2.4 | 3.2 |
| 224730_at | DDB1 and CUL4 associated factor 7 | DCAF7 | 3.4 | 3.0 | 3.2 |
| 226980_at | DEP domain containing 1B | DEPDC1B | 3.0 | 3.4 | 3.2 |
| 228042_at | ADP-ribosylarginine hydrolase | ADPRH | 3.4 | 2.9 | 3.2 |
| 228176_at | sphingosine-1-phosphate receptor 3 | S1PR3 | 2.9 | 3.6 | 3.2 |
| 230000_at | ring finger protein 213 | RNF213 | 3.6 | 2.8 | 3.2 |
| 235425_at | shugoshin-like 2 (S. pombe) | SGOL2 | 2.9 | 3.5 | 3.2 |
| 235944_at | hemicentin 1 | HMCN1 | 3.6 | 2.8 | 3.2 |
| 238996_x_at | aldolase A, fructose-bisphosphate | ALDOA | 2.8 | 3.7 | 3.2 |
| 1552584_at | interleukin 12 receptor, beta 1 | IL12RB1 | 3.6 | 2.6 | 3.1 |
| 200622_x_at | calmodulin 3 (phosphorylase kinase, delta) | CALM3 | 3.4 | 2.8 | 3.1 |
| 200665_s_at | secreted protein, acidic, cysteine-rich (osteonectin) | SPARC | 3.8 | 2.5 | 3.1 |
| 200907_s_at | palladin, cytoskeletal associated protein | PALLD | 2.9 | 3.2 | 3.1 |
| 201251_at | pyruvate kinase, muscle | PKM2 | 3.3 | 2.9 | 3.1 |
| 201411_s_at | pleckstrin homology domain containing, family B (evectins) member 2 | PLEKHB2 | 3.0 | 3.1 | 3.1 |
| 202096_s_at | translocator protein (18kDa) | TSPO | 3.3 | 2.9 | 3.1 |
| 203570_at | lysyl oxidase-like 1 | LOXL1 | 4.1 | 2.4 | 3.1 |
| 204163_at | elastin microfibril interfacer 1 | EMILIN1 | 4.0 | 2.5 | 3.1 |
| 204860_s_at | baculoviral IAP repeat-containing protein 1-like  NLR family, apoptosis inhibitory protein | LOC100510692 NAIP | 3.6 | 2.7 | 3.1 |
| 205434_s_at | AP2 associated kinase 1 | AAK1 | 3.6 | 2.7 | 3.1 |
| 207826_s_at | inhibitor of DNA binding 3, dominant negative helix-loop-helix protein | ID3 | 4.2 | 2.3 | 3.1 |
| 208131_s_at | prostaglandin I2 (prostacyclin) synthase | PTGIS | 4.7 | 2.0 | 3.1 |
| 208450_at | lectin, galactoside-binding, soluble, 2 | LGALS2 | 4.0 | 2.4 | 3.1 |
| 209356_x_at | EGF-containing fibulin-like extracellular matrix protein 2 | EFEMP2 | 4.1 | 2.3 | 3.1 |
| 210554_s_at | C-terminal binding protein 2 | CTBP2 | 4.2 | 2.3 | 3.1 |
| 210589_s_at | glucosidase, beta, acid pseudogene 1 | GBAP1 | 3.6 | 2.7 | 3.1 |
| 210986_s_at | tropomyosin 1 (alpha) | TPM1 | 3.0 | 3.1 | 3.1 |
| 212013_at | peroxidasin homolog (Drosophila) | PXDN | 4.2 | 2.3 | 3.1 |
| 212191_x_at | ribosomal protein L13 | RPL13 | 3.5 | 2.7 | 3.1 |
| 213422_s_at | matrix-remodelling associated 8 | MXRA8 | 4.3 | 2.3 | 3.1 |
| 214602_at | collagen, type IV, alpha 4 | COL4A4 | 4.5 | 2.1 | 3.1 |
| 217755_at | hematological and neurological expressed 1 | HN1 | 2.1 | 4.4 | 3.1 |
| 218223_s_at | pleckstrin homology domain containing, family O member 1 | PLEKHO1 | 3.5 | 2.8 | 3.1 |
| 218332_at | brain expressed, X-linked 1 | BEX1 | 4.3 | 2.1 | 3.1 |
| 219161_s_at | chemokine-like factor | CKLF | 3.3 | 3.0 | 3.1 |
| 219593_at | solute carrier family 15, member 3 | SLC15A3 | 3.4 | 2.8 | 3.1 |
| 219717_at | DDB1 and CUL4 associated factor 16 | DCAF16 | 3.5 | 2.7 | 3.1 |
| 219812_at | poliovirus receptor related immunoglobulin domain containing | PVRIG | 3.1 | 3.1 | 3.1 |
| 219885_at | schlafen family member 12 | SLFN12 | 4.2 | 2.3 | 3.1 |
| 221756_at | phosphoinositide-3-kinase interacting protein 1 | PIK3IP1 | 3.9 | 2.5 | 3.1 |
| 224733_at | CKLF-like MARVEL transmembrane domain containing 3 | CMTM3 | 3.4 | 2.7 | 3.1 |
| 225173_at | Rho GTPase activating protein 18 | ARHGAP18 | 3.8 | 2.5 | 3.1 |
| 225372_at | chromosome 10 open reading frame 54 | C10orf54 | 3.8 | 2.5 | 3.1 |
| 225540_at | microtubule-associated protein 2 | MAP2 | 4.3 | 2.3 | 3.1 |
| 226403_at | transmembrane channel-like 4 | TMC4 | 4.4 | 2.2 | 3.1 |
| 226950_at | activin A receptor type II-like 1 | ACVRL1 | 3.7 | 2.5 | 3.1 |
| 227084_at | dystrobrevin, alpha | DTNA | 3.0 | 3.1 | 3.1 |
| 227152_at | chromosome 12 open reading frame 35 | C12orf35 | 3.4 | 2.8 | 3.1 |
| 235213_at | Inositol 1,4,5-trisphosphate 3-kinase B | ITPKB | 4.0 | 2.5 | 3.1 |
| 241803_s_at | hypothetical LOC401522 | LOC401522 | 5.7 | 1.6 | 3.1 |
| 1556051_a_at | bicaudal D homolog 1 (Drosophila) | BICD1 | 3.2 | 2.8 | 3.0 |
| 1556656_at | --- | --- | 3.2 | 2.9 | 3.0 |
| 200808_s_at | zyxin | ZYX | 2.8 | 3.2 | 3.0 |
| 200859_x_at | filamin A, alpha | FLNA | 3.5 | 2.6 | 3.0 |
| 200872_at | S100 calcium binding protein A10 | S100A10 | 2.9 | 3.1 | 3.0 |
| 201050_at | phospholipase D family, member 3 | PLD3 | 3.3 | 2.8 | 3.0 |
| 201212_at | legumain | LGMN | 3.6 | 2.5 | 3.0 |
| 201299_s_at | MOB1, Mps One Binder kinase activator-like 1B (yeast) | MOBKL1B | 3.5 | 2.5 | 3.0 |
| 201774_s_at | non-SMC condensin I complex, subunit D2 | NCAPD2 | 2.9 | 3.0 | 3.0 |
| 201798_s_at | myoferlin | MYOF | 3.3 | 2.7 | 3.0 |
| 202551_s_at | cysteine rich transmembrane BMP regulator 1 (chordin-like) | CRIM1 | 3.4 | 2.6 | 3.0 |
| 204328_at | transmembrane channel-like 6 | TMC6 | 3.5 | 2.6 | 3.0 |
| 204345_at | collagen, type XVI, alpha 1 | COL16A1 | 4.0 | 2.2 | 3.0 |
| 205406_s_at | sperm autoantigenic protein 17 | SPA17 | 2.9 | 3.1 | 3.0 |
| 205596_s_at | SMAD specific E3 ubiquitin protein ligase 2 | SMURF2 | 3.5 | 2.6 | 3.0 |
| 205786_s_at | integrin, alpha M (complement component 3 receptor 3 subunit) | ITGAM | 4.0 | 2.3 | 3.0 |
| 207339_s_at | lymphotoxin beta (TNF superfamily, member 3) | LTB | 3.8 | 2.4 | 3.0 |
| 208621_s_at | ezrin | EZR | 2.8 | 3.1 | 3.0 |
| 209827_s_at | interleukin 16 (lymphocyte chemoattractant factor) | IL16 | 3.3 | 2.6 | 3.0 |
| 211896_s_at | decorin | DCN | 4.5 | 2.1 | 3.0 |
| 212733_at | KIAA0226 | KIAA0226 | 3.2 | 2.8 | 3.0 |
| 213226_at | cyclin A2 | CCNA2 | 2.1 | 4.2 | 3.0 |
| 218388_at | 6-phosphogluconolactonase | PGLS | 3.1 | 2.9 | 3.0 |
| 218995_s_at | endothelin 1 | EDN1 | 4.0 | 2.3 | 3.0 |
| 219229_at | solute carrier organic anion transporter family, member 3A1 | SLCO3A1 | 3.9 | 2.3 | 3.0 |
| 219551_at | ELL associated factor 2 | EAF2 | 4.5 | 1.9 | 3.0 |
| 222858_s_at | dual adaptor of phosphotyrosine and 3-phosphoinositides | DAPP1 | 3.6 | 2.5 | 3.0 |
| 225029_at | hypothetical LOC550643 | LOC550643 | 3.4 | 2.7 | 3.0 |
| 225258_at | filamin binding LIM protein 1 | FBLIM1 | 3.0 | 3.0 | 3.0 |
| 225629_s_at | zinc finger and BTB domain containing 4 | ZBTB4 | 3.9 | 2.3 | 3.0 |
| 226164_x_at | Ribosomal modification protein rimK-like family member B | RIMKLB | 4.1 | 2.2 | 3.0 |
| 227030_at | IKAROS family zinc finger 3 (Aiolos) | IKZF3 | 3.2 | 2.8 | 3.0 |
| 227145_at | lysyl oxidase-like 4 | LOXL4 | 2.8 | 3.2 | 3.0 |
| 227272_at | chromosome 15 open reading frame 52 | C15orf52 | 4.2 | 2.1 | 3.0 |
| 228141_at | glutathione peroxidase 8 (putative) | GPX8 | 4.2 | 2.2 | 3.0 |
| 228410_at | GRB2-associated binding protein 3 | GAB3 | 3.5 | 2.5 | 3.0 |
| 228497_at | solute carrier family 22, member 15 | SLC22A15 | 2.4 | 3.8 | 3.0 |
| 236533_at | ArfGAP with SH3 domain, ankyrin repeat and PH domain 1 | ASAP1 | 3.2 | 2.8 | 3.0 |
| 238593_at | chromosome 11 open reading frame 80 | C11orf80 | 3.3 | 2.7 | 3.0 |
| 242903_at | interferon gamma receptor 1 | IFNGR1 | 2.6 | 3.4 | 3.0 |
| 1557553_at | protein phosphatase 1, regulatory (inhibitor) subunit 12B | PPP1R12B | 3.2 | 2.7 | 2.9 |
| 1559051_s_at | chromosome 6 open reading frame 150 | C6orf150 | 3.4 | 2.4 | 2.9 |
| 1569652_at | myeloid/lymphoid or mixed-lineage leukemia (trithorax homolog, Drosophila); translocated to, 3 | MLLT3 | 3.5 | 2.4 | 2.9 |
| 200983_x_at | CD59 molecule, complement regulatory protein | CD59 | 3.3 | 2.5 | 2.9 |
| 201613_s_at | adaptor-related protein complex 1, gamma 2 subunit | AP1G2 | 3.3 | 2.5 | 2.9 |
| 202748_at | guanylate binding protein 2, interferon-inducible | GBP2 | 3.3 | 2.5 | 2.9 |
| 203185_at | Ras association (RalGDS/AF-6) domain family member 2 | RASSF2 | 3.5 | 2.4 | 2.9 |
| 203693_s_at | E2F transcription factor 3 | E2F3 | 2.9 | 3.0 | 2.9 |
| 204116_at | interleukin 2 receptor, gamma | IL2RG | 3.1 | 2.8 | 2.9 |
| 204285_s_at | phorbol-12-myristate-13-acetate-induced protein 1 | PMAIP1 | 3.3 | 2.7 | 2.9 |
| 204341_at | tripartite motif-containing 16 | TRIM16 | 1.5 | 5.7 | 2.9 |
| 204962_s_at | centromere protein A | CENPA | 2.1 | 4.1 | 2.9 |
| 205436_s_at | H2A histone family, member X | H2AFX | 2.2 | 3.9 | 2.9 |
| 208627_s_at | Y box binding protein 1 | YBX1 | 2.8 | 3.0 | 2.9 |
| 209365_s_at | extracellular matrix protein 1 | ECM1 | 3.8 | 2.2 | 2.9 |
| 210031_at | CD247 molecule | CD247 | 2.6 | 3.2 | 2.9 |
| 210908_s_at | prefoldin subunit 5 | PFDN5 | 3.4 | 2.5 | 2.9 |
| 211474_s_at | serpin peptidase inhibitor, clade B (ovalbumin), member 6 | SERPINB6 | 3.6 | 2.4 | 2.9 |
| 211529_x_at | major histocompatibility complex, class I, G | HLA-G | 3.0 | 2.7 | 2.9 |
| 212959_s_at | N-acetylglucosamine-1-phosphate transferase, alpha and beta subunits | GNPTAB | 3.4 | 2.5 | 2.9 |
| 213428_s_at | collagen, type VI, alpha 1 | COL6A1 | 3.5 | 2.4 | 2.9 |
| 214250_at | nuclear mitotic apparatus protein 1 | NUMA1 | 3.8 | 2.2 | 2.9 |
| 218454_at | phospholipase B domain containing 1 | PLBD1 | 3.6 | 2.3 | 2.9 |
| 218559_s_at | v-maf musculoaponeurotic fibrosarcoma oncogene homolog B (avian) | MAFB | 3.5 | 2.5 | 2.9 |
| 218724_s_at | TGFB-induced factor homeobox 2 | TGIF2 | 3.2 | 2.6 | 2.9 |
| 219064_at | inter-alpha (globulin) inhibitor H5 | ITIH5 | 4.1 | 2.1 | 2.9 |
| 219332_at | MICAL-like 2 | MICALL2 | 3.5 | 2.4 | 2.9 |
| 219952_s_at | mucolipin 1 | MCOLN1 | 3.0 | 2.8 | 2.9 |
| 220974_x_at | sideroflexin 3 | SFXN3 | 3.2 | 2.6 | 2.9 |
| 221011_s_at | limb bud and heart development homolog (mouse) | LBH | 3.4 | 2.4 | 2.9 |
| 221986_s_at | kelch-like 24 (Drosophila) | KLHL24 | 3.5 | 2.4 | 2.9 |
| 222587_s_at | UDP-N-acetyl-alpha-D-galactosamine:polypeptide N-acetylgalactosaminyltransferase 7 (GalNAc-T7) | GALNT7 | 4.1 | 2.1 | 2.9 |
| 223079_s_at | glutaminase | GLS | 3.4 | 2.5 | 2.9 |
| 223276_at | chromosome 5 open reading frame 62 | C5orf62 | 3.6 | 2.4 | 2.9 |
| 225303_at | kin of IRRE like (Drosophila) | KIRREL | 3.9 | 2.1 | 2.9 |
| 225655_at | ubiquitin-like with PHD and ring finger domains 1 | UHRF1 | 2.2 | 3.9 | 2.9 |
| 225685_at | CDC42 effector protein (Rho GTPase binding) 3 | CDC42EP3 | 3.7 | 2.4 | 2.9 |
| 225710_at | guanine nucleotide binding protein (G protein), beta polypeptide 4 | GNB4 | 3.5 | 2.5 | 2.9 |
| 225776_at | RNA binding motif, single stranded interacting protein 2 | RBMS2 | 3.5 | 2.4 | 2.9 |
| 225898_at | WD repeat domain 54 | WDR54 | 3.5 | 2.4 | 2.9 |
| 226065_at | prickle homolog 1 (Drosophila) | PRICKLE1 | 4.2 | 2.0 | 2.9 |
| 226858_at | casein kinase 1, epsilon | CSNK1E | 3.3 | 2.6 | 2.9 |
| 226865_at | hypothetical LOC100509635 | LOC100509635 | 3.9 | 2.1 | 2.9 |
| 226899_at | unc-5 homolog B (C. elegans) | UNC5B | 3.9 | 2.2 | 2.9 |
| 228360_at | LY6/PLAUR domain containing 6B | LYPD6B | 4.4 | 1.9 | 2.9 |
| 229721_x_at | Der1-like domain family, member 3 | DERL3 | 3.3 | 2.6 | 2.9 |
| 230266_at | RAB7B, member RAS oncogene family | RAB7B | 3.1 | 2.8 | 2.9 |
| 231124_x_at | lymphocyte antigen 9 | LY9 | 3.2 | 2.6 | 2.9 |
| 232386_at | vacuolar protein sorting 13 homolog C (S. cerevisiae) | VPS13C | 3.5 | 2.4 | 2.9 |
| 232794_at | Hypothetical protein LOC153682 | LOC153682 | 4.0 | 2.1 | 2.9 |
| 235306_at | GTPase, IMAP family member 8 | GIMAP8 | 3.6 | 2.4 | 2.9 |
| 238430_x_at | schlafen family member 5 | SLFN5 | 3.4 | 2.4 | 2.9 |
| 242521_at | --- | --- | 3.3 | 2.5 | 2.9 |
| 244654_at | myosin IG | MYO1G | 3.3 | 2.5 | 2.9 |
| 64408_s_at | calmodulin-like 4 | CALML4 | 3.2 | 2.7 | 2.9 |
| 1556402_at | Similar to hCG1655084 | LOC100132741 | 4.7 | 1.6 | 2.8 |
| 200697_at | hexokinase 1 | HK1 | 3.3 | 2.4 | 2.8 |
| 201428_at | claudin 4 | CLDN4 | 2.8 | 2.8 | 2.8 |
| 203940_s_at | vasohibin 1 | VASH1 | 3.7 | 2.1 | 2.8 |
| 203988_s_at | fucosyltransferase 8 (alpha (1,6) fucosyltransferase) | FUT8 | 3.7 | 2.1 | 2.8 |
| 204099_at | SWI/SNF related, matrix associated, actin dependent regulator of chromatin, subfamily d, member 3 | SMARCD3 | 3.6 | 2.2 | 2.8 |
| 204158_s_at | T-cell, immune regulator 1, ATPase, H+ transporting, lysosomal V0 subunit A3 | TCIRG1 | 3.1 | 2.6 | 2.8 |
| 204215_at | chromosome 7 open reading frame 23 | C7orf23 | 3.3 | 2.4 | 2.8 |
| 204567_s_at | ATP-binding cassette, sub-family G (WHITE), member 1 | ABCG1 | 3.1 | 2.5 | 2.8 |
| 204610_s_at | coiled-coil domain containing 85B | CCDC85B | 3.5 | 2.3 | 2.8 |
| 204735_at | phosphodiesterase 4A, cAMP-specific | PDE4A | 3.1 | 2.5 | 2.8 |
| 205119_s_at | formyl peptide receptor 1 | FPR1 | 3.0 | 2.7 | 2.8 |
| 205290_s_at | bone morphogenetic protein 2 | BMP2 | 3.3 | 2.5 | 2.8 |
| 205582_s_at | gamma-glutamyltransferase 5 | GGT5 | 3.8 | 2.0 | 2.8 |
| 205659_at | histone deacetylase 9 | HDAC9 | 3.3 | 2.4 | 2.8 |
| 205872_x_at | phosphodiesterase 4D interacting protein | PDE4DIP | 2.6 | 3.0 | 2.8 |
| 207008_at | chemokine (C-X-C motif) receptor 2 | CXCR2 | 3.4 | 2.3 | 2.8 |
| 207169_x_at | discoidin domain receptor tyrosine kinase 1 | DDR1 | 3.4 | 2.4 | 2.8 |
| 207237_at | potassium voltage-gated channel, shaker-related subfamily, member 3 | KCNA3 | 3.3 | 2.4 | 2.8 |
| 209539_at | Rac/Cdc42 guanine nucleotide exchange factor (GEF) 6 | ARHGEF6 | 3.5 | 2.3 | 2.8 |
| 213125_at | olfactomedin-like 2B | OLFML2B | 3.5 | 2.2 | 2.8 |
| 215446_s_at | lysyl oxidase | LOX | 3.5 | 2.3 | 2.8 |
| 218805_at | GTPase, IMAP family member 5 | GIMAP5 | 3.4 | 2.3 | 2.8 |
| 219073_s_at | oxysterol binding protein-like 10 | OSBPL10 | 3.6 | 2.1 | 2.8 |
| 219282_s_at | transient receptor potential cation channel, subfamily V, member 2 | TRPV2 | 3.0 | 2.5 | 2.8 |
| 219474_at | chromosome 3 open reading frame 52 | C3orf52 | 2.6 | 3.1 | 2.8 |
| 219697_at | heparan sulfate (glucosamine) 3-O-sulfotransferase 2 | HS3ST2 | 3.1 | 2.5 | 2.8 |
| 219734_at | SID1 transmembrane family, member 1 | SIDT1 | 2.8 | 2.7 | 2.8 |
| 220110_s_at | nuclear RNA export factor 3 | NXF3 | 3.7 | 2.1 | 2.8 |
| 220418_at | ubiquitin associated and SH3 domain containing A | UBASH3A | 3.1 | 2.5 | 2.8 |
| 221969_at | paired box 5 | PAX5 | 3.7 | 2.1 | 2.8 |
| 222457_s_at | LIM domain and actin binding 1 | LIMA1 | 3.1 | 2.5 | 2.8 |
| 223423_at | G protein-coupled receptor 160 | GPR160 | 3.4 | 2.4 | 2.8 |
| 224929_at | transmembrane protein 173 | TMEM173 | 3.7 | 2.0 | 2.8 |
| 224997_x_at | H19, imprinted maternally expressed transcript (non-protein coding) | H19 | 3.4 | 2.3 | 2.8 |
| 225273_at | WWC family member 3 | WWC3 | 3.2 | 2.4 | 2.8 |
| 225841_at | chromosome 1 open reading frame 59 | C1orf59 | 3.3 | 2.4 | 2.8 |
| 226047_at | murine retrovirus integration site 1 homolog | MRVI1 | 4.1 | 1.9 | 2.8 |
| 227134_at | synaptotagmin-like 1 | SYTL1 | 3.3 | 2.4 | 2.8 |
| 227839_at | methyl-CpG binding domain protein 5 | MBD5 | 4.1 | 1.8 | 2.8 |
| 227955_s_at | ephrin-A5 | EFNA5 | 3.9 | 2.0 | 2.8 |
| 228058_at | zymogen granule protein 16 homolog B (rat) | ZG16B | 3.8 | 2.1 | 2.8 |
| 228080_at | layilin | LAYN | 3.6 | 2.1 | 2.8 |
| 228962_at | phosphodiesterase 4D, cAMP-specific | PDE4D | 2.8 | 2.8 | 2.8 |
| 230748_at | solute carrier family 16, member 6 (monocarboxylic acid transporter 7) | SLC16A6 | 2.8 | 2.8 | 2.8 |
| 232024_at | GTPase, IMAP family member 2 | GIMAP2 | 3.5 | 2.3 | 2.8 |
| 232311_at | Beta-2-microglobulin | B2M | 3.2 | 2.5 | 2.8 |
| 235670_at | syntaxin 11 | STX11 | 3.1 | 2.5 | 2.8 |
| 236044_at | phosphatidic acid phosphatase type 2 domain containing 1A | PPAPDC1A | 4.2 | 1.8 | 2.8 |
| 37117_at | Rho GTPase activating protein 8  PRR5-ARHGAP8 readthrough | ARHGAP8 PRR5-ARHGAP8 | 4.0 | 2.0 | 2.8 |
| 1569607_s_at | ankyrin repeat domain 20 family, member A1  ankyrin repeat domain 20 family, member A2  ankyrin repeat domain 20 family, member A3  ankyrin repeat domain 20 family, member A4  ankyrin repeat domain 20 family, member A3 pseudogene ankyrin repeat domain-containing protein 20A3-like ankyrin repeat domain 20 family, member A2 pseudogene | ANKRD20A1 ANKRD20A2 ANKRD20A3 ANKRD20A4 C21orf81 LOC100132733 LOC284232 | 4.3 | 1.6 | 2.7 |
| 200760_s_at | ADP-ribosylation-like factor 6 interacting protein 5 | ARL6IP5 | 3.4 | 2.2 | 2.7 |
| 200909_s_at | ribosomal protein, large, P2 | RPLP2 | 3.0 | 2.3 | 2.7 |
| 201847_at | lipase A, lysosomal acid, cholesterol esterase | LIPA | 2.9 | 2.6 | 2.7 |
| 202095_s_at | baculoviral IAP repeat-containing 5 | BIRC5 | 1.7 | 4.2 | 2.7 |
| 202509_s_at | tumor necrosis factor, alpha-induced protein 2 | TNFAIP2 | 3.0 | 2.4 | 2.7 |
| 202826_at | serine peptidase inhibitor, Kunitz type 1 | SPINT1 | 3.3 | 2.2 | 2.7 |
| 203562_at | fasciculation and elongation protein zeta 1 (zygin I) | FEZ1 | 3.7 | 1.9 | 2.7 |
| 203757_s_at | carcinoembryonic antigen-related cell adhesion molecule 6 (non-specific cross reacting antigen) | CEACAM6 | 3.7 | 2.0 | 2.7 |
| 203766_s_at | leiomodin 1 (smooth muscle) | LMOD1 | 2.9 | 2.5 | 2.7 |
| 203835_at | leucine rich repeat containing 32 | LRRC32 | 3.4 | 2.2 | 2.7 |
| 204019_s_at | SH3 domain containing, Ysc84-like 1 (S. cerevisiae) | SH3YL1 | 4.1 | 1.7 | 2.7 |
| 204929_s_at | vesicle-associated membrane protein 5 (myobrevin) | VAMP5 | 3.1 | 2.4 | 2.7 |
| 205376_at | inositol polyphosphate-4-phosphatase, type II, 105kDa | INPP4B | 3.7 | 2.0 | 2.7 |
| 206028_s_at | c-mer proto-oncogene tyrosine kinase | MERTK | 2.8 | 2.5 | 2.7 |
| 208711_s_at | cyclin D1 | CCND1 | 1.6 | 4.6 | 2.7 |
| 209099_x_at | jagged 1 | JAG1 | 3.3 | 2.2 | 2.7 |
| 209761_s_at | SP110 nuclear body protein | SP110 | 3.2 | 2.3 | 2.7 |
| 209892_at | fucosyltransferase 4 (alpha (1,3) fucosyltransferase, myeloid-specific) | FUT4 | 3.0 | 2.5 | 2.7 |
| 210279_at | G protein-coupled receptor 18 | GPR18 | 3.4 | 2.1 | 2.7 |
| 210657_s_at | septin 4 | SEPT4 | 3.4 | 2.1 | 2.7 |
| 211071_s_at | myeloid/lymphoid or mixed-lineage leukemia (trithorax homolog, Drosophila); translocated to, 11 | MLLT11 | 2.1 | 3.5 | 2.7 |
| 212119_at | ras homolog gene family, member Q | RHOQ | 3.2 | 2.3 | 2.7 |
| 212288_at | formin binding protein 1 | FNBP1 | 3.1 | 2.4 | 2.7 |
| 213923_at | RAP2B, member of RAS oncogene family | RAP2B | 3.2 | 2.3 | 2.7 |
| 213998_s_at | DEAD (Asp-Glu-Ala-Asp) box polypeptide 17 | DDX17 | 3.6 | 2.0 | 2.7 |
| 214459_x_at | major histocompatibility complex, class I, C | HLA-C | 3.0 | 2.5 | 2.7 |
| 214659_x_at | YLP motif containing 1 | YLPM1 | 3.1 | 2.3 | 2.7 |
| 216840_s_at | laminin, alpha 2 | LAMA2 | 3.8 | 1.9 | 2.7 |
| 217428_s_at | collagen, type X, alpha 1 | COL10A1 | 3.8 | 1.9 | 2.7 |
| 218261_at | adaptor-related protein complex 1, mu 2 subunit | AP1M2 | 2.7 | 2.6 | 2.7 |
| 218831_s_at | Fc fragment of IgG, receptor, transporter, alpha | FCGRT | 3.4 | 2.1 | 2.7 |
| 219789_at | natriuretic peptide receptor C/guanylate cyclase C (atrionatriuretic peptide receptor C) | NPR3 | 4.0 | 1.8 | 2.7 |
| 221558_s_at | lymphoid enhancer-binding factor 1 | LEF1 | 3.0 | 2.3 | 2.7 |
| 221667_s_at | heat shock 22kDa protein 8 | HSPB8 | 2.9 | 2.6 | 2.7 |
| 223201_s_at | transmembrane protein 164 | TMEM164 | 2.8 | 2.6 | 2.7 |
| 223809_at | regulator of G-protein signaling 18 | RGS18 | 2.8 | 2.5 | 2.7 |
| 224496_s_at | transmembrane protein 107 | TMEM107 | 3.1 | 2.4 | 2.7 |
| 224981_at | transmembrane protein 219 | TMEM219 | 3.0 | 2.5 | 2.7 |
| 226272_at | RCAN family member 3 | RCAN3 | 3.1 | 2.4 | 2.7 |
| 226675_s_at | metastasis associated lung adenocarcinoma transcript 1 (non-protein coding) | MALAT1 | 2.7 | 2.7 | 2.7 |
| 227210_at | Scm-like with four mbt domains 2 | SFMBT2 | 4.0 | 1.9 | 2.7 |
| 228531_at | sterile alpha motif domain containing 9 | SAMD9 | 3.5 | 2.1 | 2.7 |
| 228667_at | 1-acylglycerol-3-phosphate O-acyltransferase 4 (lysophosphatidic acid acyltransferase, delta) | AGPAT4 | 3.3 | 2.3 | 2.7 |
| 230011_at | meiosis inhibitor 1 | MEI1 | 3.0 | 2.4 | 2.7 |
| 230529_at | headcase homolog (Drosophila) | HECA | 3.4 | 2.1 | 2.7 |
| 230669_at | RAS p21 protein activator 2 | RASA2 | 2.9 | 2.5 | 2.7 |
| 231108_at | fused in sarcoma | FUS | 3.2 | 2.3 | 2.7 |
| 231776_at | eomesodermin | EOMES | 3.3 | 2.3 | 2.7 |
| 240168_at | exportin 7 | XPO7 | 3.1 | 2.4 | 2.7 |
| 242208_at | zinc finger protein 37B, pseudogene | ZNF37BP | 3.2 | 2.3 | 2.7 |
| 1556203_a_at | SLIT-ROBO Rho GTPase activating protein 2 | SRGAP2 | 2.9 | 2.4 | 2.6 |
| 1559957_a_at | hypothetical LOC642852 | LOC642852 | 2.6 | 2.5 | 2.6 |
| 1562028_at | Cyclin D3 | CCND3 | 2.5 | 2.7 | 2.6 |
| 200600_at | moesin | MSN | 2.7 | 2.5 | 2.6 |
| 200905_x_at | major histocompatibility complex, class I, E | HLA-E | 3.0 | 2.3 | 2.6 |
| 201162_at | insulin-like growth factor binding protein 7 | IGFBP7 | 3.3 | 2.0 | 2.6 |
| 201189_s_at | inositol 1,4,5-triphosphate receptor, type 3 | ITPR3 | 2.7 | 2.5 | 2.6 |
| 204033_at | thyroid hormone receptor interactor 13 | TRIP13 | 1.6 | 4.3 | 2.6 |
| 204528_s_at | nucleosome assembly protein 1-like 1 | NAP1L1 | 2.9 | 2.4 | 2.6 |
| 204933_s_at | tumor necrosis factor receptor superfamily, member 11b | TNFRSF11B | 2.5 | 2.7 | 2.6 |
| 205092_x_at | zinc finger and BTB domain containing 1 | ZBTB1 | 2.8 | 2.4 | 2.6 |
| 206197_at | non-metastatic cells 5, protein expressed in (nucleoside-diphosphate kinase) | NME5 | 3.8 | 1.9 | 2.6 |
| 206219_s_at | vav 1 guanine nucleotide exchange factor | VAV1 | 3.2 | 2.1 | 2.6 |
| 206631_at | prostaglandin E receptor 2 (subtype EP2), 53kDa | PTGER2 | 2.7 | 2.5 | 2.6 |
| 206682_at | C-type lectin domain family 10, member A | CLEC10A | 3.2 | 2.1 | 2.6 |
| 208782_at | follistatin-like 1 | FSTL1 | 3.3 | 2.1 | 2.6 |
| 210166_at | toll-like receptor 5 | TLR5 | 3.4 | 2.0 | 2.6 |
| 212658_at | lipoma HMGIC fusion partner-like 2 | LHFPL2 | 3.0 | 2.2 | 2.6 |
| 214180_at | mannosidase, alpha, class 1C, member 1 | MAN1C1 | 3.8 | 1.8 | 2.6 |
| 215780_s_at | SET translocation (myeloid leukemia-associated) pseudogene  SET nuclear oncogene | LOC642869  SET | 2.4 | 2.8 | 2.6 |
| 218094_s_at | dysbindin (dystrobrevin binding protein 1) domain containing 2  SYS1 Golgi-localized integral membrane protein homolog (S. cerevisiae)  SYS1-DBNDD2 read-through transcript | DBNDD2  SYS1  SYS1-DBNDD2 | 3.3 | 2.1 | 2.6 |
| 218717_s_at | leprecan-like 1 | LEPREL1 | 3.2 | 2.2 | 2.6 |
| 218880_at | FOS-like antigen 2 | FOSL2 | 1.8 | 3.8 | 2.6 |
| 219202_at | rhomboid 5 homolog 2 (Drosophila) | RHBDF2 | 2.8 | 2.4 | 2.6 |
| 219423_x_at | tumor necrosis factor receptor superfamily, member 25 | TNFRSF25 | 3.0 | 2.2 | 2.6 |
| 219978_s_at | nucleolar and spindle associated protein 1 | NUSAP1 | 2.2 | 3.1 | 2.6 |
| 220122_at | multiple C2 domains, transmembrane 1 | MCTP1 | 3.7 | 1.8 | 2.6 |
| 223095_at | MARVEL domain containing 1 | MARVELD1 | 3.1 | 2.1 | 2.6 |
| 223241_at | sorting nexin 8 | SNX8 | 3.0 | 2.2 | 2.6 |
| 223316_at | coiled-coil domain containing 3 | CCDC3 | 3.5 | 1.9 | 2.6 |
| 223655_at | CD163 molecule-like 1 | CD163L1 | 2.4 | 2.7 | 2.6 |
| 223773_s_at | small nucleolar RNA host gene 12 (non-protein coding) | SNHG12 | 3.0 | 2.2 | 2.6 |
| 224841_x_at | growth arrest-specific 5 (non-protein coding) | GAS5 | 3.2 | 2.1 | 2.6 |
| 225185_at | muscle RAS oncogene homolog | MRAS | 2.9 | 2.3 | 2.6 |
| 225325_at | major facilitator superfamily domain containing 6 | MFSD6 | 2.9 | 2.3 | 2.6 |
| 225660_at | sema domain, transmembrane domain (TM), and cytoplasmic domain, (semaphorin) 6A | SEMA6A | 3.6 | 1.9 | 2.6 |
| 226789_at | embigin | EMB | 3.1 | 2.1 | 2.6 |
| 226835_s_at | non-protein coding RNA 275 | NCRNA00275 | 2.9 | 2.3 | 2.6 |
| 226992_at | nitric oxide synthase trafficker | NOSTRIN | 3.0 | 2.2 | 2.6 |
| 227087_at | inositol polyphosphate-4-phosphatase, type I, 107kDa | INPP4A | 3.0 | 2.3 | 2.6 |
| 227183_at | hypothetical LOC728264 | LOC728264 | 2.7 | 2.5 | 2.6 |
| 227394_at | neural cell adhesion molecule 1 | NCAM1 | 4.1 | 1.6 | 2.6 |
| 227919_at | urothelial cancer associated 1 (non-protein coding) | UCA1 | 3.7 | 1.8 | 2.6 |
| 228069_at | family with sequence similarity 54, member A | FAM54A | 2.1 | 3.2 | 2.6 |
| 228186_s_at | R-spondin 3 homolog (Xenopus laevis) | RSPO3 | 4.3 | 1.6 | 2.6 |
| 228245_s_at | ovostatin homolog 2-like  ovostatin homolog 2-like  ovostatin 2 | LOC100509231 LOC100509288 OVOS2 | 2.7 | 2.5 | 2.6 |
| 230245_s_at | hypothetical LOC283663 | LOC283663 | 3.5 | 2.0 | 2.6 |
| 230563_at | RasGEF domain family, member 1A | RASGEF1A | 3.2 | 2.0 | 2.6 |
| 236595_at | hypothetical LOC100507307 | LOC100507307 | 2.9 | 2.3 | 2.6 |
| 238439_at | ankyrin repeat domain 22 | ANKRD22 | 2.8 | 2.3 | 2.6 |
| 241694_at | polycystic kidney and hepatic disease 1 (autosomal recessive) | PKHD1 | 4.0 | 1.7 | 2.6 |
| 242916_at | centrosomal protein 110kDa | CEP110 | 2.9 | 2.3 | 2.6 |
| 243109_at | multiple C2 domains, transmembrane 2 | MCTP2 | 2.9 | 2.3 | 2.6 |
| 35974_at | lymphoid-restricted membrane protein | LRMP | 3.1 | 2.1 | 2.6 |
| 41577_at | protein phosphatase 1, regulatory (inhibitor) subunit 16B | PPP1R16B | 3.2 | 2.2 | 2.6 |
| 1552263_at | mitogen-activated protein kinase 1 | MAPK1 | 2.5 | 2.6 | 2.5 |
| 1554690_a_at | transforming, acidic coiled-coil containing protein 1 | TACC1 | 3.3 | 1.9 | 2.5 |
| 1555842_at | cytohesin 2 | CYTH2 | 2.8 | 2.2 | 2.5 |
| 1555852_at | hypothetical LOC100507463 | LOC100507463 | 3.1 | 2.0 | 2.5 |
| 1557749_at | EH domain binding protein 1-like 1 | EHBP1L1 | 2.9 | 2.2 | 2.5 |
| 1558515_at | non-protein coding RNA 182 | NCRNA00182 | 2.9 | 2.2 | 2.5 |
| 1558693_s_at | chromosome 1 open reading frame 85 | C1orf85 | 2.6 | 2.4 | 2.5 |
| 200748_s_at | ferritin, heavy polypeptide 1 | FTH1 | 2.3 | 2.7 | 2.5 |
| 200762_at | dihydropyrimidinase-like 2 | DPYSL2 | 3.2 | 1.9 | 2.5 |
| 200772_x_at | prothymosin, alpha | PTMA | 2.6 | 2.5 | 2.5 |
| 201311_s_at | SH3 domain binding glutamic acid-rich protein like | SH3BGRL | 3.2 | 1.9 | 2.5 |
| 202107_s_at | minichromosome maintenance complex component 2 | MCM2 | 2.0 | 3.0 | 2.5 |
| 202381_at | ADAM metallopeptidase domain 9 | ADAM9 | 2.8 | 2.3 | 2.5 |
| 202669_s_at | ephrin-B2 | EFNB2 | 2.7 | 2.2 | 2.5 |
| 203034_s_at | ribosomal protein L27a | RPL27A | 2.6 | 2.3 | 2.5 |
| 203065_s_at | caveolin 1, caveolae protein, 22kDa | CAV1 | 3.1 | 2.0 | 2.5 |
| 204254_s_at | vitamin D (1,25- dihydroxyvitamin D3) receptor | VDR | 2.7 | 2.4 | 2.5 |
| 204559_s_at | LSM7 homolog, U6 small nuclear RNA associated (S. cerevisiae) | LSM7 | 2.5 | 2.5 | 2.5 |
| 204570_at | cytochrome c oxidase subunit VIIa polypeptide 1 (muscle) | COX7A1 | 3.5 | 1.9 | 2.5 |
| 204931_at | transcription factor 21 | TCF21 | 3.4 | 1.8 | 2.5 |
| 205137_x_at | Usher syndrome 1C (autosomal recessive, severe) | USH1C | 2.9 | 2.2 | 2.5 |
| 205266_at | leukemia inhibitory factor (cholinergic differentiation factor) | LIF | 2.2 | 2.9 | 2.5 |
| 205308_at | family with sequence similarity 164, member A | FAM164A | 3.4 | 1.9 | 2.5 |
| 205379_at | carbonyl reductase 3 | CBR3 | 2.1 | 2.9 | 2.5 |
| 207233_s_at | microphthalmia-associated transcription factor | MITF | 3.2 | 1.9 | 2.5 |
| 208699_x_at | transketolase | TKT | 1.9 | 3.3 | 2.5 |
| 208797_s_at | golgin A8 family, member A | GOLGA8A | 2.7 | 2.4 | 2.5 |
| 209282_at | protein kinase D2 | PRKD2 | 2.7 | 2.2 | 2.5 |
| 210510_s_at | neuropilin 1 | NRP1 | 3.1 | 2.1 | 2.5 |
| 211047_x_at | adaptor-related protein complex 2, sigma 1 subunit | AP2S1 | 2.7 | 2.4 | 2.5 |
| 211993_at | WNK lysine deficient protein kinase 1 | WNK1 | 2.6 | 2.5 | 2.5 |
| 212420_at | E74-like factor 1 (ets domain transcription factor) | ELF1 | 2.8 | 2.2 | 2.5 |
| 212647_at | related RAS viral (r-ras) oncogene homolog | RRAS | 3.0 | 2.0 | 2.5 |
| 213004_at | angiopoietin-like 2 | ANGPTL2 | 3.2 | 1.9 | 2.5 |
| 213183_s_at | Cyclin-dependent kinase inhibitor 1C (p57, Kip2) | CDKN1C | 4.2 | 1.5 | 2.5 |
| 213988_s_at | spermidine/spermine N1-acetyltransferase 1 | SAT1 | 2.7 | 2.3 | 2.5 |
| 214239_x_at | polycomb group ring finger 2 | PCGF2 | 3.8 | 1.7 | 2.5 |
| 216333_x_at | tenascin XA pseudogene  tenascin XB | TNXA  TNXB | 3.9 | 1.6 | 2.5 |
| 218847_at | insulin-like growth factor 2 mRNA binding protein 2 | IGF2BP2 | 3.0 | 2.0 | 2.5 |
| 218885_s_at | UDP-N-acetyl-alpha-D-galactosamine:polypeptide N-acetylgalactosaminyltransferase 12 (GalNAc-T12) | GALNT12 | 3.2 | 1.9 | 2.5 |
| 219210_s_at | RAB8B, member RAS oncogene family | RAB8B | 2.7 | 2.3 | 2.5 |
| 219404_at | EPS8-like 3 | EPS8L3 | 1.4 | 4.5 | 2.5 |
| 219407_s_at | laminin, gamma 3 | LAMC3 | 3.2 | 1.9 | 2.5 |
| 219429_at | fatty acid 2-hydroxylase | FA2H | 2.9 | 2.3 | 2.5 |
| 220609_at | hypothetical LOC202181 | LOC202181 | 3.1 | 1.9 | 2.5 |
| 220918_at | chromosome 21 open reading frame 96 | C21orf96 | 3.0 | 2.1 | 2.5 |
| 221081_s_at | DENN/MADD domain containing 2D | DENND2D | 2.9 | 2.1 | 2.5 |
| 221601_s_at | Fas apoptotic inhibitory molecule 3 | FAIM3 | 2.7 | 2.4 | 2.5 |
| 222557_at | stathmin-like 3 | STMN3 | 3.0 | 2.1 | 2.5 |
| 223741_s_at | tweety homolog 2 (Drosophila) | TTYH2 | 2.8 | 2.2 | 2.5 |
| 224890_s_at | chromosome 7 open reading frame 59 | C7orf59 | 3.0 | 2.1 | 2.5 |
| 225294_s_at | trafficking protein particle complex 1 | TRAPPC1 | 2.4 | 2.5 | 2.5 |
| 225597_at | solute carrier family 45, member 4 | SLC45A4 | 3.1 | 2.0 | 2.5 |
| 225870_s_at | trafficking protein particle complex 5 | TRAPPC5 | 2.5 | 2.4 | 2.5 |
| 226264_at | sushi domain containing 1 | SUSD1 | 3.0 | 2.1 | 2.5 |
| 226358_at | anterior pharynx defective 1 homolog B (C. elegans) | APH1B | 3.2 | 2.0 | 2.5 |
| 227485_at | DEAD/H (Asp-Glu-Ala-Asp/His) box polypeptide 26B | DDX26B | 3.1 | 2.1 | 2.5 |
| 228577_x_at | outer dense fiber of sperm tails 2-like | ODF2L | 3.4 | 1.9 | 2.5 |
| 230337_at | son of sevenless homolog 1 (Drosophila) | SOS1 | 2.9 | 2.2 | 2.5 |
| 231093_at | Fc receptor-like 3 | FCRL3 | 3.4 | 1.9 | 2.5 |
| 235709_at | growth arrest-specific 2 like 3 | GAS2L3 | 3.0 | 2.1 | 2.5 |
| 242093_at | synaptotagmin-like 5 | SYTL5 | 3.9 | 1.6 | 2.5 |
| 242974_at | CD47 molecule | CD47 | 2.7 | 2.4 | 2.5 |
| 336_at | thromboxane A2 receptor | TBXA2R | 3.3 | 1.8 | 2.5 |
| 40472_at | lysophosphatidylcholine acyltransferase 4 | LPCAT4 | 2.9 | 2.3 | 2.5 |
| 1557996_at | postmeiotic segregation increased 2-like 5-like | LOC100132832 | 2.8 | 2.1 | 2.4 |
| 1558747_at | structural maintenance of chromosomes flexible hinge domain containing 1 | SMCHD1 | 2.7 | 2.1 | 2.4 |
| 1560741_at | small nuclear ribonucleoprotein polypeptide N | SNRPN | 3.1 | 1.8 | 2.4 |
| 200629_at | tryptophanyl-tRNA synthetase | WARS | 2.3 | 2.5 | 2.4 |
| 200782_at | annexin A5 | ANXA5 | 2.6 | 2.2 | 2.4 |
| 201072_s_at | SWI/SNF related, matrix associated, actin dependent regulator of chromatin, subfamily c, member 1 | SMARCC1 | 2.4 | 2.5 | 2.4 |
| 201242_s_at | ATPase, Na+/K+ transporting, beta 1 polypeptide | ATP1B1 | 2.5 | 2.3 | 2.4 |
| 201360_at | cystatin C | CST3 | 3.0 | 1.9 | 2.4 |
| 201809_s_at | endoglin | ENG | 3.0 | 1.9 | 2.4 |
| 201871_s_at | UBX domain protein 1 | UBXN1 | 2.8 | 2.1 | 2.4 |
| 202450_s_at | cathepsin K | CTSK | 3.4 | 1.6 | 2.4 |
| 202598_at | S100 calcium binding protein A13 | S100A13 | 3.0 | 1.9 | 2.4 |
| 203066_at | carbohydrate (N-acetylgalactosamine 4-sulfate 6-O) sulfotransferase 15 | CHST15 | 3.1 | 2.0 | 2.4 |
| 203485_at | reticulon 1 | RTN1 | 3.2 | 1.8 | 2.4 |
| 203636_at | midline 1 (Opitz/BBB syndrome) | MID1 | 2.2 | 2.6 | 2.4 |
| 203999_at | synaptotagmin I | SYT1 | 3.3 | 1.7 | 2.4 |
| 204442_x_at | latent transforming growth factor beta binding protein 4 | LTBP4 | 2.8 | 2.2 | 2.4 |
| 204468_s_at | tyrosine kinase with immunoglobulin-like and EGF-like domains 1 | TIE1 | 2.9 | 2.0 | 2.4 |
| 204857_at | MAD1 mitotic arrest deficient-like 1 (yeast) | MAD1L1 | 2.3 | 2.5 | 2.4 |
| 205632_s_at | phosphatidylinositol-4-phosphate 5-kinase, type I, beta | PIP5K1B | 3.6 | 1.6 | 2.4 |
| 205936_s_at | hexokinase 3 (white cell) | HK3 | 3.2 | 1.8 | 2.4 |
| 206255_at | B lymphoid tyrosine kinase | BLK | 2.9 | 1.9 | 2.4 |
| 207420_at | collectin sub-family member 10 (C-type lectin) | COLEC10 | 3.1 | 1.9 | 2.4 |
| 209243_s_at | paternally expressed 3 | PEG3 | 3.4 | 1.7 | 2.4 |
| 209550_at | necdin homolog (mouse) | NDN | 3.4 | 1.7 | 2.4 |
| 209604_s_at | GATA binding protein 3 | GATA3 | 2.8 | 2.0 | 2.4 |
| 209897_s_at | slit homolog 2 (Drosophila) | SLIT2 | 2.7 | 2.2 | 2.4 |
| 210069_at | choline kinase-like, carnitine palmitoyltransferase 1B (muscle) transcription unit  carnitine palmitoyltransferase 1B (muscle) | CHKB-CPT1B  CPT1B | 3.1 | 1.8 | 2.4 |
| 210218_s_at | SP100 nuclear antigen | SP100 | 3.2 | 1.8 | 2.4 |
| 210858_x_at | ataxia telangiectasia mutated | ATM | 2.5 | 2.3 | 2.4 |
| 211275_s_at | glycogenin 1 | GYG1 | 2.8 | 2.1 | 2.4 |
| 211395_x_at | Fc fragment of IgG, low affinity IIc, receptor for (CD32) (gene/pseudogene) | FCGR2C | 3.3 | 1.8 | 2.4 |
| 211942_x_at | ribosomal protein L13a  ribosomal protein L13a pseudogene 5  ribosomal protein L13a pseudogene 6 | RPL13A RPL13AP5 RPL13AP6 | 2.6 | 2.3 | 2.4 |
| 212107_s_at | DEAH (Asp-Glu-Ala-His) box polypeptide 9 | DHX9 | 2.4 | 2.3 | 2.4 |
| 212220_at | proteasome (prosome, macropain) activator subunit 4 | PSME4 | 2.3 | 2.5 | 2.4 |
| 212826_s_at | solute carrier family 25 (mitochondrial carrier; adenine nucleotide translocator), member 6 | SLC25A6 | 2.6 | 2.3 | 2.4 |
| 213170_at | glutathione peroxidase 7 | GPX7 | 3.1 | 1.9 | 2.4 |
| 213326_at | vesicle-associated membrane protein 1 (synaptobrevin 1) | VAMP1 | 2.7 | 2.1 | 2.4 |
| 214686_at | zinc finger protein 266 | ZNF266 | 2.7 | 2.1 | 2.4 |
| 214749_s_at | armadillo repeat containing, X-linked 6 | ARMCX6 | 2.8 | 2.1 | 2.4 |
| 217436_x_at | major histocompatibility complex, class I, A  major histocompatibility complex, class I, F  major histocompatibility complex, class I, J (pseudogene) | HLA-A  HLA-F  HLA-J | 2.4 | 2.4 | 2.4 |
| 217610_at | --- | --- | 2.6 | 2.2 | 2.4 |
| 218826_at | solute carrier family 35, member F2 | SLC35F2 | 2.8 | 2.0 | 2.4 |
| 219278_at | mitogen-activated protein kinase kinase kinase 6 | MAP3K6 | 2.3 | 2.5 | 2.4 |
| 219296_at | zinc finger, DHHC-type containing 13 | ZDHHC13 | 2.9 | 2.1 | 2.4 |
| 219777_at | GTPase, IMAP family member 6 | GIMAP6 | 3.1 | 1.8 | 2.4 |
| 220289_s_at | absent in melanoma 1-like | AIM1L | 1.8 | 3.2 | 2.4 |
| 220615_s_at | fatty acyl CoA reductase 2 | FAR2 | 2.8 | 2.1 | 2.4 |
| 221261_x_at | melanoma antigen family D, 4  melanoma antigen family D, 4B | MAGED4 MAGED4B | 3.3 | 1.7 | 2.4 |
| 221521_s_at | GINS complex subunit 2 (Psf2 homolog) | GINS2 | 1.8 | 3.3 | 2.4 |
| 221704_s_at | vacuolar protein sorting 37 homolog B (S. cerevisiae) | VPS37B | 2.6 | 2.2 | 2.4 |
| 221840_at | protein tyrosine phosphatase, receptor type, E | PTPRE | 2.8 | 2.1 | 2.4 |
| 222634_s_at | transducin (beta)-like 1 X-linked receptor 1 | TBL1XR1 | 2.8 | 2.0 | 2.4 |
| 222750_s_at | steroid 5 alpha-reductase 3 | SRD5A3 | 2.4 | 2.5 | 2.4 |
| 223454_at | chemokine (C-X-C motif) ligand 16 | CXCL16 | 2.3 | 2.5 | 2.4 |
| 224414_s_at | caspase recruitment domain family, member 6 | CARD6 | 3.2 | 1.8 | 2.4 |
| 225081_s_at | cell division cycle associated 7-like | CDCA7L | 3.1 | 1.9 | 2.4 |
| 226663_at | ankyrin repeat domain 10 | ANKRD10 | 2.7 | 2.1 | 2.4 |
| 226926_at | dermokine | DMKN | 2.9 | 1.9 | 2.4 |
| 226936_at | centromere protein W | CENPW | 2.1 | 2.7 | 2.4 |
| 226975_at | RNA-binding region (RNP1, RRM) containing 3 | RNPC3 | 3.3 | 1.8 | 2.4 |
| 227230_s_at | KIAA1211 | KIAA1211 | 2.8 | 2.1 | 2.4 |
| 227330_x_at | hypothetical protein LOC100132288  hypothetical LOC100233156  MAFF interacting protein | LOC100132288 LOC100233156 MAFIP | 2.6 | 2.2 | 2.4 |
| 227791_at | solute carrier family 9 (sodium/hydrogen exchanger), member 9 | SLC9A9 | 3.2 | 1.9 | 2.4 |
| 227801_at | tripartite motif-containing 59 | TRIM59 | 2.3 | 2.5 | 2.4 |
| 228046_at | zinc finger protein 827 | ZNF827 | 3.3 | 1.8 | 2.4 |
| 228088_at | SEC14 and spectrin domains 1 | SESTD1 | 2.6 | 2.2 | 2.4 |
| 228298_at | family with sequence similarity 113, member B | FAM113B | 2.6 | 2.2 | 2.4 |
| 229669_at | hypothetical LOC100507263 | LOC100507263 | 2.5 | 2.4 | 2.4 |
| 230110_at | mucolipin 2 | MCOLN2 | 2.9 | 2.0 | 2.4 |
| 233827_s_at | suppressor of Ty 16 homolog (S. cerevisiae) | SUPT16H | 2.5 | 2.2 | 2.4 |
| 235352_at | major histocompatibility complex, class I-related | MR1 | 2.6 | 2.2 | 2.4 |
| 235522_at | C-type lectin domain family 2, member D | CLEC2D | 2.7 | 2.2 | 2.4 |
| 61734_at | reticulocalbin 3, EF-hand calcium binding domain | RCN3 | 2.9 | 2.0 | 2.4 |
| 74694_s_at | rabaptin, RAB GTPase binding effector protein 2 | RABEP2 | 2.5 | 2.3 | 2.4 |
| 1552767_a_at | heparan sulfate 6-O-sulfotransferase 2 | HS6ST2 | 2.7 | 1.9 | 2.3 |
| 1555216_a_at | hypothetical protein LOC645722 | LOC645722 | 3.5 | 1.5 | 2.3 |
| 1555889_a_at | cartilage associated protein | CRTAP | 2.8 | 1.9 | 2.3 |
| 1558173_a_at | leucine zipper protein 1 | LUZP1 | 2.6 | 2.0 | 2.3 |
| 1559101_at | FYN oncogene related to SRC, FGR, YES | FYN | 2.3 | 2.4 | 2.3 |
| 1560434_x_at | clathrin, light chain A | CLTA | 3.3 | 1.7 | 2.3 |
| 1566191_at | Suppressor of zeste 12 homolog (Drosophila) | SUZ12 | 2.1 | 2.4 | 2.3 |
| 200022_at | ribosomal protein L18 | RPL18 | 2.4 | 2.1 | 2.3 |
| 200024_at | ribosomal protein S5 | RPS5 | 2.4 | 2.3 | 2.3 |
| 200766_at | cathepsin D | CTSD | 2.3 | 2.3 | 2.3 |
| 201088_at | karyopherin alpha 2 (RAG cohort 1, importin alpha 1) | KPNA2 | 1.8 | 3.0 | 2.3 |
| 201561_s_at | calsyntenin 1 | CLSTN1 | 3.1 | 1.7 | 2.3 |
| 201764_at | transmembrane protein 106C | TMEM106C | 1.9 | 2.8 | 2.3 |
| 201765_s_at | hexosaminidase A (alpha polypeptide) | HEXA | 2.5 | 2.1 | 2.3 |
| 202035_s_at | secreted frizzled-related protein 1 | SFRP1 | 3.0 | 1.8 | 2.3 |
| 202177_at | growth arrest-specific 6 | GAS6 | 2.9 | 1.9 | 2.3 |
| 202709_at | fibromodulin | FMOD | 3.6 | 1.4 | 2.3 |
| 203259_s_at | HD domain containing 2 | HDDC2 | 2.5 | 2.0 | 2.3 |
| 203315_at | NCK adaptor protein 2 | NCK2 | 3.2 | 1.6 | 2.3 |
| 203466_at | MpV17 mitochondrial inner membrane protein | MPV17 | 2.3 | 2.4 | 2.3 |
| 203927_at | nuclear factor of kappa light polypeptide gene enhancer in B-cells inhibitor, epsilon | NFKBIE | 2.7 | 2.0 | 2.3 |
| 204105_s_at | neuronal cell adhesion molecule | NRCAM | 2.6 | 2.1 | 2.3 |
| 204396_s_at | G protein-coupled receptor kinase 5 | GRK5 | 3.0 | 1.7 | 2.3 |
| 204411_at | kinesin family member 21B | KIF21B | 2.2 | 2.4 | 2.3 |
| 204417_at | galactosylceramidase | GALC | 3.0 | 1.8 | 2.3 |
| 204677_at | cadherin 5, type 2 (vascular endothelium) | CDH5 | 2.9 | 1.9 | 2.3 |
| 205291_at | interleukin 2 receptor, beta | IL2RB | 2.4 | 2.2 | 2.3 |
| 207455_at | purinergic receptor P2Y, G-protein coupled, 1 | P2RY1 | 2.9 | 1.8 | 2.3 |
| 207943_x_at | pleiomorphic adenoma gene-like 1 | PLAGL1 | 3.1 | 1.8 | 2.3 |
| 208475_at | FERM domain containing 4A | FRMD4A | 2.9 | 1.8 | 2.3 |
| 208610_s_at | serine/arginine repetitive matrix 2 | SRRM2 | 2.5 | 2.1 | 2.3 |
| 208736_at | actin related protein 2/3 complex, subunit 3, 21kDa | ARPC3 | 2.5 | 2.2 | 2.3 |
| 210606_x_at | killer cell lectin-like receptor subfamily D, member 1 | KLRD1 | 2.3 | 2.3 | 2.3 |
| 210613_s_at | synaptogyrin 1 | SYNGR1 | 3.3 | 1.6 | 2.3 |
| 211506_s_at | interleukin 8 | IL8 | 2.6 | 2.0 | 2.3 |
| 211833_s_at | BCL2-associated X protein | BAX | 2.5 | 2.1 | 2.3 |
| 212813_at | junctional adhesion molecule 3 | JAM3 | 3.0 | 1.7 | 2.3 |
| 213311_s_at | transcription factor 25 (basic helix-loop-helix) | TCF25 | 2.6 | 2.1 | 2.3 |
| 213418_at | heat shock 70kDa protein 6 (HSP70B') | HSPA6 | 2.1 | 2.5 | 2.3 |
| 214091_s_at | glutathione peroxidase 3 (plasma) | GPX3 | 2.1 | 2.5 | 2.3 |
| 215343_at | coiled-coil domain containing 88C | CCDC88C | 2.4 | 2.1 | 2.3 |
| 218687_s_at | mucin 13, cell surface associated | MUC13 | 1.5 | 3.5 | 2.3 |
| 218824_at | PNMA-like 1 | PNMAL1 | 3.6 | 1.4 | 2.3 |
| 219215_s_at | solute carrier family 39 (zinc transporter), member 4 | SLC39A4 | 2.5 | 2.1 | 2.3 |
| 219315_s_at | transmembrane protein 204 | TMEM204 | 3.0 | 1.8 | 2.3 |
| 219583_s_at | spermatogenesis associated 7 | SPATA7 | 2.8 | 1.9 | 2.3 |
| 219762_s_at | ribosomal protein L36 | RPL36 | 2.4 | 2.2 | 2.3 |
| 221191_at | stromal antigen 3-like 1 | STAG3L1 | 2.3 | 2.4 | 2.3 |
| 221690_s_at | NLR family, pyrin domain containing 2 | NLRP2 | 2.1 | 2.5 | 2.3 |
| 223340_at | atlastin GTPase 1 | ATL1 | 3.3 | 1.6 | 2.3 |
| 223494_at | meningioma expressed antigen 5 (hyaluronidase) | MGEA5 | 2.7 | 2.0 | 2.3 |
| 224972_at | reactive oxygen species modulator 1 | ROMO1 | 2.2 | 2.4 | 2.3 |
| 225177_at | RAB11 family interacting protein 1 (class I) | RAB11FIP1 | 2.7 | 2.0 | 2.3 |
| 225295_at | solute carrier family 39 (zinc transporter), member 10 | SLC39A10 | 2.7 | 2.0 | 2.3 |
| 225507_at | splicing factor, arginine/serine-rich 18 | SFRS18 | 2.8 | 1.8 | 2.3 |
| 225582_at | inositol 1,4,5-triphosphate receptor interacting protein | ITPRIP | 2.2 | 2.5 | 2.3 |
| 226301_at | chromosome 6 open reading frame 192 | C6orf192 | 2.8 | 2.0 | 2.3 |
| 226439_s_at | neurobeachin | NBEA | 3.2 | 1.7 | 2.3 |
| 227997_at | interleukin 17 receptor D | IL17RD | 3.0 | 1.7 | 2.3 |
| 228049_x_at | hypothetical LOC100507303 | LOC100507303 | 1.8 | 2.8 | 2.3 |
| 228461_at | SH3 domain containing ring finger 3 | SH3RF3 | 3.1 | 1.7 | 2.3 |
| 228606_at | Tctex1 domain containing 2 | TCTEX1D2 | 2.5 | 2.1 | 2.3 |
| 228624_at | transmembrane protein 144 | TMEM144 | 2.6 | 2.0 | 2.3 |
| 229029_at | calcium/calmodulin-dependent protein kinase IV | CAMK4 | 2.6 | 2.0 | 2.3 |
| 230109_at | phosphodiesterase 7B | PDE7B | 3.4 | 1.6 | 2.3 |
| 232094_at | chromosome 15 open reading frame 29 | C15orf29 | 2.3 | 2.3 | 2.3 |
| 232676_x_at | myelin expression factor 2 | MYEF2 | 2.8 | 1.9 | 2.3 |
| 233461_x_at | zinc finger protein 226 | ZNF226 | 3.1 | 1.7 | 2.3 |
| 235728_at | zinc finger protein 3 homolog (mouse) | ZFP3 | 2.9 | 1.8 | 2.3 |
| 238332_at | ankyrin repeat domain 29 | ANKRD29 | 2.1 | 2.5 | 2.3 |
| 238654_at | V-set and immunoglobulin domain containing 10 like | VSIG10L | 1.8 | 2.9 | 2.3 |
| 34726_at | calcium channel, voltage-dependent, beta 3 subunit | CACNB3 | 2.8 | 1.8 | 2.3 |
| 47550_at | leucine zipper, putative tumor suppressor 1 | LZTS1 | 2.5 | 2.2 | 2.3 |
| 1552621_at | polymerase (RNA) II (DNA directed) polypeptide J2 | POLR2J2 | 2.3 | 2.1 | 2.2 |
| 1556425_a_at | hypothetical protein LOC284219 | LOC284219 | 2.8 | 1.8 | 2.2 |
| 1559078_at | B-cell CLL/lymphoma 11A (zinc finger protein) | BCL11A | 2.7 | 1.8 | 2.2 |
| 1568619_s_at | inositol 1,4,5-triphosphate receptor interacting protein-like 2 | ITPRIPL2 | 2.5 | 2.0 | 2.2 |
| 200634_at | profilin 1 | PFN1 | 1.9 | 2.4 | 2.2 |
| 200663_at | CD63 molecule | CD63 | 2.4 | 2.1 | 2.2 |
| 200788_s_at | phosphoprotein enriched in astrocytes 15 | PEA15 | 2.2 | 2.1 | 2.2 |
| 200866_s_at | prosaposin | PSAP | 2.3 | 2.1 | 2.2 |
| 200972_at | tetraspanin 3 | TSPAN3 | 2.6 | 1.9 | 2.2 |
| 201506_at | transforming growth factor, beta-induced, 68kDa | TGFBI | 2.1 | 2.4 | 2.2 |
| 201892_s_at | IMP (inosine 5'-monophosphate) dehydrogenase 2 | IMPDH2 | 1.9 | 2.4 | 2.2 |
| 201926_s_at | CD55 molecule, decay accelerating factor for complement (Cromer blood group) | CD55 | 2.5 | 1.8 | 2.2 |
| 203196_at | ATP-binding cassette, sub-family C (CFTR/MRP), member 4 | ABCC4 | 2.9 | 1.7 | 2.2 |
| 203397_s_at | UDP-N-acetyl-alpha-D-galactosamine:polypeptide N-acetylgalactosaminyltransferase 3 (GalNAc-T3) | GALNT3 | 2.8 | 1.7 | 2.2 |
| 203630_s_at | component of oligomeric golgi complex 5 | COG5 | 2.2 | 2.3 | 2.2 |
| 204073_s_at | chromosome 11 open reading frame 9 | C11orf9 | 2.4 | 2.1 | 2.2 |
| 204613_at | phospholipase C, gamma 2 (phosphatidylinositol-specific) | PLCG2 | 2.6 | 1.9 | 2.2 |
| 204964_s_at | sarcospan (Kras oncogene-associated gene) | SSPN | 2.6 | 1.8 | 2.2 |
| 205128_x_at | prostaglandin-endoperoxide synthase 1 (prostaglandin G/H synthase and cyclooxygenase) | PTGS1 | 2.9 | 1.7 | 2.2 |
| 205866_at | ficolin (collagen/fibrinogen domain containing) 3 (Hakata antigen) | FCN3 | 2.5 | 1.9 | 2.2 |
| 206090_s_at | disrupted in schizophrenia 1  TSNAX-DISC1 gene | DISC1  TSNAX-DISC1 | 2.6 | 1.8 | 2.2 |
| 206181_at | signaling lymphocytic activation molecule family member 1 | SLAMF1 | 2.5 | 2.0 | 2.2 |
| 206829_x_at | zinc finger protein 430 | ZNF430 | 2.5 | 1.9 | 2.2 |
| 207394_at | zinc finger protein 137, pseudogene | ZNF137P | 2.9 | 1.7 | 2.2 |
| 207992_s_at | adenosine monophosphate deaminase 3 | AMPD3 | 3.0 | 1.6 | 2.2 |
| 208727_s_at | cell division cycle 42 (GTP binding protein, 25kDa) | CDC42 | 2.2 | 2.3 | 2.2 |
| 208974_x_at | karyopherin (importin) beta 1 | KPNB1 | 2.1 | 2.5 | 2.2 |
| 209043_at | 3'-phosphoadenosine 5'-phosphosulfate synthase 1 | PAPSS1 | 2.8 | 1.8 | 2.2 |
| 209197_at | synaptotagmin XI | SYT11 | 2.6 | 1.8 | 2.2 |
| 209219_at | RD RNA binding protein | RDBP | 2.2 | 2.2 | 2.2 |
| 209270_at | laminin, beta 3 | LAMB3 | 2.1 | 2.2 | 2.2 |
| 209596_at | matrix-remodelling associated 5 | MXRA5 | 3.0 | 1.7 | 2.2 |
| 209787_s_at | high mobility group nucleosomal binding domain 4 | HMGN4 | 2.4 | 2.0 | 2.2 |
| 209834_at | carbohydrate (chondroitin 6) sulfotransferase 3 | CHST3 | 2.8 | 1.8 | 2.2 |
| 210873_x_at | apolipoprotein B mRNA editing enzyme, catalytic polypeptide-like 3A | APOBEC3A | 2.2 | 2.2 | 2.2 |
| 212055_at | chromosome 18 open reading frame 10 | C18orf10 | 2.1 | 2.2 | 2.2 |
| 212307_s_at | O-linked N-acetylglucosamine (GlcNAc) transferase (UDP-N-acetylglucosamine:polypeptide-N-acetylglucosaminyl transferase) | OGT | 2.5 | 2.0 | 2.2 |
| 212573_at | endonuclease domain containing 1 | ENDOD1 | 2.6 | 1.8 | 2.2 |
| 212829_at | phosphatidylinositol-5-phosphate 4-kinase, type II, alpha | PIP4K2A | 2.5 | 1.9 | 2.2 |
| 213526_s_at | lin-37 homolog (C. elegans) | LIN37 | 2.2 | 2.3 | 2.2 |
| 215127_s_at | RNA binding motif, single stranded interacting protein 1 | RBMS1 | 2.4 | 1.9 | 2.2 |
| 215952_s_at | ornithine decarboxylase antizyme 1 | OAZ1 | 2.3 | 2.0 | 2.2 |
| 218589_at | lysophosphatidic acid receptor 6 | LPAR6 | 2.7 | 1.7 | 2.2 |
| 219316_s_at | feline leukemia virus subgroup C cellular receptor family, member 2 | FLVCR2 | 2.0 | 2.4 | 2.2 |
| 219412_at | RAB38, member RAS oncogene family | RAB38 | 2.6 | 1.8 | 2.2 |
| 219508_at | glucosaminyl (N-acetyl) transferase 3, mucin type | GCNT3 | 1.5 | 3.2 | 2.2 |
| 219679_s_at | WW domain containing adaptor with coiled-coil | WAC | 2.4 | 1.9 | 2.2 |
| 220145_at | microtubule-associated protein 9 | MAP9 | 3.0 | 1.6 | 2.2 |
| 222746_s_at | B-box and SPRY domain containing | BSPRY | 2.7 | 1.8 | 2.2 |
| 223464_at | oxysterol binding protein-like 5 | OSBPL5 | 2.8 | 1.8 | 2.2 |
| 224574_at | chromosome 17 open reading frame 49 | C17orf49 | 2.3 | 2.1 | 2.2 |
| 224942_at | pregnancy-associated plasma protein A, pappalysin 1 | PAPPA | 2.6 | 1.9 | 2.2 |
| 225639_at | src kinase associated phosphoprotein 2 | SKAP2 | 2.6 | 1.8 | 2.2 |
| 225913_at | NKF3 kinase family member | SGK269 | 2.7 | 1.8 | 2.2 |
| 226287_at | coiled-coil domain containing 34 | CCDC34 | 1.9 | 2.4 | 2.2 |
| 226575_at | zinc finger protein 462 | ZNF462 | 3.1 | 1.6 | 2.2 |
| 226632_at | cytoglobin | CYGB | 3.0 | 1.6 | 2.2 |
| 226641_at | ankyrin repeat domain 44 | ANKRD44 | 2.3 | 2.0 | 2.2 |
| 226751_at | cannabinoid receptor interacting protein 1 | CNRIP1 | 2.6 | 1.8 | 2.2 |
| 227985_at | hypothetical LOC100506098 | LOC100506098 | 2.8 | 1.8 | 2.2 |
| 228067_at | chromosome 2 open reading frame 55 | C2orf55 | 2.7 | 1.8 | 2.2 |
| 228158_at | lymphocyte-specific protein 1 pseudogene  lymphocyte-specific protein 1 pseudogene | LOC645166 LOC654342 | 2.7 | 1.8 | 2.2 |
| 229699_at | hypothetical LOC100129550 | LOC100129550 | 2.8 | 1.8 | 2.2 |
| 229700_at | zinc finger protein 738 | ZNF738 | 2.4 | 2.0 | 2.2 |
| 230093_at | radial spoke head 1 homolog (Chlamydomonas) | RSPH1 | 2.3 | 2.2 | 2.2 |
| 230480_at | piwi-like 4 (Drosophila) | PIWIL4 | 2.9 | 1.6 | 2.2 |
| 231292_at | EP300 interacting inhibitor of differentiation 3 | EID3 | 2.0 | 2.5 | 2.2 |
| 232412_at | F-box and leucine-rich repeat protein 20 | FBXL20 | 2.6 | 1.8 | 2.2 |
| 232940_s_at | myeloid/lymphoid or mixed-lineage leukemia 3 | MLL3 | 2.5 | 1.9 | 2.2 |
| 235014_at | hypothetical LOC147727 | LOC147727 | 2.2 | 2.1 | 2.2 |
| 235167_at | hypothetical LOC100190986 | LOC100190986 | 2.4 | 2.0 | 2.2 |
| 235461_at | tet oncogene family member 2 | TET2 | 2.6 | 1.8 | 2.2 |
| 235484_at | protein prenyltransferase alpha subunit repeat containing 1 | PTAR1 | 2.8 | 1.8 | 2.2 |
| 238706_at | PAP associated domain containing 4 | PAPD4 | 2.9 | 1.7 | 2.2 |
| 60815_at | polymerase (RNA) II (DNA directed) polypeptide J4, pseudogene | POLR2J4 | 2.5 | 2.0 | 2.2 |
| 1552386_at | GRB2-binding adaptor protein, transmembrane | GAPT | 2.3 | 1.9 | 2.1 |
| 1552611_a_at | Janus kinase 1 | JAK1 | 2.4 | 1.9 | 2.1 |
| 1559413_at | t-complex 11 (mouse)-like 2 | TCP11L2 | 2.7 | 1.6 | 2.1 |
| 200001_at | calpain, small subunit 1 | CAPNS1 | 2.1 | 2.1 | 2.1 |
| 200081_s_at | ribosomal protein S6 | RPS6 | 2.2 | 2.0 | 2.1 |
| 200885_at | ras homolog gene family, member C | RHOC | 1.9 | 2.2 | 2.1 |
| 200940_s_at | arginine-glutamic acid dipeptide (RE) repeats | RERE | 2.4 | 1.8 | 2.1 |
| 201040_at | guanine nucleotide binding protein (G protein), alpha inhibiting activity polypeptide 2 | GNAI2 | 2.1 | 2.0 | 2.1 |
| 202098_s_at | protein arginine methyltransferase 2 | PRMT2 | 2.7 | 1.7 | 2.1 |
| 202307_s_at | transporter 1, ATP-binding cassette, sub-family B (MDR/TAP) | TAP1 | 2.1 | 2.2 | 2.1 |
| 202421_at | immunoglobulin superfamily, member 3 | IGSF3 | 2.5 | 1.7 | 2.1 |
| 203211_s_at | myotubularin related protein 2 | MTMR2 | 2.3 | 1.9 | 2.1 |
| 203358_s_at | enhancer of zeste homolog 2 (Drosophila) | EZH2 | 1.8 | 2.3 | 2.1 |
| 203592_s_at | follistatin-like 3 (secreted glycoprotein) | FSTL3 | 2.4 | 1.8 | 2.1 |
| 203856_at | vaccinia related kinase 1 | VRK1 | 2.0 | 2.2 | 2.1 |
| 204057_at | interferon regulatory factor 8 | IRF8 | 2.5 | 1.7 | 2.1 |
| 204174_at | arachidonate 5-lipoxygenase-activating protein | ALOX5AP | 2.4 | 1.8 | 2.1 |
| 204641_at | NIMA (never in mitosis gene a)-related kinase 2 | NEK2 | 1.4 | 3.1 | 2.1 |
| 204680_s_at | Rap guanine nucleotide exchange factor (GEF) 5 | RAPGEF5 | 2.3 | 1.9 | 2.1 |
| 205010_at | guanine nucleotide binding protein-like 3 (nucleolar)-like | GNL3L | 2.0 | 2.1 | 2.1 |
| 205080_at | retinoic acid receptor, beta | RARB | 2.8 | 1.6 | 2.1 |
| 209132_s_at | COMM domain containing 4 | COMMD4 | 2.0 | 2.2 | 2.1 |
| 209258_s_at | structural maintenance of chromosomes 3 | SMC3 | 2.4 | 1.8 | 2.1 |
| 209422_at | PHD finger protein 20 | PHF20 | 2.3 | 1.9 | 2.1 |
| 209969_s_at | signal transducer and activator of transcription 1, 91kDa | STAT1 | 2.2 | 2.0 | 2.1 |
| 210980_s_at | N-acylsphingosine amidohydrolase (acid ceramidase) 1 | ASAH1 | 2.3 | 1.9 | 2.1 |
| 211005_at | linker for activation of T cells  spinster homolog 1 (Drosophila) | LAT  SPNS1 | 2.4 | 1.9 | 2.1 |
| 212256_at | UDP-N-acetyl-alpha-D-galactosamine:polypeptide N-acetylgalactosaminyltransferase 10 (GalNAc-T10) | GALNT10 | 2.2 | 1.9 | 2.1 |
| 212958_x_at | peptidylglycine alpha-amidating monooxygenase | PAM | 2.5 | 1.7 | 2.1 |
| 213135_at | T-cell lymphoma invasion and metastasis 1 | TIAM1 | 2.9 | 1.5 | 2.1 |
| 213358_at | KIAA0802 | KIAA0802 | 2.7 | 1.7 | 2.1 |
| 214783_s_at | annexin A11 | ANXA11 | 2.6 | 1.7 | 2.1 |
| 215011_at | small nucleolar RNA host gene 3 (non-protein coding) | SNHG3 | 1.4 | 3.2 | 2.1 |
| 216194_s_at | tubulin folding cofactor B | TBCB | 2.2 | 2.0 | 2.1 |
| 218350_s_at | geminin, DNA replication inhibitor | GMNN | 2.0 | 2.2 | 2.1 |
| 218764_at | protein kinase C, eta | PRKCH | 2.5 | 1.7 | 2.1 |
| 218815_s_at | transmembrane protein 51 | TMEM51 | 2.8 | 1.6 | 2.1 |
| 219694_at | family with sequence similarity 105, member A | FAM105A | 2.8 | 1.6 | 2.1 |
| 219985_at | heparan sulfate (glucosamine) 3-O-sulfotransferase 3A1 | HS3ST3A1 | 2.3 | 2.0 | 2.1 |
| 220146_at | toll-like receptor 7 | TLR7 | 2.8 | 1.6 | 2.1 |
| 220468_at | ADP-ribosylation factor-like 14 | ARL14 | 1.8 | 2.5 | 2.1 |
| 220694_at | ASAP1 intronic transcript (non-protein coding) | ASAP1-IT | 2.2 | 2.1 | 2.1 |
| 222034_at | guanine nucleotide binding protein (G protein), beta polypeptide 2-like 1 | GNB2L1 | 1.9 | 2.2 | 2.1 |
| 222387_s_at | vacuolar protein sorting 35 homolog (S. cerevisiae) | VPS35 | 2.2 | 2.0 | 2.1 |
| 224227_s_at | B double prime 1, subunit of RNA polymerase III transcription initiation factor IIIB | BDP1 | 2.2 | 2.0 | 2.1 |
| 224833_at | v-ets erythroblastosis virus E26 oncogene homolog 1 (avian) | ETS1 | 2.3 | 1.9 | 2.1 |
| 224908_s_at | tubulin tyrosine ligase | TTL | 1.9 | 2.4 | 2.1 |
| 224927_at | KIAA1949 | KIAA1949 | 2.2 | 1.9 | 2.1 |
| 224959_at | solute carrier family 26 (sulfate transporter), member 2 | SLC26A2 | 2.3 | 1.8 | 2.1 |
| 225228_at | DNA-damage regulated autophagy modulator 2 | DRAM2 | 2.5 | 1.8 | 2.1 |
| 225626_at | phosphoprotein associated with glycosphingolipid microdomains 1 | PAG1 | 2.3 | 2.0 | 2.1 |
| 225921_at | ninein (GSK3B interacting protein) | NIN | 2.3 | 2.0 | 2.1 |
| 226055_at | arrestin domain containing 2 | ARRDC2 | 2.6 | 1.8 | 2.1 |
| 226080_at | slingshot homolog 2 (Drosophila) | SSH2 | 2.4 | 1.9 | 2.1 |
| 227074_at | hypothetical LOC100131564 | LOC100131564 | 2.4 | 1.9 | 2.1 |
| 227484_at | SLIT-ROBO Rho GTPase activating protein 1 | SRGAP1 | 2.5 | 1.7 | 2.1 |
| 229399_at | chromosome 10 open reading frame 118 | C10orf118 | 2.7 | 1.7 | 2.1 |
| 230100_x_at | p21 protein (Cdc42/Rac)-activated kinase 1 | PAK1 | 2.6 | 1.7 | 2.1 |
| 230466_s_at | --- | --- | 2.4 | 1.9 | 2.1 |
| 233565_s_at | syndecan binding protein (syntenin) 2 | SDCBP2 | 1.4 | 3.1 | 2.1 |
| 235721_at | deltex homolog 3 (Drosophila) | DTX3 | 2.9 | 1.5 | 2.1 |
| 235871_at | lipase, member H | LIPH | 2.1 | 2.2 | 2.1 |
| 236561_at | --- | --- | 2.1 | 2.0 | 2.1 |
| 236562_at | zinc finger protein 439 | ZNF439 | 2.9 | 1.5 | 2.1 |
| 238350_at | ubinuclein 2 | UBN2 | 2.3 | 2.0 | 2.1 |
| 240257_at | synaptojanin 2 | SYNJ2 | 2.5 | 1.8 | 2.1 |
| 240260_at | --- | --- | 1.9 | 2.4 | 2.1 |
| 243140_at | Actin, alpha 2, smooth muscle, aorta | ACTA2 | 2.0 | 2.3 | 2.1 |
| 243835_at | zinc finger, DHHC-type containing 21 | ZDHHC21 | 2.4 | 1.8 | 2.1 |
| 1552797_s_at | prominin 2 | PROM2 | 2.7 | 1.5 | 2.0 |
| 1556698_a_at | GPRIN family member 3 | GPRIN3 | 1.9 | 2.1 | 2.0 |
| 200823_x_at | ribosomal protein L29 | RPL29 | 2.0 | 2.0 | 2.0 |
| 202471_s_at | isocitrate dehydrogenase 3 (NAD+) gamma | IDH3G | 2.0 | 2.1 | 2.0 |
| 202986_at | aryl-hydrocarbon receptor nuclear translocator 2 | ARNT2 | 3.0 | 1.3 | 2.0 |
| 203892_at | WAP four-disulfide core domain 2 | WFDC2 | 2.8 | 1.4 | 2.0 |
| 204454_at | leucine zipper, down-regulated in cancer 1 | LDOC1 | 2.9 | 1.4 | 2.0 |
| 204562_at | interferon regulatory factor 4 | IRF4 | 2.9 | 1.4 | 2.0 |
| 204804_at | tripartite motif-containing 21 | TRIM21 | 2.2 | 1.9 | 2.0 |
| 206565_x_at | glucuronidase, beta pseudogene | SMA4 | 2.3 | 1.8 | 2.0 |
| 207840_at | CD160 molecule | CD160 | 2.7 | 1.5 | 2.0 |
| 208885_at | lymphocyte cytosolic protein 1 (L-plastin) | LCP1 | 2.1 | 2.0 | 2.0 |
| 210410_s_at | chromosome 6 open reading frame 26  mutS homolog 5 (E. coli) | C6orf26  MSH5 | 2.0 | 2.0 | 2.0 |
| 211163_s_at | tumor necrosis factor receptor superfamily, member 10c, decoy without an intracellular domain | TNFRSF10C | 2.5 | 1.7 | 2.0 |
| 211555_s_at | guanylate cyclase 1, soluble, beta 3 | GUCY1B3 | 2.6 | 1.6 | 2.0 |
| 212451_at | SECIS binding protein 2-like | SECISBP2L | 2.4 | 1.7 | 2.0 |
| 212906_at | GRAM domain containing 1B | GRAMD1B | 2.4 | 1.6 | 2.0 |
| 213316_at | KIAA1462 | KIAA1462 | 2.7 | 1.5 | 2.0 |
| 215247_at | LIM and senescent cell antigen-like domains 3 | LIMS3 | 2.3 | 1.8 | 2.0 |
| 217497_at | thymidine phosphorylase | TYMP | 1.8 | 2.3 | 2.0 |
| 217593_at | zinc finger and SCAN domain containing 18 | ZSCAN18 | 2.5 | 1.7 | 2.0 |
| 217807_s_at | glioma tumor suppressor candidate region gene 2 | GLTSCR2 | 2.3 | 1.8 | 2.0 |
| 219341_at | ceroid-lipofuscinosis, neuronal 8 (epilepsy, progressive with mental retardation) | CLN8 | 2.0 | 2.0 | 2.0 |
| 219558_at | ATPase type 13A3 | ATP13A3 | 2.0 | 2.0 | 2.0 |
| 219667_s_at | B-cell scaffold protein with ankyrin repeats 1 | BANK1 | 2.3 | 1.7 | 2.0 |
| 219892_at | transmembrane 6 superfamily member 1 | TM6SF1 | 2.3 | 1.8 | 2.0 |
| 220755_s_at | chromosome 6 open reading frame 48 | C6orf48 | 2.5 | 1.6 | 2.0 |
| 222233_s_at | DNA cross-link repair 1C | DCLRE1C | 2.4 | 1.8 | 2.0 |
| 222257_s_at | angiotensin I converting enzyme (peptidyl-dipeptidase A) 2 | ACE2 | 1.4 | 2.9 | 2.0 |
| 222810_s_at | RAS protein activator like 2 | RASAL2 | 2.3 | 1.8 | 2.0 |
| 223322_at | Ras association (RalGDS/AF-6) domain family member 5 | RASSF5 | 2.3 | 1.8 | 2.0 |
| 224573_at | ribonuclease, RNase K | RNASEK | 2.0 | 2.0 | 2.0 |
| 224631_at | zinc finger protein 91 homolog (mouse) | ZFP91 | 2.2 | 1.9 | 2.0 |
| 224772_at | neuron navigator 1 | NAV1 | 2.9 | 1.4 | 2.0 |
| 227946_at | oxysterol binding protein-like 7 | OSBPL7 | 2.4 | 1.7 | 2.0 |
| 228310_at | enabled homolog (Drosophila) | ENAH | 2.3 | 1.8 | 2.0 |
| 229872_s_at | hypothetical LOC100132999 | LOC100132999 | 2.7 | 1.6 | 2.0 |
| 230418_s_at | UDP-N-acetyl-alpha-D-galactosamine:polypeptide N-acetylgalactosaminyltransferase-like 1 | GALNTL1 | 2.3 | 1.7 | 2.0 |
| 230930_at | hypothetical protein LOC338620 | LOC338620 | 2.1 | 1.9 | 2.0 |
| 232001_at | hypothetical LOC439949 | LOC439949 | 2.1 | 2.0 | 2.0 |
| 236808_at | FGFR1 oncogene partner 2 | FGFR1OP2 | 2.3 | 1.8 | 2.0 |
| 243256_at | MAP kinase interacting serine/threonine kinase 1 | MKNK1 | 2.6 | 1.6 | 2.0 |

* Transcript name based on probe target sequence. MHN denotes massive hepatic necrosis. SHN denotes submassive hepatic necrosis. LD denotes liver donors. ALF denotes acute liver failure.
