# Supplementary material for: Liver Regeneration Signature in Hepatitis B Virus (HBV)-Associated Acute Liver Failure Identified by Gene Expression Profiling
Source: PLoS One. 2012 Nov 21;7(11):e49611. doi: 10.1371/journal.pone.0049611 (PMC3504149; doi:10.1371/journal.pone.0049611)
Supplement: Table S2 — Down-regulated Transcripts in HBV-Associated Acute Liver Failure. (DOCX) [file pone.0049611.s002.docx]

| **Table S2. Down-regulated Transcripts in HBV-Associated Acute Liver Failure** | | | | | |
| --- | --- | --- | --- | --- | --- |
|  |  |  |  |  |  |
|  |  |  | **Fold Change** | | |
| **Affymetrix Probe ID** | **Transcript Name** | **Gene Symbol** | **MHN/LD** | **SHN/LD** | **All ALF Cases/LD** |
| 206727_at | complement component 9 | C9 | -1006.0 | -20.0 | -141.7 |
| 208607_s_at | serum amyloid A1  serum amyloid A2 | SAA1  SAA2 | -524.9 | -28.7 | -122.8 |
| 207874_s_at | complement factor H-related 4 | CFHR4 | -329.4 | -24.4 | -89.6 |
| 207218_at | coagulation factor IX | F9 | -882.6 | -7.5 | -81.4 |
| 1554459_s_at | complement factor H-related 3 | CFHR3 | -158.2 | -35.5 | -74.9 |
| 205700_at | hydroxysteroid (17-beta) dehydrogenase 6 homolog (mouse) | HSD17B6 | -339.0 | -12.8 | -65.9 |
| 207262_at | apolipoprotein F | APOF | -196.0 | -19.2 | -61.4 |
| 205040_at | orosomucoid 1 | ORM1 | -338.6 | -10.6 | -60.0 |
| 229596_at | amidohydrolase domain containing 1 | AMDHD1 | -160.9 | -15.1 | -49.2 |
| 207584_at | lipoprotein, Lp(a) | LPA | -99.9 | -23.9 | -48.8 |
| 208088_s_at | complement factor H-related 5 | CFHR5 | -218.9 | -9.2 | -44.8 |
| 210013_at | hemopexin | HPX | -167.9 | -9.1 | -39.0 |
| 220786_s_at | solute carrier family 38, member 4 | SLC38A4 | -208.9 | -6.6 | -37.1 |
| 227253_at | ceruloplasmin (ferroxidase) | CP | -183.4 | -7.4 | -36.8 |
| 210328_at | glycine N-methyltransferase | GNMT | -41.5 | -31.6 | -36.2 |
| 228844_at | solute carrier family 13 (sodium-dependent citrate transporter), member 5 | SLC13A5 | -139.5 | -9.4 | -36.1 |
| 206305_s_at | complement component 8, alpha polypeptide | C8A | -185.4 | -6.7 | -35.4 |
| 220626_at | serpin peptidase inhibitor, clade A (alpha-1 antiproteinase, antitrypsin), member 10 | SERPINA10 | -232.2 | -5.4 | -35.4 |
| 206979_at | complement component 8, beta polypeptide | C8B | -114.5 | -10.8 | -35.2 |
| 205649_s_at | fibrinogen alpha chain | FGA | -464.3 | -2.7 | -35.1 |
| 210215_at | transferrin receptor 2 | TFR2 | -138.5 | -8.5 | -34.3 |
| 209978_s_at | lipoprotein, Lp(a)  plasminogen | LPA  PLG | -231.4 | -4.8 | -33.3 |
| 205753_at | C-reactive protein, pentraxin-related | CRP | -164.0 | -6.2 | -31.9 |
| 207256_at | mannose-binding lectin (protein C) 2, soluble (opsonic defect) | MBL2 | -261.9 | -3.9 | -31.8 |
| 216238_s_at | fibrinogen beta chain | FGB | -400.9 | -2.5 | -31.5 |
| 204154_at | cysteine dioxygenase, type I | CDO1 | -150.2 | -6.4 | -31.1 |
| 1554491_a_at | serpin peptidase inhibitor, clade C (antithrombin), member 1 | SERPINC1 | -128.5 | -7.5 | -31.0 |
| 206345_s_at | paraoxonase 1 | PON1 | -202.5 | -4.7 | -30.9 |
| 241914_s_at | acyl-CoA synthetase medium-chain family member 2A  acyl-CoA synthetase medium-chain family member 2B | ACSM2A  ACSM2B | -123.7 | -7.7 | -30.8 |
| 244723_at | acyl-CoA synthetase medium-chain family member 2A | ACSM2A | -53.6 | -17.3 | -30.4 |
| 217512_at | kininogen 1 | KNG1 | -143.9 | -6.2 | -29.8 |
| 205083_at | aldehyde oxidase 1 | AOX1 | -140.0 | -6.3 | -29.6 |
| 227794_at | glycine-N-acyltransferase-like 1 | GLYATL1 | -172.2 | -4.9 | -29.1 |
| 209631_s_at | G protein-coupled receptor 37 (endothelin receptor type B-like) | GPR37 | -35.5 | -22.9 | -28.5 |
| 206515_at | cytochrome P450, family 4, subfamily F, polypeptide 3 | CYP4F3 | -108.0 | -7.1 | -27.8 |
| 219803_at | angiopoietin-like 3 | ANGPTL3 | -225.5 | -3.4 | -27.7 |
| 207097_s_at | solute carrier family 17 (sodium phosphate), member 2 | SLC17A2 | -134.8 | -5.6 | -27.6 |
| 207096_at | serum amyloid A4, constitutive | SAA4 | -215.8 | -3.5 | -27.5 |
| 205576_at | serpin peptidase inhibitor, clade D (heparin cofactor), member 1 | SERPIND1 | -185.9 | -3.9 | -27.1 |
| 229819_at | alpha-1-B glycoprotein | A1BG | -103.4 | -7.0 | -26.9 |
| 214461_at | lipopolysaccharide binding protein | LBP | -244.8 | -2.8 | -26.3 |
| 209309_at | alpha-2-glycoprotein 1, zinc-binding | AZGP1 | -88.3 | -7.7 | -26.1 |
| 231678_s_at | alcohol dehydrogenase 4 (class II), pi polypeptide | ADH4 | -267.1 | -2.5 | -25.9 |
| 206354_at | solute carrier organic anion transporter family, member 1B3 | SLCO1B3 | -78.5 | -8.1 | -25.3 |
| 235708_at | klotho beta | KLB | -50.2 | -12.6 | -25.1 |
| 220801_s_at | hydroxyacid oxidase 2 (long chain) | HAO2 | -40.3 | -15.5 | -25.0 |
| 207201_s_at | solute carrier family 22 (organic cation transporter), member 1 | SLC22A1 | -109.2 | -5.4 | -24.3 |
| 210587_at | inhibin, beta E | INHBE | -209.2 | -2.8 | -24.3 |
| 207102_at | aldo-keto reductase family 1, member D1 (delta 4-3-ketosteroid-5-beta-reductase) | AKR1D1 | -128.1 | -4.5 | -24.0 |
| 210327_s_at | alanine-glyoxylate aminotransferase | AGXT | -129.7 | -4.3 | -23.7 |
| 205152_at | solute carrier family 6 (neurotransmitter transporter, GABA), member 1 | SLC6A1 | -23.7 | -23.3 | -23.5 |
| 205442_at | microfibrillar-associated protein 3-like | MFAP3L | -40.5 | -13.7 | -23.5 |
| 206177_s_at | arginase, liver | ARG1 | -174.1 | -3.2 | -23.5 |
| 206287_s_at | inter-alpha (globulin) inhibitor H4 (plasma Kallikrein-sensitive glycoprotein)  musculoskeletal, embryonic nuclear protein 1 | ITIH4  MUSTN1 | -86.4 | -6.2 | -23.2 |
| 238752_at | --- | --- | -44.5 | -12.0 | -23.1 |
| 207608_x_at | cytochrome P450, family 1, subfamily A, polypeptide 2 | CYP1A2 | -42.1 | -12.3 | -22.8 |
| 210143_at | annexin A10 | ANXA10 | -46.2 | -10.9 | -22.4 |
| 206386_at | serpin peptidase inhibitor, clade A (alpha-1 antiproteinase, antitrypsin), member 7 | SERPINA7 | -104.1 | -4.8 | -22.3 |
| 207820_at | alcohol dehydrogenase 1A (class I), alpha polypeptide | ADH1A | -122.4 | -4.1 | -22.3 |
| 203914_x_at | hydroxyprostaglandin dehydrogenase 15-(NAD) | HPGD | -47.0 | -10.3 | -22.0 |
| 231398_at | solute carrier family 22 (organic anion transporter), member 7 | SLC22A7 | -141.5 | -3.4 | -22.0 |
| 210233_at | interleukin 1 receptor accessory protein | IL1RAP | -27.2 | -17.6 | -21.9 |
| 205754_at | coagulation factor II (thrombin) | F2 | -104.2 | -4.5 | -21.8 |
| 208471_at | haptoglobin-related protein | HPR | -99.1 | -4.6 | -21.3 |
| 206643_at | histidine ammonia-lyase | HAL | -59.1 | -7.6 | -21.2 |
| 207414_s_at | proprotein convertase subtilisin/kexin type 6 | PCSK6 | -55.3 | -8.2 | -21.2 |
| 228621_at | hemochromatosis type 2 (juvenile) | HFE2 | -60.6 | -7.3 | -21.1 |
| 1556388_a_at | --- | --- | -37.0 | -11.5 | -20.7 |
| 206256_at | carboxypeptidase N, polypeptide 1 | CPN1 | -58.3 | -7.3 | -20.6 |
| 210366_at | solute carrier organic anion transporter family, member 1B1 | SLCO1B1 | -87.2 | -4.9 | -20.6 |
| 206226_at | histidine-rich glycoprotein | HRG | -220.8 | -1.9 | -20.4 |
| 205768_s_at | solute carrier family 27 (fatty acid transporter), member 2 | SLC27A2 | -173.2 | -2.4 | -20.3 |
| 206457_s_at | deiodinase, iodothyronine, type I | DIO1 | -108.9 | -3.6 | -19.8 |
| 208209_s_at | complement component 4 binding protein, beta | C4BPB | -53.2 | -7.1 | -19.5 |
| 209460_at | 4-aminobutyrate aminotransferase | ABAT | -41.6 | -8.6 | -18.9 |
| 229229_at | alanine--glyoxylate aminotransferase 2 | AGXT2 | -60.6 | -5.9 | -18.9 |
| 210888_s_at | inter-alpha (globulin) inhibitor H1 | ITIH1 | -88.5 | -4.0 | -18.7 |
| 232494_at | cytochrome P450, family 8, subfamily B, polypeptide 1 | CYP8B1 | -130.4 | -2.7 | -18.7 |
| 210452_x_at | cytochrome P450, family 4, subfamily F, polypeptide 2 | CYP4F2 | -48.3 | -7.2 | -18.6 |
| 214621_at | glycogen synthase 2 (liver) | GYS2 | -86.2 | -4.0 | -18.6 |
| 220432_s_at | cytochrome P450, family 39, subfamily A, polypeptide 1 | CYP39A1 | -26.9 | -12.6 | -18.4 |
| 214842_s_at | Human serum albumin (ALB) gene, complete cds* | ALB | -67.5 | -4.9 | -18.2 |
| 228648_at | leucine-rich alpha-2-glycoprotein 1 | LRG1 | -57.4 | -5.7 | -18.2 |
| 204607_at | 3-hydroxy-3-methylglutaryl-CoA synthase 2 (mitochondrial) | HMGCS2 | -153.4 | -2.1 | -18.1 |
| 205654_at | complement component 4 binding protein, alpha | C4BPA | -39.5 | -8.3 | -18.1 |
| 229476_s_at | thyroid hormone responsive | THRSP | -45.1 | -7.1 | -18.0 |
| 204284_at | protein phosphatase 1, regulatory (inhibitor) subunit 3C | PPP1R3C | -18.9 | -16.8 | -17.8 |
| 205728_at | odz, odd Oz/ten-m homolog 1(Drosophila) | ODZ1 | -32.3 | -9.9 | -17.8 |
| 206753_at | retinol dehydrogenase 16 (all-trans) | RDH16 | -48.4 | -6.6 | -17.8 |
| 231691_at | complement component 3 precursor pseudogene | C3P1 | -53.9 | -5.5 | -17.3 |
| 205813_s_at | methionine adenosyltransferase I, alpha | MAT1A | -89.1 | -3.3 | -17.2 |
| 213695_at | paraoxonase 3 | PON3 | -35.9 | -8.2 | -17.1 |
| 220148_at | aldehyde dehydrogenase 8 family, member A1 | ALDH8A1 | -54.6 | -5.3 | -17.1 |
| 220383_at | ATP-binding cassette, sub-family G (WHITE), member 5 | ABCG5 | -105.5 | -2.7 | -17.0 |
| 222071_s_at | solute carrier organic anion transporter family, member 4C1 | SLCO4C1 | -17.4 | -16.6 | -17.0 |
| 231790_at | dimethylglycine dehydrogenase | DMGDH | -68.0 | -4.2 | -16.9 |
| 203790_s_at | heat-responsive protein 12 | HRSP12 | -43.7 | -6.4 | -16.8 |
| 215554_at | glycosylphosphatidylinositol specific phospholipase D1 | GPLD1 | -25.1 | -11.0 | -16.6 |
| 206292_s_at | sulfotransferase family, cytosolic, 2A, dehydroepiandrosterone (DHEA)-preferring, member 1 | SULT2A1 | -122.6 | -2.2 | -16.5 |
| 207041_at | mannan-binding lectin serine peptidase 2 | MASP2 | -41.7 | -6.3 | -16.3 |
| 208147_s_at | cytochrome P450, family 2, subfamily C, polypeptide 8 | CYP2C8 | -80.5 | -3.2 | -16.1 |
| 220224_at | hydroxyacid oxidase (glycolate oxidase) 1 | HAO1 | -85.4 | -3.1 | -16.1 |
| 204685_s_at | ATPase, Ca++ transporting, plasma membrane 2 | ATP2B2 | -35.7 | -6.6 | -15.4 |
| 205500_at | complement component 5 | C5 | -44.2 | -5.3 | -15.3 |
| 223699_at | carnosine dipeptidase 1 (metallopeptidase M20 family) | CNDP1 | -28.3 | -8.3 | -15.3 |
| 205999_x_at | cytochrome P450, family 3, subfamily A, polypeptide 4 | CYP3A4 | -25.4 | -8.8 | -15.0 |
| 206262_at | alcohol dehydrogenase 1C (class I), gamma polypeptide | ADH1C | -26.6 | -8.5 | -15.0 |
| 230951_at | erythrocyte membrane protein band 4.1 like 5 | EPB41L5 | -23.7 | -9.3 | -14.9 |
| 214421_x_at | cytochrome P450, family 2, subfamily C, polypeptide 9 | CYP2C9 | -67.6 | -3.3 | -14.8 |
| 214478_at | secreted phosphoprotein 2, 24kDa | SPP2 | -30.5 | -7.2 | -14.8 |
| 1559573_at | --- | --- | -35.0 | -6.2 | -14.7 |
| 205871_at | plasminogen-like A  plasminogen-like B1  plasminogen-like B2 | PLGLA PLGLB1 PLGLB2 | -49.4 | -4.3 | -14.6 |
| 235129_at | protein phosphatase 1, regulatory (inhibitor) subunit 1A | PPP1R1A | -15.2 | -13.8 | -14.5 |
| 209541_at | insulin-like growth factor 1 (somatomedin C) | IGF1 | -12.1 | -17.1 | -14.4 |
| 205675_at | microsomal triglyceride transfer protein | MTTP | -106.8 | -1.9 | -14.2 |
| 238625_at | chromosome 1 open reading frame 168 | C1orf168 | -31.5 | -6.4 | -14.2 |
| 204704_s_at | aldolase B, fructose-bisphosphate | ALDOB | -79.8 | -2.5 | -14.1 |
| 205355_at | acyl-CoA dehydrogenase, short/branched chain | ACADSB | -28.7 | -6.9 | -14.1 |
| 207810_at | coagulation factor XIII, B polypeptide | F13B | -40.9 | -4.7 | -13.8 |
| 206541_at | kallikrein B, plasma (Fletcher factor) 1 | KLKB1 | -25.9 | -7.0 | -13.4 |
| 221008_s_at | alanine-glyoxylate aminotransferase 2-like 1 | AGXT2L1 | -69.3 | -2.5 | -13.2 |
| 206910_x_at | complement factor H-related 2 | CFHR2 | -30.5 | -5.7 | -13.1 |
| 206610_s_at | coagulation factor XI | F11 | -26.9 | -6.2 | -13.0 |
| 242817_at | peptidoglycan recognition protein 2 | PGLYRP2 | -33.6 | -5.0 | -12.9 |
| 230602_at | aminocarboxymuconate semialdehyde decarboxylase | ACMSD | -33.8 | -4.9 | -12.8 |
| 238160_at | acyl-CoA thioesterase 12 | ACOT12 | -67.5 | -2.4 | -12.8 |
| 209614_at | alcohol dehydrogenase 1B (class I), beta polypeptide | ADH1B | -21.3 | -7.5 | -12.7 |
| 220507_s_at | ureidopropionase, beta | UPB1 | -43.4 | -3.7 | -12.7 |
| 220491_at | hepcidin antimicrobial peptide | HAMP | -63.8 | -2.4 | -12.4 |
| 241994_at | xanthine dehydrogenase | XDH | -35.4 | -4.4 | -12.4 |
| 206007_at | proteoglycan 4 | PRG4 | -12.9 | -11.8 | -12.3 |
| 206535_at | solute carrier family 2 (facilitated glucose transporter), member 2 | SLC2A2 | -38.9 | -3.9 | -12.3 |
| 210168_at | complement component 6 | C6 | -13.3 | -11.4 | -12.3 |
| 205498_at | growth hormone receptor | GHR | -31.3 | -4.8 | -12.2 |
| 225424_at | glycerol-3-phosphate acyltransferase, mitochondrial | GPAM | -29.7 | -4.9 | -12.1 |
| 202357_s_at | complement factor B | CFB | -26.1 | -5.5 | -11.9 |
| 205774_at | coagulation factor XII (Hageman factor) | F12 | -49.5 | -2.8 | -11.8 |
| 206211_at | selectin E | SELE | -14.4 | -9.8 | -11.8 |
| 219733_s_at | solute carrier family 27 (fatty acid transporter), member 5 | SLC27A5 | -22.8 | -6.0 | -11.7 |
| 227174_at | WD repeat domain 72 | WDR72 | -19.6 | -7.0 | -11.7 |
| 207185_at | solute carrier family 10 (sodium/bile acid cotransporter family), member 1 | SLC10A1 | -29.5 | -4.5 | -11.5 |
| 219902_at | betaine--homocysteine S-methyltransferase 2 | BHMT2 | -55.4 | -2.4 | -11.5 |
| 1570505_at | ATP-binding cassette, sub-family B (MDR/TAP), member 4 | ABCB4 | -56.6 | -2.3 | -11.3 |
| 206754_s_at | cytochrome P450, family 2, subfamily B, polypeptide 6  cytochrome P450, family 2, subfamily B, polypeptide 7 pseudogene 1 | CYP2B6  CYP2B7P1 | -46.0 | -2.8 | -11.3 |
| 206065_s_at | dihydropyrimidinase | DPYS | -38.5 | -3.3 | -11.2 |
| 219543_at | phenazine biosynthesis-like protein domain containing | PBLD | -25.1 | -4.9 | -11.1 |
| 206606_at | lipase, hepatic | LIPC | -23.4 | -5.2 | -11.0 |
| 207007_at | nuclear receptor subfamily 1, group I, member 3 | NR1I3 | -15.9 | -7.6 | -11.0 |
| 208451_s_at | complement component 4A (Rodgers blood group)  complement component 4B (Chido blood group) complement C4-B-like | C4A  C4B  LOC100509001 | -29.7 | -4.0 | -10.9 |
| 221590_s_at | Aldehyde dehydrogenase 6 family, member A1 | ALDH6A1 | -15.3 | -7.7 | -10.8 |
| 215559_at | ATP-binding cassette, sub-family C (CFTR/MRP), member 6 | ABCC6 | -33.2 | -3.5 | -10.7 |
| 225516_at | solute carrier family 7 (cationic amino acid transporter, y+ system), member 2 | SLC7A2 | -22.3 | -5.1 | -10.7 |
| 228293_at | DEP domain containing 7 | DEPDC7 | -40.4 | -2.9 | -10.7 |
| 205305_at | fibrinogen-like 1 | FGL1 | -43.6 | -2.6 | -10.6 |
| 207330_at | pregnancy-zone protein | PZP | -11.0 | -10.1 | -10.5 |
| 214261_s_at | alcohol dehydrogenase 6 (class V) | ADH6 | -42.9 | -2.6 | -10.5 |
| 233030_at | patatin-like phospholipase domain containing 3 | PNPLA3 | -19.5 | -5.7 | -10.5 |
| 220116_at | potassium intermediate/small conductance calcium-activated channel, subfamily N, member 2 | KCNN2 | -11.2 | -9.6 | -10.4 |
| 231359_at | Apolipoprotein H (beta-2-glycoprotein I) | APOH | -16.3 | -6.6 | -10.4 |
| 1552362_a_at | liver expressed antimicrobial peptide 2 | LEAP2 | -41.0 | -2.6 | -10.3 |
| 230318_at | serpin peptidase inhibitor, clade A (alpha-1 antiproteinase, antitrypsin), member 1 | SERPINA1 | -39.8 | -2.7 | -10.3 |
| 205531_s_at | glutaminase 2 (liver, mitochondrial) | GLS2 | -17.7 | -5.9 | -10.2 |
| 206239_s_at | serine peptidase inhibitor, Kazal type 1 | SPINK1 | -36.3 | -2.8 | -10.0 |
| 213631_x_at | dihydroorotate dehydrogenase | DHODH | -16.3 | -6.2 | -10.0 |
| 222943_at | glucosidase, beta, acid 3 (cytosolic) | GBA3 | -22.3 | -4.4 | -10.0 |
| 229299_at | chromosome 5 open reading frame 33 | C5orf33 | -26.1 | -3.8 | -9.9 |
| 1558322_a_at | progestin and adipoQ receptor family member IX | PAQR9 | -23.3 | -4.1 | -9.8 |
| 205075_at | serpin peptidase inhibitor, clade F (alpha-2 antiplasmin, pigment epithelium derived factor), member 2 | SERPINF2 | -30.0 | -3.2 | -9.8 |
| 205433_at | butyrylcholinesterase | BCHE | -25.5 | -3.7 | -9.7 |
| 205749_at | cytochrome P450, family 1, subfamily A, polypeptide 1 | CYP1A1 | -11.5 | -8.2 | -9.7 |
| 206085_s_at | cystathionase (cystathionine gamma-lyase) | CTH | -27.8 | -3.4 | -9.7 |
| 218976_at | DnaJ (Hsp40) homolog, subfamily C, member 12 | DNAJC12 | -22.8 | -4.0 | -9.6 |
| 219295_s_at | procollagen C-endopeptidase enhancer 2 | PCOLCE2 | -10.1 | -8.7 | -9.4 |
| 223278_at | gap junction protein, beta 2, 26kDa | GJB2 | -29.5 | -3.0 | -9.4 |
| 226192_at | androgen receptor | AR | -16.9 | -5.3 | -9.4 |
| 205158_at | ribonuclease, RNase A family, 4 | RNASE4 | -18.0 | -4.9 | -9.3 |
| 204534_at | vitronectin | VTN | -25.0 | -3.4 | -9.2 |
| 210942_s_at | ST3 beta-galactoside alpha-2,3-sialyltransferase 6 | ST3GAL6 | -11.0 | -7.8 | -9.2 |
| 215363_x_at | folate hydrolase (prostate-specific membrane antigen) 1 | FOLH1 | -11.2 | -7.6 | -9.2 |
| 241703_at | RUN domain containing 3B | RUNDC3B | -16.4 | -5.1 | -9.2 |
| 213245_at | adenylate cyclase 1 (brain) | ADCY1 | -8.2 | -10.2 | -9.1 |
| 227417_at | MOCO sulphurase C-terminal domain containing 2 | MOSC2 | -9.5 | -8.8 | -9.1 |
| 89977_at | acyl-CoA synthetase medium-chain family member 5 | ACSM5 | -13.3 | -6.3 | -9.1 |
| 204934_s_at | hepsin | HPN | -23.1 | -3.5 | -9.0 |
| 205066_s_at | ectonucleotide pyrophosphatase/phosphodiesterase 1 | ENPP1 | -22.0 | -3.7 | -9.0 |
| 209616_s_at | carboxylesterase 1 | CES1 | -53.0 | -1.5 | -9.0 |
| 219093_at | phosphotyrosine interaction domain containing 1 | PID1 | -9.2 | -8.8 | -9.0 |
| 212110_at | solute carrier family 39 (zinc transporter), member 14 | SLC39A14 | -21.7 | -3.6 | -8.9 |
| 205776_at | flavin containing monooxygenase 5 | FMO5 | -16.4 | -4.7 | -8.8 |
| 213920_at | cut-like homeobox 2 | CUX2 | -17.4 | -4.4 | -8.8 |
| 204476_s_at | pyruvate carboxylase | PC | -24.6 | -3.1 | -8.7 |
| 215695_s_at | glycogenin 2 | GYG2 | -18.8 | -4.0 | -8.7 |
| 222083_at | glycine-N-acyltransferase | GLYAT | -21.0 | -3.5 | -8.6 |
| 235182_at | isthmin 1 homolog (zebrafish) | ISM1 | -9.1 | -8.1 | -8.6 |
| 1554640_at | paralemmin 2 | PALM2 | -14.7 | -4.9 | -8.5 |
| 203608_at | aldehyde dehydrogenase 5 family, member A1 | ALDH5A1 | -14.0 | -5.1 | -8.5 |
| 220528_at | vanin 3 | VNN3 | -11.4 | -6.3 | -8.5 |
| 205568_at | aquaporin 9 | AQP9 | -15.7 | -4.4 | -8.3 |
| 226649_at | pantothenate kinase 1 | PANK1 | -17.8 | -3.9 | -8.3 |
| 239562_at | methylenetetrahydrofolate dehydrogenase (NADP+ dependent) 2-like | MTHFD2L | -9.5 | -7.3 | -8.3 |
| 241547_at | --- | --- | -21.1 | -3.3 | -8.3 |
| 205755_at | inter-alpha (globulin) inhibitor H3 | ITIH3 | -29.4 | -2.3 | -8.2 |
| 229053_at | synaptotagmin XVII | SYT17 | -11.2 | -6.0 | -8.2 |
| 243483_at | transient receptor potential cation channel, subfamily M, member 8 | TRPM8 | -17.5 | -3.8 | -8.2 |
| 1555229_a_at | complement component 1, s subcomponent | C1S | -8.4 | -7.9 | -8.1 |
| 201627_s_at | insulin induced gene 1 | INSIG1 | -15.2 | -4.3 | -8.1 |
| 204998_s_at | activating transcription factor 5 | ATF5 | -24.6 | -2.7 | -8.1 |
| 231694_at | apolipoprotein A-I* | APOA1 | -16.1 | -4.1 | -8.1 |
| 236430_at | transmembrane emp24 protein transport domain containing 6 | TMED6 | -14.7 | -4.4 | -8.1 |
| 222043_at | clusterin | CLU | -11.5 | -5.5 | -8.0 |
| 223582_at | G protein-coupled receptor 98 | GPR98 | -13.3 | -4.8 | -8.0 |
| 227828_s_at | family with sequence similarity 176, member A | FAM176A | -25.7 | -2.5 | -8.0 |
| 229777_at | clarin 3 | CLRN3 | -33.2 | -1.9 | -8.0 |
| 206797_at | N-acetyltransferase 2 (arylamine N-acetyltransferase) | NAT2 | -24.4 | -2.6 | -7.9 |
| 211303_x_at | folate hydrolase 1B | FOLH1B | -10.9 | -5.7 | -7.9 |
| 205421_at | solute carrier family 22 (extraneuronal monoamine transporter), member 3 | SLC22A3 | -23.5 | -2.6 | -7.8 |
| 235299_at | solute carrier family 41, member 2 | SLC41A2 | -12.4 | -4.8 | -7.8 |
| 237350_at | tetratricopeptide repeat domain 36 | TTC36 | -11.1 | -5.4 | -7.8 |
| 202886_s_at | protein phosphatase 2, regulatory subunit A, beta | PPP2R1B | -13.9 | -4.2 | -7.7 |
| 213664_at | solute carrier family 1 (neuronal/epithelial high affinity glutamate transporter, system Xag), member 1 | SLC1A1 | -8.7 | -6.9 | -7.7 |
| 215103_at | cytochrome P450, family 2, subfamily C, polypeptide 18 | CYP2C18 | -22.6 | -2.6 | -7.7 |
| 216733_s_at | glycine amidinotransferase (L-arginine:glycine amidinotransferase) | GATM | -12.7 | -4.6 | -7.7 |
| 205042_at | glucosamine (UDP-N-acetyl)-2-epimerase/N-acetylmannosamine kinase | GNE | -10.6 | -5.4 | -7.6 |
| 205222_at | enoyl-CoA, hydratase/3-hydroxyacyl CoA dehydrogenase | EHHADH | -28.4 | -2.0 | -7.6 |
| 231029_at | coagulation factor V (proaccelerin, labile factor) | F5 | -10.1 | -5.7 | -7.6 |
| 210558_at | aldo-keto reductase family 1, member C4 (chlordecone reductase; 3-alpha hydroxysteroid dehydrogenase, type I; dihydrodiol dehydrogenase 4) | AKR1C4 | -26.5 | -2.1 | -7.5 |
| 210751_s_at | regucalcin (senescence marker protein-30) | RGN | -15.2 | -3.7 | -7.5 |
| 1555564_a_at | complement factor I | CFI | -9.5 | -5.8 | -7.4 |
| 211596_s_at | leucine-rich repeats and immunoglobulin-like domains 1 | LRIG1 | -5.8 | -9.6 | -7.4 |
| 228463_at | forkhead box A3 | FOXA3 | -14.4 | -3.8 | -7.4 |
| 236652_at | hypothetical protein LOC149703 | LOC149703 | -23.3 | -2.3 | -7.4 |
| 1554533_at | complement component 2 | C2 | -9.6 | -5.6 | -7.3 |
| 203560_at | gamma-glutamyl hydrolase (conjugase, folylpolygammaglutamyl hydrolase) | GGH | -14.7 | -3.6 | -7.3 |
| 203642_s_at | COBL-like 1 | COBLL1 | -8.9 | -6.0 | -7.3 |
| 205404_at | hydroxysteroid (11-beta) dehydrogenase 1 | HSD11B1 | -11.1 | -4.8 | -7.3 |
| 218326_s_at | leucine-rich repeat-containing G protein-coupled receptor 4 | LGR4 | -10.5 | -5.1 | -7.3 |
| 225792_at | hook homolog 1 (Drosophila) | HOOK1 | -10.5 | -5.1 | -7.3 |
| 229430_at | chromosome 8 open reading frame 46 | C8orf46 | -8.3 | -6.5 | -7.3 |
| 206204_at | growth factor receptor-bound protein 14 | GRB14 | -10.8 | -4.8 | -7.2 |
| 207498_s_at | cytochrome P450, family 2, subfamily D, polypeptide 6 | CYP2D6 | -21.0 | -2.5 | -7.2 |
| 220161_s_at | erythrocyte membrane protein band 4.1 like 4B | EPB41L4B | -12.5 | -4.2 | -7.2 |
| 231559_at | hypothetical LOC100506941 | LOC100506941 | -10.5 | -4.9 | -7.2 |
| 236302_at | protein phosphatase, Mg2+/Mn2+ dependent, 1E | PPM1E | -7.8 | -6.6 | -7.2 |
| 1556666_a_at | non-protein coding RNA 291 | NCRNA00291 | -7.1 | -7.2 | -7.1 |
| 206069_s_at | acyl-CoA dehydrogenase, long chain | ACADL | -9.8 | -5.1 | -7.1 |
| 217546_at | metallothionein 1M | MT1M | -11.5 | -4.4 | -7.1 |
| 221024_s_at | solute carrier family 2 (facilitated glucose transporter), member 10 | SLC2A10 | -7.7 | -6.5 | -7.1 |
| 1553296_at | G protein-coupled receptor 128 | GPR128 | -4.1 | -11.8 | -7.0 |
| 1554830_a_at | STEAP family member 3 | STEAP3 | -13.2 | -3.7 | -7.0 |
| 205682_x_at | apolipoprotein M | APOM | -26.7 | -1.8 | -7.0 |
| 209301_at | carbonic anhydrase II | CA2 | -6.7 | -7.2 | -7.0 |
| 216058_s_at | cytochrome P450, family 2, subfamily C, polypeptide 19 | CYP2C19 | -12.9 | -3.8 | -7.0 |
| 219277_s_at | oxoglutarate dehydrogenase-like | OGDHL | -13.9 | -3.6 | -7.0 |
| 220313_at | G protein-coupled receptor 88 | GPR88 | -9.4 | -5.2 | -7.0 |
| 221245_s_at | frizzled homolog 5 (Drosophila) | FZD5 | -8.9 | -5.5 | -7.0 |
| 206496_at | flavin containing monooxygenase 3 | FMO3 | -15.7 | -3.0 | -6.9 |
| 212158_at | syndecan 2 | SDC2 | -11.5 | -4.1 | -6.9 |
| 229276_at | immunoglobulin superfamily, member 9 | IGSF9 | -6.8 | -6.9 | -6.9 |
| 238576_at | molybdenum cofactor sulfurase | MOCOS | -10.0 | -4.7 | -6.9 |
| 239860_at | LP2209 | LOC100130232 | -19.5 | -2.4 | -6.9 |
| 219718_at | FGGY carbohydrate kinase domain containing | FGGY | -8.7 | -5.3 | -6.8 |
| 223605_at | solute carrier family 25 (mitochondrial carrier), member 18 | SLC25A18 | -19.6 | -2.4 | -6.8 |
| 229377_at | growth hormone regulated TBC protein 1 | GRTP1 | -11.9 | -3.9 | -6.8 |
| 204388_s_at | monoamine oxidase A | MAOA | -16.8 | -2.7 | -6.7 |
| 213832_at | potassium voltage-gated channel, Shal-related subfamily, member 3 | KCND3 | -5.8 | -7.8 | -6.7 |
| 214064_at | transferrin | TF | -8.6 | -5.2 | -6.7 |
| 214156_at | myosin VIIA and Rab interacting protein | MYRIP | -7.6 | -5.9 | -6.7 |
| 220435_at | solute carrier family 30, member 10 | SLC30A10 | -12.1 | -3.7 | -6.7 |
| 230573_at | Serum/glucocorticoid regulated kinase 2 | SGK2 | -14.3 | -3.1 | -6.7 |
| 231009_at | phospholipase A2, group XIIB | PLA2G12B | -22.5 | -2.0 | -6.7 |
| 205542_at | six transmembrane epithelial antigen of the prostate 1 | STEAP1 | -13.1 | -3.3 | -6.6 |
| 206259_at | protein C (inactivator of coagulation factors Va and VIIIa) | PROC | -12.6 | -3.4 | -6.6 |
| 211028_s_at | ketohexokinase (fructokinase) | KHK | -15.9 | -2.7 | -6.6 |
| 212883_at | Apolipoprotein E | APOE | -7.9 | -5.5 | -6.6 |
| 212907_at | solute carrier family 30 (zinc transporter), member 1 | SLC30A1 | -8.4 | -5.1 | -6.6 |
| 231698_at | hypothetical LOC647115 | FLJ36848 | -8.7 | -5.0 | -6.6 |
| 236175_at | tripartite motif-containing 55 | TRIM55 | -16.2 | -2.7 | -6.6 |
| 238956_at | hypothetical LOC100506781 | LOC100506781 | -16.1 | -2.7 | -6.6 |
| 1560469_at | nuclear receptor subfamily 5, group A, member 2 | NR5A2 | -6.1 | -6.8 | -6.5 |
| 207275_s_at | acyl-CoA synthetase long-chain family member 1 | ACSL1 | -14.5 | -2.9 | -6.5 |
| 202238_s_at | nicotinamide N-methyltransferase | NNMT | -16.4 | -2.5 | -6.4 |
| 205073_at | cytochrome P450, family 2, subfamily J, polypeptide 2 | CYP2J2 | -16.4 | -2.5 | -6.4 |
| 206938_at | steroid-5-alpha-reductase, alpha polypeptide 2 (3-oxo-5 alpha-steroid delta 4-dehydrogenase alpha 2) | SRD5A2 | -8.9 | -4.7 | -6.4 |
| 207202_s_at | nuclear receptor subfamily 1, group I, member 2 | NR1I2 | -22.6 | -1.8 | -6.4 |
| 211423_s_at | sterol-C5-desaturase (ERG3 delta-5-desaturase homolog, S. cerevisiae)-like | SC5DL | -5.3 | -7.7 | -6.4 |
| 211715_s_at | 3-hydroxybutyrate dehydrogenase, type 1 | BDH1 | -13.7 | -3.0 | -6.4 |
| 228806_at | RAR-related orphan receptor C | RORC | -9.6 | -4.3 | -6.4 |
| 229273_at | sal-like 1 (Drosophila) | SALL1 | -14.1 | -2.9 | -6.4 |
| 231693_at | Fatty acid binding protein 1, liver | FABP1 | -22.9 | -1.8 | -6.4 |
| 1552307_a_at | tetratricopeptide repeat domain 39C | TTC39C | -12.9 | -3.0 | -6.3 |
| 1552569_a_at | receptor (chemosensory) transporter protein 3 | RTP3 | -18.7 | -2.1 | -6.3 |
| 203434_s_at | membrane metallo-endopeptidase | MME | -11.4 | -3.5 | -6.3 |
| 205939_at | cytochrome P450, family 3, subfamily A, polypeptide 7 | CYP3A7 | -11.1 | -3.6 | -6.3 |
| 216320_x_at | macrophage stimulating 1 (hepatocyte growth factor-like) | MST1 | -16.6 | -2.4 | -6.3 |
| 221556_at | CDC14 cell division cycle 14 homolog B (S. cerevisiae) | CDC14B | -8.1 | -5.0 | -6.3 |
| 226438_at | syntrophin, beta 1 (dystrophin-associated protein A1, 59kDa, basic component 1) | SNTB1 | -9.3 | -4.3 | -6.3 |
| 226750_at | La ribonucleoprotein domain family, member 1B | LARP1B | -9.0 | -4.4 | -6.3 |
| 209368_at | epoxide hydrolase 2, cytoplasmic | EPHX2 | -14.2 | -2.7 | -6.2 |
| 214234_s_at | cytochrome P450, family 3, subfamily A, polypeptide 5 | CYP3A5 | -13.0 | -3.0 | -6.2 |
| 229103_at | wingless-type MMTV integration site family, member 3 | WNT3 | -7.7 | -5.0 | -6.2 |
| 205943_at | tryptophan 2,3-dioxygenase | TDO2 | -11.2 | -3.3 | -6.1 |
| 207354_at | chemokine (C-C motif) ligand 16 | CCL16 | -12.3 | -3.0 | -6.1 |
| 209980_s_at | serine hydroxymethyltransferase 1 (soluble) | SHMT1 | -9.7 | -3.9 | -6.1 |
| 212816_s_at | cystathionine-beta-synthase | CBS | -13.1 | -2.9 | -6.1 |
| 219181_at | lipase, endothelial | LIPG | -8.4 | -4.4 | -6.1 |
| 235591_at | somatostatin receptor 1 | SSTR1 | -6.8 | -5.4 | -6.1 |
| 219410_at | transmembrane protein 45A | TMEM45A | -9.1 | -4.0 | -6.0 |
| 204987_at | inter-alpha (globulin) inhibitor H2 | ITIH2 | -12.6 | -2.8 | -5.9 |
| 205311_at | dopa decarboxylase (aromatic L-amino acid decarboxylase) | DDC | -18.0 | -1.9 | -5.9 |
| 205969_at | arylacetamide deacetylase (esterase) | AADAC | -21.4 | -1.6 | -5.9 |
| 227476_at | lysophosphatidylglycerol acyltransferase 1 | LPGAT1 | -6.6 | -5.2 | -5.9 |
| 203997_at | protein tyrosine phosphatase, non-receptor type 3 | PTPN3 | -9.2 | -3.7 | -5.8 |
| 204140_at | tyrosylprotein sulfotransferase 1 | TPST1 | -5.6 | -6.1 | -5.8 |
| 207300_s_at | coagulation factor VII (serum prothrombin conversion accelerator) | F7 | -11.1 | -3.1 | -5.8 |
| 229223_at | epithelial splicing regulatory protein 2 | ESRP2 | -8.6 | -3.9 | -5.8 |
| 229693_at | transmembrane protein 220 | TMEM220 | -7.2 | -4.6 | -5.8 |
| 236235_at | Itchy E3 ubiquitin protein ligase homolog (mouse) | ITCH | -7.1 | -4.8 | -5.8 |
| 237086_at | Forkhead box A1 | FOXA1 | -5.9 | -5.7 | -5.8 |
| 207773_x_at | cytochrome P450, family 3, subfamily A, polypeptide 43 | CYP3A43 | -9.0 | -3.6 | -5.7 |
| 212657_s_at | interleukin 1 receptor antagonist | IL1RN | -9.6 | -3.4 | -5.7 |
| 213143_at | chromosome 2 open reading frame 72 | C2orf72 | -11.6 | -2.8 | -5.7 |
| 213712_at | elongation of very long chain fatty acids (FEN1/Elo2, SUR4/Elo3, yeast)-like 2 | ELOVL2 | -9.3 | -3.5 | -5.7 |
| 213929_at | exophilin 5 | EXPH5 | -7.6 | -4.3 | -5.7 |
| 230914_at | hepatocyte nuclear factor 4, alpha | HNF4A | -13.3 | -2.5 | -5.7 |
| 240509_s_at | gremlin 2 | GREM2 | -8.2 | -3.9 | -5.7 |
| 205412_at | acetyl-CoA acetyltransferase 1 | ACAT1 | -7.5 | -4.2 | -5.6 |
| 216223_at | carboxypeptidase N, polypeptide 2 | CPN2 | -15.9 | -2.0 | -5.6 |
| 219076_s_at | peroxisomal membrane protein 2, 22kDa | PXMP2 | -8.2 | -3.8 | -5.6 |
| 220532_s_at | transmembrane protein 176B | TMEM176B | -9.1 | -3.5 | -5.6 |
| 228716_at | thyroid hormone receptor, beta (erythroblastic leukemia viral (v-erb-a) oncogene homolog 2, avian) | THRB | -7.9 | -3.9 | -5.6 |
| 1552727_s_at | ADAM metallopeptidase with thrombospondin type 1 motif, 17 | ADAMTS17 | -6.3 | -4.8 | -5.5 |
| 201120_s_at | progesterone receptor membrane component 1 | PGRMC1 | -7.9 | -3.8 | -5.5 |
| 206373_at | Zic family member 1 (odd-paired homolog, Drosophila) | ZIC1 | -7.4 | -4.1 | -5.5 |
| 213325_at | poliovirus receptor-related 3 | PVRL3 | -6.0 | -5.1 | -5.5 |
| 219525_at | solute carrier family 47, member 1 | SLC47A1 | -9.0 | -3.3 | -5.5 |
| 200730_s_at | protein tyrosine phosphatase type IVA, member 1 | PTP4A1 | -9.4 | -3.1 | -5.4 |
| 219313_at | GRAM domain containing 1C | GRAMD1C | -5.2 | -5.5 | -5.4 |
| 224299_x_at | formiminotransferase cyclodeaminase | FTCD | -13.6 | -2.1 | -5.4 |
| 227550_at | GDNF family receptor alpha 1 | GFRA1 | -5.9 | -5.0 | -5.4 |
| 238513_at | Proline rich Gla (G-carboxyglutamic acid) 4 (transmembrane) | PRRG4 | -5.0 | -6.0 | -5.4 |
| 49452_at | acetyl-CoA carboxylase beta | ACACB | -5.6 | -5.1 | -5.4 |
| 204753_s_at | hepatic leukemia factor | HLF | -14.2 | -2.0 | -5.3 |
| 205208_at | aldehyde dehydrogenase 1 family, member L1 | ALDH1L1 | -13.8 | -2.0 | -5.3 |
| 205848_at | growth arrest-specific 2 | GAS2 | -9.7 | -2.9 | -5.3 |
| 208116_s_at | mannosidase, alpha, class 1A, member 1 | MAN1A1 | -6.2 | -4.5 | -5.3 |
| 209146_at | sterol-C4-methyl oxidase-like | SC4MOL | -8.8 | -3.2 | -5.3 |
| 217504_at | ATP-binding cassette, sub-family A (ABC1), member 6 | ABCA6 | -5.6 | -5.1 | -5.3 |
| 227759_at | proprotein convertase subtilisin/kexin type 9 | PCSK9 | -5.6 | -5.1 | -5.3 |
| 229019_at | zinc finger protein 385B | ZNF385B | -5.6 | -5.1 | -5.3 |
| 231704_at | zinc finger protein 498 | ZNF498 | -5.6 | -4.9 | -5.3 |
| 240883_at | 6-phosphofructo-2-kinase/fructose-2,6-biphosphatase 1 | PFKFB1 | -6.1 | -4.6 | -5.3 |
| 200947_s_at | glutamate dehydrogenase 1 | GLUD1 | -8.9 | -3.0 | -5.2 |
| 201968_s_at | phosphoglucomutase 1 | PGM1 | -8.6 | -3.1 | -5.2 |
| 204624_at | ATPase, Cu++ transporting, beta polypeptide | ATP7B | -8.6 | -3.1 | -5.2 |
| 210377_at | acyl-CoA synthetase medium-chain family member 3 | ACSM3 | -5.7 | -4.8 | -5.2 |
| 214971_s_at | ST6 beta-galactosamide alpha-2,6-sialyltranferase 1 | ST6GAL1 | -6.0 | -4.5 | -5.2 |
| 225024_at | regulation of nuclear pre-mRNA domain containing 1B | RPRD1B | -6.2 | -4.4 | -5.2 |
| 227669_at | brain protein 44 | BRP44 | -6.5 | -4.2 | -5.2 |
| 229491_at | Na+/H+ exchanger domain containing 2 | NHEDC2 | -10.1 | -2.7 | -5.2 |
| 206130_s_at | asialoglycoprotein receptor 2 | ASGR2 | -12.4 | -2.1 | -5.1 |
| 209668_x_at | carboxylesterase 2 | CES2 | -13.1 | -2.0 | -5.1 |
| 219274_at | tetraspanin 12 | TSPAN12 | -5.6 | -4.7 | -5.1 |
| 222830_at | grainyhead-like 1 (Drosophila) | GRHL1 | -10.3 | -2.5 | -5.1 |
| 223579_s_at | apolipoprotein B (including Ag(x) antigen) | APOB | -10.0 | -2.6 | -5.1 |
| 233496_s_at | cofilin 2 (muscle) | CFL2 | -6.5 | -4.0 | -5.1 |
| 202023_at | ephrin-A1 | EFNA1 | -6.7 | -3.7 | -5.0 |
| 202843_at | DnaJ (Hsp40) homolog, subfamily B, member 9 | DNAJB9 | -6.4 | -4.0 | -5.0 |
| 221163_s_at | MLX interacting protein-like | MLXIPL | -8.6 | -2.9 | -5.0 |
| 223062_s_at | phosphoserine aminotransferase 1 | PSAT1 | -13.2 | -1.9 | -5.0 |
| 223437_at | peroxisome proliferator-activated receptor alpha | PPARA | -7.5 | -3.3 | -5.0 |
| 244385_at | Lysine (K)-specific demethylase 4C | KDM4C | -9.8 | -2.5 | -5.0 |
| 203799_at | CD302 molecule | CD302 | -4.3 | -5.7 | -4.9 |
| 204719_at | ATP-binding cassette, sub-family A (ABC1), member 8 | ABCA8 | -4.0 | -6.0 | -4.9 |
| 208561_at | ATP-binding cassette, sub-family C (CFTR/MRP), member 9 | ABCC9 | -4.7 | -5.1 | -4.9 |
| 209774_x_at | chemokine (C-X-C motif) ligand 2 | CXCL2 | -8.1 | -2.9 | -4.9 |
| 214829_at | aminoadipate-semialdehyde synthase | AASS | -5.4 | -4.5 | -4.9 |
| 215794_x_at | glutamate dehydrogenase 2 | GLUD2 | -7.9 | -3.0 | -4.9 |
| 222614_at | RWD domain containing 2B | RWDD2B | -5.4 | -4.4 | -4.9 |
| 222662_at | protein phosphatase 1, regulatory (inhibitor) subunit 3B | PPP1R3B | -7.8 | -3.1 | -4.9 |
| 225573_at | acyl-CoA dehydrogenase family, member 11  nephronophthisis 3 (adolescent) | ACAD11 NPHP3 | -8.8 | -2.8 | -4.9 |
| 205773_at | cytoplasmic polyadenylation element binding protein 3 | CPEB3 | -5.0 | -4.6 | -4.8 |
| 207717_s_at | plakophilin 2 | PKP2 | -6.6 | -3.6 | -4.8 |
| 209608_s_at | acetyl-CoA acetyltransferase 2 | ACAT2 | -5.7 | -3.9 | -4.8 |
| 210473_s_at | G protein-coupled receptor 125 | GPR125 | -4.7 | -4.9 | -4.8 |
| 210653_s_at | branched chain keto acid dehydrogenase E1, beta polypeptide | BCKDHB | -7.4 | -3.1 | -4.8 |
| 217583_at | phenylalanine hydroxylase | PAH | -7.9 | -2.9 | -4.8 |
| 217738_at | nicotinamide phosphoribosyltransferase | NAMPT | -6.5 | -3.6 | -4.8 |
| 220135_s_at | solute carrier family 7 (cationic amino acid transporter, y+ system), member 9 | SLC7A9 | -16.3 | -1.4 | -4.8 |
| 221477_s_at | superoxide dismutase 2, mitochondrial | SOD2 | -5.9 | -3.9 | -4.8 |
| 227197_at | Rho guanine nucleotide exchange factor (GEF) 26 | ARHGEF26 | -5.8 | -4.0 | -4.8 |
| 230782_at | sorbitol dehydrogenase | SORD | -7.7 | -3.0 | -4.8 |
| 231587_at | Apolipoprotein C-III | APOC3 | -11.4 | -2.0 | -4.8 |
| 236236_at | WNK lysine deficient protein kinase 3 | WNK3 | -4.9 | -4.8 | -4.8 |
| 239001_at | Microsomal glutathione S-transferase 1 | MGST1 | -10.5 | -2.2 | -4.8 |
| 241193_at | V-ets erythroblastosis virus E26 oncogene homolog 2 (avian) | ETS2 | -4.8 | -4.8 | -4.8 |
| 203566_s_at | amylo-alpha-1, 6-glucosidase, 4-alpha-glucanotransferase | AGL | -5.9 | -3.8 | -4.7 |
| 203722_at | aldehyde dehydrogenase 4 family, member A1 | ALDH4A1 | -8.3 | -2.6 | -4.7 |
| 207027_at | HGF activator | HGFAC | -9.4 | -2.3 | -4.7 |
| 209712_at | solute carrier family 35 (UDP-glucuronic acid/UDP-N-acetylgalactosamine dual transporter), member D1 | SLC35D1 | -7.6 | -2.9 | -4.7 |
| 214307_at | homogentisate 1,2-dioxygenase | HGD | -13.1 | -1.7 | -4.7 |
| 231561_s_at | Apolipoprotein C-II | APOC2 | -10.3 | -2.2 | -4.7 |
| 237248_at | phosphodiesterase 11A | PDE11A | -7.5 | -2.9 | -4.7 |
| 1561094_a_at | solute carrier family 22, member 25 | SLC22A25 | -5.6 | -3.7 | -4.6 |
| 202718_at | insulin-like growth factor binding protein 2, 36kDa | IGFBP2 | -5.8 | -3.6 | -4.6 |
| 204121_at | growth arrest and DNA-damage-inducible, gamma | GADD45G | -6.5 | -3.3 | -4.6 |
| 204428_s_at | lecithin-cholesterol acyltransferase | LCAT | -5.8 | -3.6 | -4.6 |
| 218546_at | chromosome 1 open reading frame 115 | C1orf115 | -6.9 | -3.1 | -4.6 |
| 225807_at | jub, ajuba homolog (Xenopus laevis) | JUB | -6.5 | -3.3 | -4.6 |
| 227620_at | solute carrier family 44, member 1 | SLC44A1 | -4.8 | -4.4 | -4.6 |
| 232124_at | family with sequence similarity 198, member A | FAM198A | -4.6 | -4.7 | -4.6 |
| 232184_at | amyotrophic lateral sclerosis 2 (juvenile) | ALS2 | -5.2 | -4.1 | -4.6 |
| 33494_at | electron-transferring-flavoprotein dehydrogenase | ETFDH | -8.2 | -2.6 | -4.6 |
| 1559033_at | hypothetical LOC255167 | LOC255167 | -4.3 | -4.8 | -4.5 |
| 202735_at | emopamil binding protein (sterol isomerase) | EBP | -6.5 | -3.0 | -4.5 |
| 204675_at | steroid-5-alpha-reductase, alpha polypeptide 1 (3-oxo-5 alpha-steroid delta 4-dehydrogenase alpha 1) | SRD5A1 | -7.5 | -2.7 | -4.5 |
| 205480_s_at | UDP-glucose pyrophosphorylase 2 | UGP2 | -6.2 | -3.3 | -4.5 |
| 209535_s_at | non-ocogenic Rho GTPase-specific GTP exchange factor (proto-LBC) mRNA* | AKAP13 | -4.4 | -4.6 | -4.5 |
| 227491_at | ELOVL family member 6, elongation of long chain fatty acids (FEN1/Elo2, SUR4/Elo3-like, yeast) | ELOVL6 | -7.4 | -2.7 | -4.5 |
| 228242_at | NEDD4 binding protein 2 | N4BP2 | -4.7 | -4.3 | -4.5 |
| 228469_at | Peptidylprolyl isomerase D | PPID | -6.9 | -3.0 | -4.5 |
| 228834_at | transducer of ERBB2, 1 | TOB1 | -4.5 | -4.4 | -4.5 |
| 231015_at | Kruppel-like factor 15 | KLF15 | -5.3 | -3.9 | -4.5 |
| 201849_at | BCL2/adenovirus E1B 19kDa interacting protein 3 | BNIP3 | -8.7 | -2.2 | -4.4 |
| 202283_at | serpin peptidase inhibitor, clade F (alpha-2 antiplasmin, pigment epithelium derived factor), member 1 | SERPINF1 | -4.3 | -4.5 | -4.4 |
| 204120_s_at | adenosine kinase | ADK | -7.1 | -2.8 | -4.4 |
| 205364_at | acyl-CoA oxidase 2, branched chain | ACOX2 | -9.7 | -2.0 | -4.4 |
| 206325_at | serpin peptidase inhibitor, clade A (alpha-1 antiproteinase, antitrypsin), member 6 | SERPINA6 | -9.3 | -2.1 | -4.4 |
| 209366_x_at | cytochrome b5 type A (microsomal) | CYB5A | -8.2 | -2.3 | -4.4 |
| 210512_s_at | vascular endothelial growth factor A | VEGFA | -4.7 | -4.1 | -4.4 |
| 215129_at | phosphoinositide-3-kinase, class 2, gamma polypeptide | PIK3C2G | -5.7 | -3.4 | -4.4 |
| 218170_at | isochorismatase domain containing 1 | ISOC1 | -7.1 | -2.7 | -4.4 |
| 223874_at | ARP3 actin-related protein 3 homolog C (yeast) | ACTR3C | -6.4 | -3.1 | -4.4 |
| 226597_at | receptor accessory protein 6 | REEP6 | -11.7 | -1.7 | -4.4 |
| 228004_at | non-protein coding RNA 261 | NCRNA00261 | -8.4 | -2.3 | -4.4 |
| 232449_at | beta-carotene oxygenase 2 | BCO2 | -5.6 | -3.5 | -4.4 |
| 238530_at | nicotinamide nucleotide transhydrogenase | NNT | -4.7 | -4.1 | -4.4 |
| 241981_at | family with sequence similarity 20, member A | FAM20A | -3.3 | -5.9 | -4.4 |
| 1554375_a_at | nuclear receptor subfamily 1, group H, member 4 | NR1H4 | -3.3 | -5.6 | -4.3 |
| 205351_at | gamma-glutamyl carboxylase | GGCX | -6.4 | -2.8 | -4.3 |
| 206424_at | cytochrome P450, family 26, subfamily A, polypeptide 1 | CYP26A1 | -4.3 | -4.3 | -4.3 |
| 206738_at | apolipoprotein C-IV | APOC4 | -9.4 | -2.0 | -4.3 |
| 209894_at | leptin receptor | LEPR | -9.7 | -1.9 | -4.3 |
| 213974_at | ADAMTS-like 3 | ADAMTSL3 | -3.3 | -5.6 | -4.3 |
| 214425_at | Alpha-1-microglobulin/bikunin precursor | AMBP | -5.8 | -3.3 | -4.3 |
| 222049_s_at | Retinol binding protein 4, plasma | RBP4 | -7.9 | -2.4 | -4.3 |
| 238518_x_at | glycerate kinase | GLYCTK | -7.0 | -2.6 | -4.3 |
| 1563475_s_at | chromosome 12 open reading frame 72 | C12orf72 | -4.0 | -4.6 | -4.2 |
| 202068_s_at | low density lipoprotein receptor | LDLR | -5.0 | -3.6 | -4.2 |
| 202376_at | serpin peptidase inhibitor, clade A (alpha-1 antiproteinase, antitrypsin), member 3 | SERPINA3 | -6.0 | -3.0 | -4.2 |
| 203207_s_at | mitochondrial fission regulator 1 | MTFR1 | -5.7 | -3.2 | -4.2 |
| 204608_at | argininosuccinate lyase | ASL | -8.6 | -2.0 | -4.2 |
| 212364_at | myosin IB | MYO1B | -5.8 | -3.1 | -4.2 |
| 212623_at | transmembrane protein 41B | TMEM41B | -4.9 | -3.7 | -4.2 |
| 218701_at | lactamase, beta 2 | LACTB2 | -8.5 | -2.1 | -4.2 |
| 223319_at | gephyrin | GPHN | -5.4 | -3.3 | -4.2 |
| 231669_at | Selenoprotein P, plasma, 1 | SEPP1 | -4.2 | -4.3 | -4.2 |
| 1555274_a_at | ethanolaminephosphotransferase 1 (CDP-ethanolamine-specific) | EPT1 | -6.9 | -2.4 | -4.1 |
| 1559363_at | hypothetical protein LOC283587 | LOC283587 | -6.5 | -2.6 | -4.1 |
| 200637_s_at | protein tyrosine phosphatase, receptor type, F | PTPRF | -5.3 | -3.1 | -4.1 |
| 202539_s_at | 3-hydroxy-3-methylglutaryl-CoA reductase | HMGCR | -4.3 | -4.0 | -4.1 |
| 214413_at | Tyrosine aminotransferase | TAT | -7.1 | -2.4 | -4.1 |
| 218692_at | syntabulin (syntaxin-interacting) | SYBU | -4.8 | -3.6 | -4.1 |
| 218923_at | chitobiase, di-N-acetyl- | CTBS | -4.2 | -4.0 | -4.1 |
| 218983_at | complement component 1, r subcomponent-like | C1RL | -4.6 | -3.7 | -4.1 |
| 224990_at | chromosome 4 open reading frame 34 | C4orf34 | -5.4 | -3.1 | -4.1 |
| 226021_at | retinol dehydrogenase 10 (all-trans) | RDH10 | -6.1 | -2.8 | -4.1 |
| 229376_at | prospero homeobox 1 | PROX1 | -6.9 | -2.5 | -4.1 |
| 231751_at | ATP-binding cassette, sub-family G (WHITE), member 8 | ABCG8 | -6.6 | -2.5 | -4.1 |
| 231944_at | ERO1-like beta (S. cerevisiae) | ERO1LB | -4.2 | -3.9 | -4.1 |
| 234103_at | Potassium channel, subfamily T, member 2 | KCNT2 | -3.9 | -4.4 | -4.1 |
| 236217_at | solute carrier family 31 (copper transporters), member 1 | SLC31A1 | -5.1 | -3.3 | -4.1 |
| 238067_at | TBC1 domain family, member 8B (with GRAM domain) | TBC1D8B | -4.1 | -4.0 | -4.1 |
| 238647_at | chromosome 14 open reading frame 28 | C14orf28 | -3.9 | -4.2 | -4.1 |
| 239989_at | centlein, centrosomal protein | CNTLN | -4.1 | -4.2 | -4.1 |
| 1555905_a_at | chromosome 3 open reading frame 23 | C3orf23 | -4.8 | -3.4 | -4.0 |
| 200862_at | 24-dehydrocholesterol reductase | DHCR24 | -6.1 | -2.6 | -4.0 |
| 202308_at | sterol regulatory element binding transcription factor 1 | SREBF1 | -4.2 | -3.7 | -4.0 |
| 203646_at | ferredoxin 1 | FDX1 | -5.2 | -3.1 | -4.0 |
| 204072_s_at | furry homolog (Drosophila) | FRY | -4.5 | -3.6 | -4.0 |
| 206252_s_at | arginine vasopressin receptor 1A | AVPR1A | -4.2 | -3.8 | -4.0 |
| 212812_at | serine incorporator 5 | SERINC5 | -5.4 | -3.0 | -4.0 |
| 213353_at | ATP-binding cassette, sub-family A (ABC1), member 5 | ABCA5 | -7.0 | -2.3 | -4.0 |
| 221563_at | dual specificity phosphatase 10 | DUSP10 | -4.2 | -3.9 | -4.0 |
| 225817_at | cingulin-like 1 | CGNL1 | -4.5 | -3.5 | -4.0 |
| 226390_at | StAR-related lipid transfer (START) domain containing 4 | STARD4 | -3.8 | -4.2 | -4.0 |
| 228561_at | cell division cycle 37 homolog (S. cerevisiae)-like 1 | CDC37L1 | -4.3 | -3.6 | -4.0 |
| 228855_at | nudix (nucleoside diphosphate linked moiety X)-type motif 7 | NUDT7 | -7.9 | -2.1 | -4.0 |
| 229241_at | lactate dehydrogenase D | LDHD | -7.7 | -2.1 | -4.0 |
| 231102_at | carnitine O-octanoyltransferase | CROT | -5.9 | -2.7 | -4.0 |
| 235099_at | CKLF-like MARVEL transmembrane domain containing 8 | CMTM8 | -4.5 | -3.5 | -4.0 |
| 235766_x_at | RAB27A, member RAS oncogene family | RAB27A | -4.5 | -3.5 | -4.0 |
| 242702_at | methylmalonic aciduria (cobalamin deficiency) cblA type | MMAA | -5.6 | -2.8 | -4.0 |
| 203986_at | family with sequence similarity 47, member E starch binding domain 1 | FAM47E STBD1 | -6.3 | -2.5 | -3.9 |
| 206058_at | solute carrier family 6 (neurotransmitter transporter, betaine/GABA), member 12 | SLC6A12 | -5.8 | -2.6 | -3.9 |
| 206417_at | cyclic nucleotide gated channel alpha 1 | CNGA1 | -3.8 | -4.0 | -3.9 |
| 208034_s_at | protein Z, vitamin K-dependent plasma glycoprotein | PROZ | -6.6 | -2.3 | -3.9 |
| 210614_at | tocopherol (alpha) transfer protein | TTPA | -5.0 | -3.1 | -3.9 |
| 213629_x_at | metallothionein 1F | MT1F | -6.4 | -2.4 | -3.9 |
| 215933_s_at | hematopoietically expressed homeobox | HHEX | -3.7 | -4.2 | -3.9 |
| 218552_at | enoyl CoA hydratase domain containing 2 | ECHDC2 | -5.0 | -3.1 | -3.9 |
| 218931_at | RAB17, member RAS oncogene family | RAB17 | -5.6 | -2.7 | -3.9 |
| 221268_s_at | sphingosine-1-phosphate phosphatase 1 | SGPP1 | -3.7 | -4.2 | -3.9 |
| 221868_at | poly(A) binding protein interacting protein 2B | PAIP2B | -4.8 | -3.2 | -3.9 |
| 222128_at | NOP2/Sun domain family, member 6 | NSUN6 | -5.2 | -2.9 | -3.9 |
| 223652_at | arsenic (+3 oxidation state) methyltransferase | AS3MT | -6.0 | -2.5 | -3.9 |
| 224327_s_at | diacylglycerol O-acyltransferase 2 | DGAT2 | -8.2 | -1.8 | -3.9 |
| 229222_at | acyl-CoA synthetase short-chain family member 3 | ACSS3 | -5.9 | -2.5 | -3.9 |
| 229498_at | muscleblind-like 3 (Drosophila) | MBNL3 | -5.0 | -3.0 | -3.9 |
| 201790_s_at | 7-dehydrocholesterol reductase | DHCR7 | -6.7 | -2.1 | -3.8 |
| 202441_at | ER lipid raft associated 1 | ERLIN1 | -5.5 | -2.6 | -3.8 |
| 202960_s_at | methylmalonyl CoA mutase | MUT | -5.2 | -2.8 | -3.8 |
| 204462_s_at | solute carrier family 16, member 2 (monocarboxylic acid transporter 8) | SLC16A2 | -4.3 | -3.4 | -3.8 |
| 205225_at | estrogen receptor 1 | ESR1 | -3.3 | -4.5 | -3.8 |
| 205620_at | coagulation factor X | F10 | -7.1 | -2.1 | -3.8 |
| 205972_at | solute carrier family 38, member 3 | SLC38A3 | -4.8 | -3.0 | -3.8 |
| 207392_x_at | UDP glucuronosyltransferase 2 family, polypeptide B15 | UGT2B15 | -6.2 | -2.4 | -3.8 |
| 207421_at | carbonic anhydrase VA, mitochondrial | CA5A | -9.4 | -1.5 | -3.8 |
| 210904_s_at | interleukin 13 receptor, alpha 1 | IL13RA1 | -4.1 | -3.5 | -3.8 |
| 218024_at | brain protein 44-like | BRP44L | -5.6 | -2.5 | -3.8 |
| 218345_at | transmembrane protein 176A | TMEM176A | -6.8 | -2.1 | -3.8 |
| 219268_at | ethanolamine kinase 2 | ETNK2 | -4.3 | -3.3 | -3.8 |
| 220390_at | ATP/GTP binding protein-like 2 | AGBL2 | -3.5 | -4.0 | -3.8 |
| 223839_s_at | stearoyl-CoA desaturase (delta-9-desaturase) | SCD | -5.4 | -2.8 | -3.8 |
| 226388_at | transcription elongation factor A (SII), 3 | TCEA3 | -5.6 | -2.5 | -3.8 |
| 231955_s_at | 3-hydroxyisobutyrate dehydrogenase | HIBADH | -5.4 | -2.6 | -3.8 |
| 235093_at | peroxisomal biogenesis factor 13 | PEX13 | -4.6 | -3.2 | -3.8 |
| 239345_at | solute carrier family 19, member 3 | SLC19A3 | -4.9 | -3.0 | -3.8 |
| 239960_x_at | Lyrm7 homolog (mouse) | LYRM7 | -3.6 | -4.1 | -3.8 |
| 202454_s_at | v-erb-b2 erythroblastic leukemia viral oncogene homolog 3 (avian) | ERBB3 | -4.1 | -3.3 | -3.7 |
| 204485_s_at | target of myb1 (chicken)-like 1 | TOM1L1 | -4.7 | -3.0 | -3.7 |
| 204745_x_at | metallothionein 1G | MT1G | -7.9 | -1.8 | -3.7 |
| 205894_at | arylsulfatase E (chondrodysplasia punctata 1) | ARSE | -6.1 | -2.2 | -3.7 |
| 208369_s_at | glutaryl-CoA dehydrogenase | GCDH | -5.5 | -2.4 | -3.7 |
| 210665_at | tissue factor pathway inhibitor (lipoprotein-associated coagulation inhibitor) | TFPI | -4.6 | -3.0 | -3.7 |
| 211489_at | adrenergic, alpha-1A-, receptor | ADRA1A | -3.7 | -3.7 | -3.7 |
| 212245_at | multiple coagulation factor deficiency 2 | MCFD2 | -4.5 | -3.0 | -3.7 |
| 212611_at | deltex homolog 4 (Drosophila) | DTX4 | -4.4 | -3.2 | -3.7 |
| 213282_at | apolipoprotein O-like | APOOL | -4.1 | -3.3 | -3.7 |
| 214142_at | zymogen granule protein 16 homolog (rat) | ZG16 | -4.8 | -2.8 | -3.7 |
| 219664_s_at | 2,4-dienoyl CoA reductase 2, peroxisomal | DECR2 | -5.7 | -2.4 | -3.7 |
| 219732_at | lipid phosphate phosphatase-related protein type 1 | LPPR1 | -3.3 | -4.0 | -3.7 |
| 222666_s_at | RNA terminal phosphate cyclase-like 1 | RCL1 | -6.8 | -2.0 | -3.7 |
| 222805_at | mannosidase, endo-alpha | MANEA | -3.8 | -3.5 | -3.7 |
| 225747_at | coenzyme Q10 homolog A (S. cerevisiae) | COQ10A | -3.6 | -3.8 | -3.7 |
| 229116_at | connector enhancer of kinase suppressor of Ras 2 | CNKSR2 | -3.7 | -3.7 | -3.7 |
| 229994_at | Nuclear factor I/A | NFIA | -3.2 | -4.2 | -3.7 |
| 230143_at | ring finger protein 165 | RNF165 | -4.3 | -3.1 | -3.7 |
| 230659_at | ER degradation enhancer, mannosidase alpha-like 1 | EDEM1 | -4.1 | -3.3 | -3.7 |
| 231483_at | hypothetical LOC100509390 | LOC100509390 | -4.7 | -2.9 | -3.7 |
| 235509_at | chromosome 8 open reading frame 38 | C8orf38 | -4.9 | -2.8 | -3.7 |
| 235747_at | Solute carrier family 25 (mitochondrial carrier; Graves disease autoantigen), member 16 | SLC25A16 | -3.9 | -3.5 | -3.7 |
| 201425_at | aldehyde dehydrogenase 2 family (mitochondrial) | ALDH2 | -3.8 | -3.5 | -3.6 |
| 203335_at | phytanoyl-CoA 2-hydroxylase | PHYH | -5.7 | -2.3 | -3.6 |
| 203979_at | cytochrome P450, family 27, subfamily A, polypeptide 1 | CYP27A1 | -6.3 | -2.0 | -3.6 |
| 206010_at | hyaluronan binding protein 2 | HABP2 | -6.5 | -2.0 | -3.6 |
| 210683_at | neurturin | NRTN | -4.1 | -3.2 | -3.6 |
| 213385_at | chimerin (chimaerin) 2 | CHN2 | -4.1 | -3.3 | -3.6 |
| 215712_s_at | insulin-like growth factor binding protein, acid labile subunit | IGFALS | -4.3 | -3.0 | -3.6 |
| 217776_at | retinol dehydrogenase 11 (all-trans/9-cis/11-cis) | RDH11 | -3.7 | -3.4 | -3.6 |
| 221679_s_at | abhydrolase domain containing 6 | ABHD6 | -4.7 | -2.7 | -3.6 |
| 222212_s_at | LAG1 homolog, ceramide synthase 2 | LASS2 | -4.9 | -2.6 | -3.6 |
| 222400_s_at | acireductone dioxygenase 1 | ADI1 | -5.5 | -2.4 | -3.6 |
| 223593_at | aminoadipate aminotransferase | AADAT | -4.2 | -3.1 | -3.6 |
| 224361_s_at | interleukin 17 receptor B | IL17RB | -6.2 | -2.1 | -3.6 |
| 225455_at | transcriptional adaptor 1 | TADA1 | -3.6 | -3.7 | -3.6 |
| 226612_at | ubiquitin-conjugating enzyme E2Q family-like 1 | UBE2QL1 | -4.2 | -3.0 | -3.6 |
| 227094_at | dehydrogenase E1 and transketolase domain containing 1 | DHTKD1 | -6.1 | -2.2 | -3.6 |
| 227140_at | inhibin, beta A | INHBA | -3.3 | -4.0 | -3.6 |
| 227425_at | RALBP1 associated Eps domain containing 2 | REPS2 | -3.6 | -3.5 | -3.6 |
| 227752_at | serine palmitoyltransferase, long chain base subunit 3 | SPTLC3 | -5.2 | -2.5 | -3.6 |
| 228565_at | mixed lineage kinase 4 | KIAA1804 | -3.1 | -4.2 | -3.6 |
| 230265_at | Sel-1 suppressor of lin-12-like (C. elegans) | SEL1L | -3.4 | -3.9 | -3.6 |
| 231187_at | solute carrier family 28 (sodium-coupled nucleoside transporter), member 1 | SLC28A1 | -6.4 | -2.0 | -3.6 |
| 235349_at | family with sequence similarity 82, member A1 | FAM82A1 | -2.9 | -4.4 | -3.6 |
| 235350_at | chromosome 4 open reading frame 19 | C4orf19 | -4.4 | -2.9 | -3.6 |
| 243495_s_at | --- | --- | -2.8 | -4.6 | -3.6 |
| 244151_at | hypothetical LOC285733 | LOC285733 | -5.2 | -2.4 | -3.6 |
| 1558279_a_at | 3-ketodihydrosphingosine reductase | KDSR | -3.4 | -3.6 | -3.5 |
| 202409_at | insulin-like growth factor 2 (somatomedin A)  INS-IGF2 readthrough transcript | IGF2  INS-IGF2 | -3.1 | -3.9 | -3.5 |
| 202793_at | lysophosphatidylcholine acyltransferase 3 | LPCAT3 | -4.7 | -2.7 | -3.5 |
| 204039_at | CCAAT/enhancer binding protein (C/EBP), alpha | CEBPA | -5.6 | -2.2 | -3.5 |
| 204845_s_at | glutamyl aminopeptidase (aminopeptidase A) | ENPEP | -4.4 | -2.8 | -3.5 |
| 204973_at | gap junction protein, beta 1, 32kDa | GJB1 | -5.2 | -2.3 | -3.5 |
| 209623_at | methylcrotonoyl-CoA carboxylase 2 (beta) | MCCC2 | -5.1 | -2.5 | -3.5 |
| 216607_s_at | cytochrome P450, family 51, subfamily A, polypeptide 1 | CYP51A1 | -4.1 | -2.9 | -3.5 |
| 218605_at | transcription factor B2, mitochondrial | TFB2M | -4.7 | -2.6 | -3.5 |
| 222784_at | SPARC related modular calcium binding 1 | SMOC1 | -4.2 | -3.0 | -3.5 |
| 223266_at | STE20-related kinase adaptor beta | STRADB | -4.6 | -2.6 | -3.5 |
| 224657_at | ERBB receptor feedback inhibitor 1 | ERRFI1 | -6.0 | -2.0 | -3.5 |
| 225062_at | hypothetical LOC389831 | LOC389831 | -2.5 | -5.1 | -3.5 |
| 226225_at | mutated in colorectal cancers | MCC | -3.1 | -4.0 | -3.5 |
| 227019_at | chromosome 1 open reading frame 226 | C1orf226 | -4.1 | -2.9 | -3.5 |
| 229526_at | aquaporin 11 | AQP11 | -6.0 | -2.0 | -3.5 |
| 230364_at | choline phosphotransferase 1 | CHPT1 | -3.5 | -3.5 | -3.5 |
| 231174_s_at | erythrocyte membrane protein band 4.1-like 2* | EPB41L2 | -3.4 | -3.7 | -3.5 |
| 232271_at | hepatocyte nuclear factor 4, gamma | HNF4G | -4.2 | -2.9 | -3.5 |
| 243681_at | SH3 and multiple ankyrin repeat domains 2 | SHANK2 | -3.1 | -3.9 | -3.5 |
| 59697_at | RAB15, member RAS onocogene family | RAB15 | -4.1 | -3.0 | -3.5 |
| 203970_s_at | peroxisomal biogenesis factor 3 | PEX3 | -3.9 | -2.9 | -3.4 |
| 204541_at | SEC14-like 2 (S. cerevisiae) | SEC14L2 | -4.8 | -2.4 | -3.4 |
| 205307_s_at | kynurenine 3-monooxygenase (kynurenine 3-hydroxylase) | KMO | -4.2 | -2.8 | -3.4 |
| 206656_s_at | chromosome 20 open reading frame 3 | C20orf3 | -4.3 | -2.7 | -3.4 |
| 209123_at | quinoid dihydropteridine reductase | QDPR | -5.4 | -2.2 | -3.4 |
| 209512_at | hydroxysteroid dehydrogenase like 2 | HSDL2 | -5.8 | -2.0 | -3.4 |
| 210830_s_at | paraoxonase 2 | PON2 | -6.0 | -1.9 | -3.4 |
| 211774_s_at | methylmalonic aciduria (cobalamin deficiency) cblC type, with homocystinuria | MMACHC | -3.8 | -2.9 | -3.4 |
| 220070_at | jumonji domain containing 5 | JMJD5 | -4.4 | -2.5 | -3.4 |
| 222403_at | mitochondrial carrier homolog 2 (C. elegans) | MTCH2 | -4.9 | -2.3 | -3.4 |
| 226121_at | dehydrogenase/reductase (SDR family) member 13 | DHRS13 | -3.6 | -3.3 | -3.4 |
| 226859_at | DnaJ (Hsp40) homolog, subfamily C , member 25 | DNAJC25 | -4.0 | -2.9 | -3.4 |
| 227492_at | occludin | OCLN | -3.4 | -3.4 | -3.4 |
| 231008_at | unc-5 homolog C (C. elegans)-like | UNC5CL | -4.2 | -2.8 | -3.4 |
| 241368_at | perilipin 5 | PLIN5 | -4.7 | -2.5 | -3.4 |
| 241741_at | cardiolipin synthase 1 | CRLS1 | -4.2 | -2.8 | -3.4 |
| 202890_at | microtubule-associated protein 7 | MAP7 | -4.1 | -2.6 | -3.3 |
| 202922_at | glutamate-cysteine ligase, catalytic subunit | GCLC | -3.8 | -2.9 | -3.3 |
| 203433_at | 5,10-methenyltetrahydrofolate synthetase (5-formyltetrahydrofolate cyclo-ligase) | MTHFS | -4.0 | -2.8 | -3.3 |
| 204615_x_at | isopentenyl-diphosphate delta isomerase 1 | IDI1 | -3.5 | -3.2 | -3.3 |
| 205651_x_at | Rap guanine nucleotide exchange factor (GEF) 4 | RAPGEF4 | -4.4 | -2.5 | -3.3 |
| 209496_at | retinoic acid receptor responder (tazarotene induced) 2 | RARRES2 | -3.9 | -2.8 | -3.3 |
| 210063_at | sarcosine dehydrogenase | SARDH | -4.2 | -2.5 | -3.3 |
| 214658_at | transmembrane emp24 protein transport domain containing 7 | TMED7 | -4.0 | -2.8 | -3.3 |
| 218025_s_at | peroxisomal D3,D2-enoyl-CoA isomerase | PECI | -5.8 | -1.9 | -3.3 |
| 219263_at | ring finger protein 128 | RNF128 | -4.8 | -2.3 | -3.3 |
| 219796_s_at | cadherin-related family member 5 | CDHR5 | -5.2 | -2.0 | -3.3 |
| 226863_at | family with sequence similarity 110, member C | FAM110C | -3.7 | -2.9 | -3.3 |
| 228150_at | SEC16 homolog B (S. cerevisiae) | SEC16B | -6.0 | -1.9 | -3.3 |
| 229456_s_at | Dimethylarginine dimethylaminohydrolase 1 | DDAH1 | -3.7 | -2.9 | -3.3 |
| 242263_at | transmembrane emp24 protein transport domain containing 5 | TMED5 | -4.7 | -2.4 | -3.3 |
| 242931_at | LON peptidase N-terminal domain and ring finger 3 | LONRF3 | -4.6 | -2.4 | -3.3 |
| 1569940_at | Solute carrier family 6, member 16 | SLC6A16 | -2.7 | -3.7 | -3.2 |
| 201619_at | peroxiredoxin 3 | PRDX3 | -4.3 | -2.4 | -3.2 |
| 202309_at | methylenetetrahydrofolate dehydrogenase (NADP+ dependent) 1, methenyltetrahydrofolate cyclohydrolase, formyltetrahydrofolate synthetase | MTHFD1 | -5.5 | -1.9 | -3.2 |
| 202670_at | mitogen-activated protein kinase kinase 1 | MAP2K1 | -3.4 | -3.0 | -3.2 |
| 203474_at | IQ motif containing GTPase activating protein 2 | IQGAP2 | -3.6 | -2.9 | -3.2 |
| 204326_x_at | metallothionein 1X | MT1X | -5.8 | -1.8 | -3.2 |
| 204427_s_at | transmembrane emp24 domain trafficking protein 2 | TMED2 | -3.5 | -2.9 | -3.2 |
| 210565_at | glucagon receptor | GCGR | -3.9 | -2.6 | -3.2 |
| 210596_at | magnesium transporter 1 | MAGT1 | -3.1 | -3.2 | -3.2 |
| 210619_s_at | hyaluronoglucosaminidase 1 | HYAL1 | -5.4 | -1.9 | -3.2 |
| 211569_s_at | hydroxyacyl-CoA dehydrogenase | HADH | -4.5 | -2.3 | -3.2 |
| 212830_at | multiple EGF-like-domains 9 | MEGF9 | -4.2 | -2.4 | -3.2 |
| 212923_s_at | chromosome 6 open reading frame 145 | C6orf145 | -4.4 | -2.3 | -3.2 |
| 218772_x_at | transmembrane protein 38B | TMEM38B | -4.7 | -2.2 | -3.2 |
| 219584_at | phospholipase A1 member A | PLA1A | -6.7 | -1.5 | -3.2 |
| 220751_s_at | chromosome 5 open reading frame 4 | C5orf4 | -2.7 | -3.9 | -3.2 |
| 221014_s_at | RAB33B, member RAS oncogene family | RAB33B | -3.3 | -3.1 | -3.2 |
| 223159_s_at | NIMA (never in mitosis gene a)-related kinase 6 | NEK6 | -4.3 | -2.3 | -3.2 |
| 223233_s_at | cingulin | CGN | -3.6 | -2.8 | -3.2 |
| 223306_at | emopamil binding protein-like | EBPL | -3.6 | -2.9 | -3.2 |
| 223611_s_at | ligand of numb-protein X 1 | LNX1 | -3.4 | -3.0 | -3.2 |
| 223665_at | actin related protein M1 | ARPM1 | -3.4 | -3.0 | -3.2 |
| 225826_at | methylmalonic aciduria (cobalamin deficiency) cblB type | MMAB | -4.2 | -2.5 | -3.2 |
| 226261_at | zinc and ring finger 2 | ZNRF2 | -3.2 | -3.3 | -3.2 |
| 227569_at | ligand of numb-protein X 2 | LNX2 | -4.1 | -2.5 | -3.2 |
| 227598_at | chromosome 7 open reading frame 29 | C7orf29 | -3.7 | -2.8 | -3.2 |
| 229534_at | acyl-CoA thioesterase 4 | ACOT4 | -4.6 | -2.2 | -3.2 |
| 231919_at | dihydrolipoamide branched chain transacylase E2 | DBT | -3.3 | -3.1 | -3.2 |
| 232381_s_at | dynein, axonemal, heavy chain 5 | DNAH5 | -2.8 | -3.7 | -3.2 |
| 239999_at | --- | --- | -2.3 | -4.4 | -3.2 |
| 242317_at | HIG1 hypoxia inducible domain family, member 1A | HIGD1A | -4.6 | -2.3 | -3.2 |
| 200927_s_at | RAB14, member RAS oncogene family | RAB14 | -3.3 | -2.9 | -3.1 |
| 201984_s_at | epidermal growth factor receptor | EGFR | -4.0 | -2.3 | -3.1 |
| 202982_s_at | acyl-CoA thioesterase 1  acyl-CoA thioesterase 2 | ACOT1 ACOT2 | -4.5 | -2.1 | -3.1 |
| 203165_s_at | solute carrier family 33 (acetyl-CoA transporter), member 1 | SLC33A1 | -3.5 | -2.8 | -3.1 |
| 203293_s_at | lectin, mannose-binding, 1 | LMAN1 | -3.2 | -3.0 | -3.1 |
| 204301_at | kelch repeat and BTB (POZ) domain containing 11 | KBTBD11 | -3.7 | -2.6 | -3.1 |
| 204686_at | insulin receptor substrate 1 | IRS1 | -3.7 | -2.7 | -3.1 |
| 205353_s_at | phosphatidylethanolamine binding protein 1 | PEBP1 | -5.1 | -1.9 | -3.1 |
| 205512_s_at | apoptosis-inducing factor, mitochondrion-associated, 1 | AIFM1 | -5.2 | -1.9 | -3.1 |
| 208658_at | protein disulfide isomerase family A, member 4 | PDIA4 | -3.4 | -2.8 | -3.1 |
| 208951_at | aldehyde dehydrogenase 7 family, member A1 | ALDH7A1 | -3.9 | -2.5 | -3.1 |
| 210625_s_at | A kinase (PRKA) anchor protein 1 | AKAP1 | -3.7 | -2.6 | -3.1 |
| 212277_at | myotubularin related protein 4 | MTMR4 | -3.1 | -3.0 | -3.1 |
| 212310_at | melanoma inhibitory activity family, member 3 | MIA3 | -3.1 | -3.2 | -3.1 |
| 213626_at | carbonyl reductase 4 | CBR4 | -2.9 | -3.4 | -3.1 |
| 213874_at | serpin peptidase inhibitor, clade A (alpha-1 antiproteinase, antitrypsin), member 4 | SERPINA4 | -4.1 | -2.3 | -3.1 |
| 214136_at | nudix (nucleoside diphosphate linked moiety X)-type motif 13 | NUDT13 | -3.2 | -3.0 | -3.1 |
| 214331_at | Ts translation elongation factor, mitochondrial | TSFM | -3.2 | -2.9 | -3.1 |
| 221045_s_at | period homolog 3 (Drosophila) | PER3 | -2.5 | -3.9 | -3.1 |
| 221803_s_at | nuclear receptor binding factor 2 | NRBF2 | -3.7 | -2.6 | -3.1 |
| 223268_at | chromosome 11 open reading frame 54 | C11orf54 | -3.1 | -3.1 | -3.1 |
| 223535_at | nudix (nucleoside diphosphate linked moiety X)-type motif 12 | NUDT12 | -3.8 | -2.6 | -3.1 |
| 225158_at | G elongation factor, mitochondrial 1 | GFM1 | -4.6 | -2.1 | -3.1 |
| 225539_at | zinc finger protein 295 | ZNF295 | -3.4 | -2.8 | -3.1 |
| 225745_at | low density lipoprotein receptor-related protein 6 | LRP6 | -4.1 | -2.3 | -3.1 |
| 226003_at | kinesin family member 21A | KIF21A | -4.1 | -2.4 | -3.1 |
| 227113_at | alcohol dehydrogenase, iron containing, 1 | ADHFE1 | -3.1 | -3.1 | -3.1 |
| 227982_at | Sep (O-phosphoserine) tRNA:Sec (selenocysteine) tRNA synthase | SEPSECS | -3.4 | -2.8 | -3.1 |
| 228391_at | cytochrome P450, family 4, subfamily V, polypeptide 2 | CYP4V2 | -3.1 | -3.1 | -3.1 |
| 229829_at | chromosome 18 open reading frame 18 | C18orf18 | -3.9 | -2.4 | -3.1 |
| 231034_s_at | KIAA1357 protein* | KIAA1357 | -3.1 | -3.0 | -3.1 |
| 231625_at | solute carrier family 22 (organic anion transporter), member 9 | SLC22A9 | -4.9 | -2.0 | -3.1 |
| 244261_at | interleukin 28 receptor, alpha (interferon, lambda receptor) | IL28RA | -3.4 | -2.8 | -3.1 |
| 39248_at | aquaporin 3 (Gill blood group) | AQP3 | -4.5 | -2.1 | -3.1 |
| 1553994_at | 5'-nucleotidase, ecto (CD73) | NT5E | -4.2 | -2.1 | -3.0 |
| 1555886_at | prenyl (decaprenyl) diphosphate synthase, subunit 2 | PDSS2 | -3.8 | -2.4 | -3.0 |
| 201017_at | eukaryotic translation initiation factor 1A, X-linked | EIF1AX | -3.1 | -2.9 | -3.0 |
| 202366_at | acyl-CoA dehydrogenase, C-2 to C-3 short chain | ACADS | -3.6 | -2.5 | -3.0 |
| 202460_s_at | lipin 2 | LPIN2 | -3.4 | -2.7 | -3.0 |
| 202722_s_at | glutamine--fructose-6-phosphate transaminase 1 | GFPT1 | -4.4 | -2.1 | -3.0 |
| 203510_at | met proto-oncogene (hepatocyte growth factor receptor) | MET | -4.4 | -2.0 | -3.0 |
| 203857_s_at | protein disulfide isomerase family A, member 5 | PDIA5 | -3.3 | -2.6 | -3.0 |
| 208886_at | H1 histone family, member 0 | H1F0 | -3.2 | -2.7 | -3.0 |
| 209185_s_at | insulin receptor substrate 2 | IRS2 | -3.9 | -2.3 | -3.0 |
| 209585_s_at | multiple inositol-polyphosphate phosphatase 1 | MINPP1 | -3.7 | -2.4 | -3.0 |
| 210678_s_at | 1-acylglycerol-3-phosphate O-acyltransferase 2 (lysophosphatidic acid acyltransferase, beta) | AGPAT2 | -3.9 | -2.2 | -3.0 |
| 212694_s_at | propionyl CoA carboxylase, beta polypeptide | PCCB | -5.0 | -1.8 | -3.0 |
| 213393_at | major facilitator superfamily domain containing 9 | MFSD9 | -3.2 | -2.9 | -3.0 |
| 217047_s_at | family with sequence similarity 13, member A | FAM13A | -2.8 | -3.3 | -3.0 |
| 217289_s_at | solute carrier family 37 (glucose-6-phosphate transporter), member 4 | SLC37A4 | -3.5 | -2.6 | -3.0 |
| 218865_at | MOCO sulphurase C-terminal domain containing 1 | MOSC1 | -3.7 | -2.4 | -3.0 |
| 219164_s_at | ATG2 autophagy related 2 homolog B (S. cerevisiae) | ATG2B | -3.1 | -3.0 | -3.0 |
| 222270_at | SMEK homolog 2, suppressor of mek1 (Dictyostelium) | SMEK2 | -3.4 | -2.7 | -3.0 |
| 223099_s_at | lon peptidase 2, peroxisomal | LONP2 | -4.1 | -2.3 | -3.0 |
| 225368_at | homeodomain interacting protein kinase 2 | HIPK2 | -3.1 | -2.9 | -3.0 |
| 226181_at | tubulin, epsilon 1 | TUBE1 | -3.1 | -2.9 | -3.0 |
| 229453_at | protein disulfide isomerase family A, member 3 | PDIA3 | -3.1 | -2.9 | -3.0 |
| 229770_at | glycosyltransferase 1 domain containing 1 | GLT1D1 | -3.6 | -2.5 | -3.0 |
| 235737_at | thymic stromal lymphopoietin | TSLP | -2.7 | -3.4 | -3.0 |
| 241359_at | TLC domain containing 2 | TLCD2 | -4.0 | -2.2 | -3.0 |
| 244398_x_at | zinc finger protein 684 | ZNF684 | -3.6 | -2.5 | -3.0 |
| 1556606_at | neuron navigator 2 | NAV2 | -2.8 | -3.1 | -2.9 |
| 202245_at | lanosterol synthase (2,3-oxidosqualene-lanosterol cyclase) | LSS | -2.8 | -3.0 | -2.9 |
| 203011_at | inositol(myo)-1(or 4)-monophosphatase 1 | IMPA1 | -3.1 | -2.7 | -2.9 |
| 203059_s_at | 3'-phosphoadenosine 5'-phosphosulfate synthase 2 | PAPSS2 | -3.8 | -2.3 | -2.9 |
| 203282_at | glucan (1,4-alpha-), branching enzyme 1 | GBE1 | -4.8 | -1.8 | -2.9 |
| 203775_at | solute carrier family 25, member 13 (citrin) | SLC25A13 | -5.8 | -1.5 | -2.9 |
| 203786_s_at | tumor protein D52-like 1 | TPD52L1 | -5.9 | -1.5 | -2.9 |
| 204866_at | PHD finger protein 16 | PHF16 | -3.8 | -2.3 | -2.9 |
| 206461_x_at | metallothionein 1H | MT1H | -5.4 | -1.5 | -2.9 |
| 207791_s_at | RAB1A, member RAS oncogene family | RAB1A | -3.3 | -2.5 | -2.9 |
| 210046_s_at | isocitrate dehydrogenase 2 (NADP+), mitochondrial | IDH2 | -3.2 | -2.6 | -2.9 |
| 212282_at | transmembrane protein 97 | TMEM97 | -3.0 | -2.9 | -2.9 |
| 212703_at | talin 2 | TLN2 | -3.4 | -2.5 | -2.9 |
| 213006_at | CCAAT/enhancer binding protein (C/EBP), delta | CEBPD | -3.4 | -2.4 | -2.9 |
| 213085_s_at | WW and C2 domain containing 1 | WWC1 | -4.1 | -2.0 | -2.9 |
| 213820_s_at | StAR-related lipid transfer (START) domain containing 5 | STARD5 | -2.9 | -2.9 | -2.9 |
| 214864_s_at | glyoxylate reductase/hydroxypyruvate reductase | GRHPR | -4.0 | -2.1 | -2.9 |
| 216209_at | hypothetical LOC400084 | LOC400084 | -3.3 | -2.6 | -2.9 |
| 217757_at | alpha-2-macroglobulin | A2M | -2.9 | -2.8 | -2.9 |
| 218174_s_at | chromosome 10 open reading frame 57 | C10orf57 | -3.9 | -2.2 | -2.9 |
| 218272_at | tetratricopeptide repeat domain 38 | TTC38 | -3.7 | -2.2 | -2.9 |
| 218392_x_at | sideroflexin 1 | SFXN1 | -4.9 | -1.7 | -2.9 |
| 218477_at | transmembrane protein 14A | TMEM14A | -4.2 | -2.0 | -2.9 |
| 218532_s_at | family with sequence similarity 134, member B | FAM134B | -3.4 | -2.5 | -2.9 |
| 218901_at | phospholipid scramblase 4 | PLSCR4 | -2.9 | -2.9 | -2.9 |
| 218922_s_at | LAG1 homolog, ceramide synthase 4 | LASS4 | -3.5 | -2.3 | -2.9 |
| 219312_s_at | zinc finger and BTB domain containing 10 | ZBTB10 | -2.9 | -2.9 | -2.9 |
| 219949_at | leucine rich repeat containing 2 | LRRC2 | -3.0 | -2.9 | -2.9 |
| 221575_at | selenocysteine lyase | SCLY | -3.7 | -2.3 | -2.9 |
| 222809_x_at | coiled-coil domain containing 85C | CCDC85C | -4.6 | -1.8 | -2.9 |
| 225278_at | protein kinase, AMP-activated, beta 2 non-catalytic subunit | PRKAB2 | -3.0 | -2.8 | -2.9 |
| 225949_at | nuclear receptor binding protein 2 | NRBP2 | -3.0 | -2.8 | -2.9 |
| 226610_at | centromere protein V | CENPV | -4.7 | -1.7 | -2.9 |
| 227446_s_at | chromosome 14 open reading frame 167 | C14orf167 | -2.9 | -3.0 | -2.9 |
| 229380_at | --- | --- | -2.8 | -3.0 | -2.9 |
| 229831_at | contactin 3 (plasmacytoma associated) | CNTN3 | -3.3 | -2.5 | -2.9 |
| 229983_at | tigger transposable element derived 2 | TIGD2 | -3.3 | -2.5 | -2.9 |
| 230051_at | chromosome 10 open reading frame 47 | C10orf47 | -4.1 | -2.1 | -2.9 |
| 230677_at | chromosome 14 open reading frame 73 | C14orf73 | -3.5 | -2.3 | -2.9 |
| 232083_at | kinesin family member 16B | KIF16B | -5.3 | -1.6 | -2.9 |
| 235749_at | UDP-glucose glycoprotein glucosyltransferase 2 | UGGT2 | -4.1 | -2.1 | -2.9 |
| 238002_at | golgi integral membrane protein 4 | GOLIM4 | -3.4 | -2.5 | -2.9 |
| 238440_at | citrate lyase beta like | CLYBL | -3.6 | -2.2 | -2.9 |
| 242001_at | Isocitrate dehydrogenase 1 (NADP+), soluble | IDH1 | -3.0 | -2.7 | -2.9 |
| 242844_at | --- | --- | -2.5 | -3.3 | -2.9 |
| 1552519_at | activin A receptor, type IC | ACVR1C | -2.9 | -2.7 | -2.8 |
| 1558508_a_at | chromosome 1 open reading frame 53 | C1orf53 | -3.8 | -2.1 | -2.8 |
| 1560679_at | hypothetical protein LOC151438 | LOC151438 | -2.7 | -2.8 | -2.8 |
| 1561908_a_at | Heparan sulfate (glucosamine) 3-O-sulfotransferase 3B1 | HS3ST3B1 | -2.9 | -2.8 | -2.8 |
| 200698_at | KDEL (Lys-Asp-Glu-Leu) endoplasmic reticulum protein retention receptor 2 | KDELR2 | -3.6 | -2.1 | -2.8 |
| 201571_s_at | dCMP deaminase | DCTD | -3.7 | -2.2 | -2.8 |
| 202003_s_at | acetyl-CoA acyltransferase 2 | ACAA2 | -5.6 | -1.4 | -2.8 |
| 202022_at | aldolase C, fructose-bisphosphate | ALDOC | -2.9 | -2.6 | -2.8 |
| 202085_at | tight junction protein 2 (zona occludens 2) | TJP2 | -3.2 | -2.5 | -2.8 |
| 202502_at | acyl-CoA dehydrogenase, C-4 to C-12 straight chain | ACADM | -3.7 | -2.1 | -2.8 |
| 203557_s_at | pterin-4 alpha-carbinolamine dehydratase/dimerization cofactor of hepatocyte nuclear factor 1 alpha | PCBD1 | -4.1 | -1.9 | -2.8 |
| 203586_s_at | ADP-ribosylation factor-like 4D | ARL4D | -2.8 | -2.8 | -2.8 |
| 204055_s_at | CTAGE family, member 5 | CTAGE5 | -2.4 | -3.3 | -2.8 |
| 204347_at | adenylate kinase 4 | AK4 | -6.2 | -1.3 | -2.8 |
| 206020_at | suppressor of cytokine signaling 6 | SOCS6 | -3.2 | -2.5 | -2.8 |
| 207386_at | cytochrome P450, family 7, subfamily B, polypeptide 1 | CYP7B1 | -3.4 | -2.3 | -2.8 |
| 209262_s_at | nuclear receptor subfamily 2, group F, member 6 | NR2F6 | -4.0 | -2.0 | -2.8 |
| 212067_s_at | complement component 1, r subcomponent | C1R | -2.0 | -4.0 | -2.8 |
| 212402_at | zinc finger CCCH-type containing 13 | ZC3H13 | -2.8 | -2.8 | -2.8 |
| 212459_x_at | succinate-CoA ligase, GDP-forming, beta subunit | SUCLG2 | -3.5 | -2.2 | -2.8 |
| 213133_s_at | glycine cleavage system protein H (aminomethyl carrier)  glycine cleavage system protein H pseudogene | GCSH  LOC100329108 | -5.8 | -1.3 | -2.8 |
| 213306_at | multiple PDZ domain protein | MPDZ | -3.0 | -2.6 | -2.8 |
| 218036_x_at | NMD3 homolog (S. cerevisiae) | NMD3 | -3.5 | -2.3 | -2.8 |
| 218504_at | fumarylacetoacetate hydrolase domain containing 2A | FAHD2A | -3.7 | -2.2 | -2.8 |
| 218676_s_at | phosphatidylcholine transfer protein | PCTP | -3.8 | -2.1 | -2.8 |
| 218877_s_at | tRNA methyltransferase 11 homolog (S. cerevisiae) | TRMT11 | -3.5 | -2.3 | -2.8 |
| 219298_at | enoyl CoA hydratase domain containing 3 | ECHDC3 | -5.6 | -1.4 | -2.8 |
| 220084_at | chromosome 14 open reading frame 105 | C14orf105 | -2.8 | -2.8 | -2.8 |
| 221142_s_at | peroxisomal trans-2-enoyl-CoA reductase | PECR | -4.6 | -1.8 | -2.8 |
| 223324_s_at | transient receptor potential cation channel, subfamily M, member 7 | TRPM7 | -2.8 | -2.9 | -2.8 |
| 225632_s_at | ISY1 splicing factor homolog (S. cerevisiae) RAB43, member RAS oncogene family | ISY1  RAB43 | -3.2 | -2.4 | -2.8 |
| 226238_at | methylmalonyl CoA epimerase | MCEE | -2.6 | -3.0 | -2.8 |
| 226589_at | transmembrane protein 192 | TMEM192 | -3.0 | -2.7 | -2.8 |
| 227559_at | NADH dehydrogenase (ubiquinone) 1 alpha subcomplex, assembly factor 4 | NDUFAF4 | -2.8 | -2.8 | -2.8 |
| 227962_at | acyl-CoA oxidase 1, palmitoyl | ACOX1 | -5.1 | -1.6 | -2.8 |
| 230300_at | --- | --- | -2.6 | -2.9 | -2.8 |
| 230624_at | solute carrier family 25, member 27 | SLC25A27 | -2.0 | -3.8 | -2.8 |
| 230792_at | fatty acid amide hydrolase 2 | FAAH2 | -3.1 | -2.6 | -2.8 |
| 231169_at | interleukin 14* | IL14 | -3.0 | -2.6 | -2.8 |
| 232500_at | Ral GTPase activating protein, alpha subunit 2 (catalytic) | RALGAPA2 | -2.2 | -3.5 | -2.8 |
| 235798_at | transmembrane protein 170B | TMEM170B | -2.6 | -2.9 | -2.8 |
| 236548_at | GIPC PDZ domain containing family, member 2 | GIPC2 | -3.7 | -2.1 | -2.8 |
| 238458_at | EF-hand domain family, member A2 | EFHA2 | -3.1 | -2.5 | -2.8 |
| 242626_at | sterile alpha motif domain containing 5 | SAMD5 | -2.9 | -2.7 | -2.8 |
| 1554241_at | coagulation factor C homolog, cochlin (Limulus polyphemus) | COCH | -2.8 | -2.6 | -2.7 |
| 1555226_s_at | chromosome 1 open reading frame 43 | C1orf43 | -2.9 | -2.5 | -2.7 |
| 1556285_s_at | pyrophosphatase (inorganic) 2 | PPA2 | -3.1 | -2.2 | -2.7 |
| 201566_x_at | inhibitor of DNA binding 2, dominant negative helix-loop-helix protein | ID2 | -3.0 | -2.4 | -2.7 |
| 202772_at | 3-hydroxymethyl-3-methylglutaryl-CoA lyase | HMGCL | -3.6 | -2.0 | -2.7 |
| 203860_at | propionyl CoA carboxylase, alpha polypeptide | PCCA | -3.4 | -2.2 | -2.7 |
| 204867_at | GTP cyclohydrolase I feedback regulator | GCHFR | -4.0 | -1.8 | -2.7 |
| 207621_s_at | phosphatidylethanolamine N-methyltransferase | PEMT | -4.7 | -1.6 | -2.7 |
| 208015_at | SMAD family member 1 | SMAD1 | -2.4 | -3.0 | -2.7 |
| 209522_s_at | carnitine O-acetyltransferase | CRAT | -4.3 | -1.7 | -2.7 |
| 209681_at | solute carrier family 19 (thiamine transporter), member 2 | SLC19A2 | -3.5 | -2.1 | -2.7 |
| 210640_s_at | G protein-coupled estrogen receptor 1 | GPER | -3.3 | -2.2 | -2.7 |
| 212239_at | phosphoinositide-3-kinase, regulatory subunit 1 (alpha) | PIK3R1 | -2.4 | -3.0 | -2.7 |
| 218793_s_at | sex comb on midleg-like 1 (Drosophila) | SCML1 | -3.7 | -2.0 | -2.7 |
| 220183_s_at | nudix (nucleoside diphosphate linked moiety X)-type motif 6 | NUDT6 | -3.2 | -2.2 | -2.7 |
| 221139_s_at | cysteine sulfinic acid decarboxylase | CSAD | -3.6 | -2.0 | -2.7 |
| 223440_at | chromosome 16 open reading frame 70 | C16orf70 | -3.3 | -2.3 | -2.7 |
| 225202_at | Rho-related BTB domain containing 3 | RHOBTB3 | -3.0 | -2.5 | -2.7 |
| 225880_at | torsin A interacting protein 2 | TOR1AIP2 | -3.1 | -2.4 | -2.7 |
| 225974_at | transmembrane protein 64 | TMEM64 | -3.3 | -2.2 | -2.7 |
| 226778_at | chromosome 8 open reading frame 42 | C8orf42 | -2.8 | -2.5 | -2.7 |
| 227196_at | rhophilin, Rho GTPase binding protein 2 | RHPN2 | -3.1 | -2.4 | -2.7 |
| 227268_at | ring finger protein, transmembrane 1 | RNFT1 | -2.9 | -2.6 | -2.7 |
| 228031_at | tocopherol (alpha) transfer protein-like | TTPAL | -2.8 | -2.5 | -2.7 |
| 229313_at | anoctamin 5 | ANO5 | -3.2 | -2.4 | -2.7 |
| 229633_at | integrator complex subunit 10 | INTS10 | -2.7 | -2.8 | -2.7 |
| 229954_at | choline dehydrogenase | CHDH | -3.7 | -2.0 | -2.7 |
| 230433_at | hCG2028352-like | LOC729970 | -3.2 | -2.2 | -2.7 |
| 230543_at | ubiquitin specific peptidase 9, X-linked | USP9X | -3.3 | -2.2 | -2.7 |
| 235341_at | DnaJ (Hsp40) homolog, subfamily C, member 3 | DNAJC3 | -3.2 | -2.2 | -2.7 |
| 243008_at | --- | --- | -2.8 | -2.7 | -2.7 |
| 1554077_a_at | transmembrane protein 53 | TMEM53 | -4.2 | -1.6 | -2.6 |
| 1564053_a_at | YTH domain family, member 3 | YTHDF3 | -3.0 | -2.3 | -2.6 |
| 1568779_a_at | extracellular matrix protein 2, female organ and adipocyte specific | ECM2 | -2.8 | -2.4 | -2.6 |
| 200656_s_at | prolyl 4-hydroxylase, beta polypeptide | P4HB | -4.1 | -1.7 | -2.6 |
| 201701_s_at | progesterone receptor membrane component 2 | PGRMC2 | -2.9 | -2.3 | -2.6 |
| 203716_s_at | dipeptidyl-peptidase 4 | DPP4 | -3.8 | -1.8 | -2.6 |
| 204044_at | quinolinate phosphoribosyltransferase | QPRT | -4.2 | -1.6 | -2.6 |
| 207655_s_at | B-cell linker | BLNK | -2.1 | -3.2 | -2.6 |
| 213424_at | KIAA0895 | KIAA0895 | -2.9 | -2.4 | -2.6 |
| 218557_at | nitrilase family, member 2 | NIT2 | -4.1 | -1.7 | -2.6 |
| 218783_at | integrator complex subunit 7 | INTS7 | -2.7 | -2.5 | -2.6 |
| 220924_s_at | solute carrier family 38, member 2 | SLC38A2 | -3.7 | -1.9 | -2.6 |
| 221213_s_at | zinc finger protein 280D | ZNF280D | -2.2 | -3.0 | -2.6 |
| 222979_s_at | surfeit 4 | SURF4 | -3.8 | -1.8 | -2.6 |
| 225150_s_at | rhotekin | RTKN | -4.1 | -1.6 | -2.6 |
| 225311_at | isovaleryl-CoA dehydrogenase | IVD | -2.9 | -2.2 | -2.6 |
| 225616_at | SPRY domain containing 4 | SPRYD4 | -4.1 | -1.6 | -2.6 |
| 225661_at | interferon (alpha, beta and omega) receptor 1 | IFNAR1 | -2.5 | -2.8 | -2.6 |
| 226160_at | hexose-6-phosphate dehydrogenase (glucose 1-dehydrogenase) | H6PD | -3.5 | -1.9 | -2.6 |
| 226167_at | synaptotagmin VII | SYT7 | -2.8 | -2.4 | -2.6 |
| 226650_at | zinc finger, AN1-type domain 2A | ZFAND2A | -3.5 | -2.0 | -2.6 |
| 227629_at | prolactin receptor | PRLR | -2.2 | -3.2 | -2.6 |
| 228284_at | transducin-like enhancer of split 1 (E(sp1) homolog, Drosophila) | TLE1 | -3.5 | -1.9 | -2.6 |
| 229163_at | calcium/calmodulin-dependent protein kinase II inhibitor 1 | CAMK2N1 | -2.9 | -2.4 | -2.6 |
| 230031_at | heat shock 70kDa protein 5 (glucose-regulated protein, 78kDa) | HSPA5 | -2.9 | -2.3 | -2.6 |
| 230609_at | clathrin interactor 1 | CLINT1 | -2.4 | -2.8 | -2.6 |
| 230769_at | DENN/MADD domain containing 2C | DENND2C | -3.1 | -2.2 | -2.6 |
| 230788_at | glucosaminyl (N-acetyl) transferase 2, I-branching enzyme (I blood group) | GCNT2 | -4.1 | -1.7 | -2.6 |
| 231070_at | iodotyrosine deiodinase | IYD | -3.1 | -2.2 | -2.6 |
| 239435_x_at | shroom family member 1 | SHROOM1 | -4.2 | -1.6 | -2.6 |
| 242100_at | chondroitin sulfate synthase 3 | CHSY3 | -2.3 | -3.0 | -2.6 |
| 242852_at | hypothetical protein LOC285147 | LOC285147 | -2.2 | -3.0 | -2.6 |
| 1552281_at | solute carrier family 39 (metal ion transporter), member 5 | SLC39A5 | -4.6 | -1.3 | -2.5 |
| 1552733_at | kelch domain containing 1 | KLHDC1 | -1.8 | -3.5 | -2.5 |
| 1553465_a_at | carboxylesterase 5A | CES5A | -2.8 | -2.2 | -2.5 |
| 1559618_at | hypothetical protein LOC100129447 | LOC100129447 | -2.2 | -2.8 | -2.5 |
| 201819_at | scavenger receptor class B, member 1 | SCARB1 | -4.1 | -1.5 | -2.5 |
| 203021_at | secretory leukocyte peptidase inhibitor | SLPI | -2.2 | -2.9 | -2.5 |
| 203662_s_at | tropomodulin 1 | TMOD1 | -2.2 | -2.9 | -2.5 |
| 203803_at | prenylcysteine oxidase 1 | PCYOX1 | -2.3 | -2.6 | -2.5 |
| 204042_at | WAS protein family, member 3 | WASF3 | -2.2 | -2.7 | -2.5 |
| 204224_s_at | GTP cyclohydrolase 1 | GCH1 | -4.1 | -1.6 | -2.5 |
| 206144_at | membrane associated guanylate kinase, WW and PDZ domain containing 1 | MAGI1 | -2.3 | -2.7 | -2.5 |
| 207030_s_at | cysteine and glycine-rich protein 2 | CSRP2 | -2.1 | -3.0 | -2.5 |
| 207687_at | inhibin, beta C | INHBC | -2.7 | -2.3 | -2.5 |
| 207776_s_at | calcium channel, voltage-dependent, beta 2 subunit | CACNB2 | -2.7 | -2.4 | -2.5 |
| 211248_s_at | chordin | CHRD | -2.9 | -2.1 | -2.5 |
| 211760_s_at | vesicle-associated membrane protein 4 | VAMP4 | -2.5 | -2.5 | -2.5 |
| 212479_s_at | required for meiotic nuclear division 5 homolog A (S. cerevisiae) | RMND5A | -2.5 | -2.4 | -2.5 |
| 213374_x_at | 3-hydroxyisobutyryl-CoA hydrolase | HIBCH | -2.9 | -2.1 | -2.5 |
| 214358_at | acetyl-CoA carboxylase alpha | ACACA | -3.1 | -2.0 | -2.5 |
| 215506_s_at | DIRAS family, GTP-binding RAS-like 3 | DIRAS3 | -2.1 | -2.8 | -2.5 |
| 218254_s_at | SAR1 homolog B (S. cerevisiae) | SAR1B | -3.4 | -1.9 | -2.5 |
| 219999_at | mannosidase, alpha, class 2A, member 2 | MAN2A2 | -2.5 | -2.4 | -2.5 |
| 222317_at | phosphodiesterase 3B, cGMP-inhibited | PDE3B | -4.3 | -1.5 | -2.5 |
| 222824_at | nudix (nucleoside diphosphate linked moiety X)-type motif 5 | NUDT5 | -2.6 | -2.3 | -2.5 |
| 222931_s_at | threonine synthase-like 1 (S. cerevisiae) | THNSL1 | -3.8 | -1.7 | -2.5 |
| 223211_at | 2-hydroxyacyl-CoA lyase 1 | HACL1 | -3.6 | -1.8 | -2.5 |
| 225354_s_at | SH3 domain binding glutamic acid-rich protein like 2 | SH3BGRL2 | -2.6 | -2.4 | -2.5 |
| 225734_at | F-box protein 22 | FBXO22 | -3.0 | -2.2 | -2.5 |
| 225853_at | glucosamine-phosphate N-acetyltransferase 1 | GNPNAT1 | -4.5 | -1.4 | -2.5 |
| 226096_at | fibronectin type III domain containing 5 | FNDC5 | -2.7 | -2.4 | -2.5 |
| 226216_at | insulin receptor | INSR | -3.3 | -1.9 | -2.5 |
| 226370_at | kelch-like 15 (Drosophila) | KLHL15 | -2.8 | -2.3 | -2.5 |
| 227176_at | solute carrier family 2 (facilitated glucose transporter), member 13 | SLC2A13 | -2.0 | -3.1 | -2.5 |
| 228381_at | Hypothetical protein LOC100287628 | LOC100287628 | -2.7 | -2.4 | -2.5 |
| 229245_at | pleckstrin homology domain containing, family A member 6 | PLEKHA6 | -2.3 | -2.7 | -2.5 |
| 229623_at | Transmembrane protein 150C | TMEM150C | -2.5 | -2.6 | -2.5 |
| 229865_at | fibronectin type III domain containing 3B | FNDC3B | -2.7 | -2.4 | -2.5 |
| 230175_s_at | Discoidin, CUB and LCCL domain containing 2 | DCBLD2 | -2.4 | -2.6 | -2.5 |
| 231152_at | INO80 complex subunit D | INO80D | -2.4 | -2.6 | -2.5 |
| 233198_at | golgin A2 family, member B | GOLGA2B | -2.6 | -2.4 | -2.5 |
| 234980_at | transmembrane protein 56 | TMEM56 | -2.2 | -2.9 | -2.5 |
| 235065_at | family with sequence similarity 59, member A | FAM59A | -2.7 | -2.3 | -2.5 |
| 236831_at | coiled-coil domain containing 50 | CCDC50 | -2.7 | -2.3 | -2.5 |
| 53720_at | chromosome 19 open reading frame 66 | C19orf66 | -3.2 | -2.0 | -2.5 |
| 1555538_s_at | family with sequence similarity 9, member B | FAM9B | -2.6 | -2.3 | -2.4 |
| 200664_s_at | DnaJ (Hsp40) homolog, subfamily B, member 1 | DNAJB1 | -2.7 | -2.1 | -2.4 |
| 202071_at | syndecan 4 | SDC4 | -3.0 | -1.9 | -2.4 |
| 202235_at | solute carrier family 16, member 1 (monocarboxylic acid transporter 1) | SLC16A1 | -3.2 | -1.8 | -2.4 |
| 202449_s_at | retinoid X receptor, alpha | RXRA | -2.3 | -2.6 | -2.4 |
| 203407_at | periplakin | PPL | -2.2 | -2.6 | -2.4 |
| 204067_at | sulfite oxidase | SUOX | -2.5 | -2.3 | -2.4 |
| 204646_at | dihydropyrimidine dehydrogenase | DPYD | -2.3 | -2.6 | -2.4 |
| 204807_at | transmembrane protein 5 | TMEM5 | -2.6 | -2.3 | -2.4 |
| 205190_at | plastin 1 | PLS1 | -2.7 | -2.1 | -2.4 |
| 206449_s_at | mannan-binding lectin serine peptidase 1 (C4/C2 activating component of Ra-reactive factor) | MASP1 | -2.7 | -2.1 | -2.4 |
| 206878_at | D-amino-acid oxidase | DAO | -4.1 | -1.5 | -2.4 |
| 209081_s_at | collagen, type XVIII, alpha 1 | COL18A1 | -2.7 | -2.1 | -2.4 |
| 209340_at | UDP-N-acteylglucosamine pyrophosphorylase 1 | UAP1 | -2.8 | -2.0 | -2.4 |
| 211155_s_at | thrombopoietin | THPO | -3.2 | -1.8 | -2.4 |
| 212274_at | lipin 1 | LPIN1 | -2.1 | -2.8 | -2.4 |
| 212432_at | GrpE-like 1, mitochondrial (E. coli) | GRPEL1 | -3.8 | -1.5 | -2.4 |
| 212867_at | nuclear receptor coactivator 2 | NCOA2 | -2.2 | -2.7 | -2.4 |
| 213375_s_at | NEDD4 binding protein 2-like 1 | N4BP2L1 | -2.1 | -2.6 | -2.4 |
| 214955_at | transmembrane protease, serine 6 | TMPRSS6 | -3.3 | -1.8 | -2.4 |
| 215427_s_at | zinc finger, CCHC domain containing 14 | ZCCHC14 | -2.7 | -2.1 | -2.4 |
| 218487_at | aminolevulinate dehydratase | ALAD | -2.4 | -2.4 | -2.4 |
| 220235_s_at | chromosome 1 open reading frame 103 | C1orf103 | -2.4 | -2.4 | -2.4 |
| 222025_s_at | 5-oxoprolinase (ATP-hydrolysing) | OPLAH | -3.1 | -1.9 | -2.4 |
| 222631_at | phosphatidylinositol 4-kinase type 2 beta | PI4K2B | -2.9 | -2.0 | -2.4 |
| 223412_at | kelch repeat and BTB (POZ) domain containing 7 | KBTBD7 | -1.9 | -2.9 | -2.4 |
| 224895_at | Yes-associated protein 1 | YAP1 | -2.6 | -2.2 | -2.4 |
| 225070_at | nuclear undecaprenyl pyrophosphate synthase 1 homolog (S. cerevisiae) | NUS1 | -2.5 | -2.4 | -2.4 |
| 225078_at | epithelial membrane protein 2 | EMP2 | -2.0 | -2.8 | -2.4 |
| 225599_s_at | chromosome 8 open reading frame 83 | C8orf83 | -2.0 | -2.8 | -2.4 |
| 225840_at | thyrotrophic embryonic factor | TEF | -2.2 | -2.7 | -2.4 |
| 226305_at | Ly6/neurotoxin 1 | LYNX1 | -2.4 | -2.5 | -2.4 |
| 226459_at | phosphoinositide-3-kinase adaptor protein 1 | PIK3AP1 | -2.2 | -2.5 | -2.4 |
| 226498_at | fms-related tyrosine kinase 1 (vascular endothelial growth factor/vascular permeability factor receptor) | FLT1 | -2.7 | -2.2 | -2.4 |
| 227029_at | family with sequence similarity 177, member A1 | FAM177A1 | -3.0 | -2.0 | -2.4 |
| 227224_at | Ral GEF with PH domain and SH3 binding motif 2 | RALGPS2 | -2.6 | -2.2 | -2.4 |
| 228724_at | tubulin tyrosine ligase-like family, member 7 | TTLL7 | -2.1 | -2.9 | -2.4 |
| 229676_at | Mitochondrial poly(A) polymerase | MTPAP | -2.6 | -2.2 | -2.4 |
| 231018_at | paralemmin 3 | PALM3 | -2.9 | -2.0 | -2.4 |
| 233599_at | hCG2003663 | LOC728061 | -2.9 | -2.0 | -2.4 |
| 233609_at | Protein tyrosine phosphatase, receptor type, K | PTPRK | -2.1 | -2.7 | -2.4 |
| 234725_s_at | sema domain, immunoglobulin domain (Ig), transmembrane domain (TM) and short cytoplasmic domain, (semaphorin) 4B | SEMA4B | -2.1 | -2.6 | -2.4 |
| 235722_at | synaptojanin 2 binding protein | SYNJ2BP | -2.4 | -2.4 | -2.4 |
| 236261_at | oxysterol binding protein-like 6 | OSBPL6 | -2.3 | -2.5 | -2.4 |
| 237363_at | chromosome 9 open reading frame 68 | C9orf68 | -2.4 | -2.5 | -2.4 |
| 239513_at | chromosome 22 open reading frame 45 | C22orf45 | -3.0 | -1.8 | -2.4 |
| 243372_at | heat shock 60kDa protein 1 (chaperonin) | HSPD1 | -4.0 | -1.5 | -2.4 |
| 243761_at | cyclin-dependent kinase 14 | CDK14 | -2.3 | -2.5 | -2.4 |
| 1556021_at | --- | --- | -2.4 | -2.3 | -2.3 |
| 1556123_a_at | --- | --- | -2.3 | -2.3 | -2.3 |
| 1556316_s_at | hypothetical LOC284889 | LOC284889 | -2.6 | -2.0 | -2.3 |
| 1556588_at | chromosome 15 open reading frame 37 | C15orf37 | -2.5 | -2.2 | -2.3 |
| 1558815_at | sorbin and SH3 domain containing 2 | SORBS2 | -2.2 | -2.4 | -2.3 |
| 201886_at | DDB1 and CUL4 associated factor 11 | DCAF11 | -2.7 | -2.0 | -2.3 |
| 201940_at | carboxypeptidase D | CPD | -2.5 | -2.2 | -2.3 |
| 202562_s_at | chromosome 14 open reading frame 1 | C14orf1 | -2.5 | -2.1 | -2.3 |
| 202662_s_at | inositol 1,4,5-triphosphate receptor, type 2 | ITPR2 | -2.8 | -1.8 | -2.3 |
| 203116_s_at | ferrochelatase | FECH | -2.9 | -1.8 | -2.3 |
| 203150_at | Rab9 effector protein with kelch motifs | RABEPK | -3.1 | -1.7 | -2.3 |
| 204143_s_at | enolase superfamily member 1 | ENOSF1 | -2.4 | -2.3 | -2.3 |
| 205160_at | peroxisomal biogenesis factor 11 alpha | PEX11A | -3.0 | -1.7 | -2.3 |
| 205380_at | PDZ domain containing 1 | PDZK1 | -3.6 | -1.5 | -2.3 |
| 205627_at | cytidine deaminase | CDA | -2.8 | -1.8 | -2.3 |
| 206170_at | adrenergic, beta-2-, receptor, surface | ADRB2 | -1.8 | -2.9 | -2.3 |
| 206263_at | flavin containing monooxygenase 4 | FMO4 | -2.2 | -2.4 | -2.3 |
| 208249_s_at | TDP-glucose 4,6-dehydratase | TGDS | -2.4 | -2.2 | -2.3 |
| 209605_at | thiosulfate sulfurtransferase (rhodanese) | TST | -3.8 | -1.4 | -2.3 |
| 209694_at | 6-pyruvoyltetrahydropterin synthase | PTS | -3.3 | -1.7 | -2.3 |
| 209799_at | protein kinase, AMP-activated, alpha 1 catalytic subunit | PRKAA1 | -2.8 | -2.0 | -2.3 |
| 210909_x_at | lipoprotein, Lp(a)-like 2, pseudogene | LPAL2 | -2.4 | -2.2 | -2.3 |
| 211456_x_at | metallothionein 1 pseudogene 2 | MT1P2 | -3.8 | -1.4 | -2.3 |
| 212560_at | sortilin-related receptor, L(DLR class) A repeats-containing | SORL1 | -2.4 | -2.3 | -2.3 |
| 212599_at | autism susceptibility candidate 2 | AUTS2 | -2.5 | -2.1 | -2.3 |
| 212859_x_at | metallothionein 1E | MT1E | -4.0 | -1.4 | -2.3 |
| 214787_at | DENN/MADD domain containing 4A | DENND4A | -2.5 | -2.0 | -2.3 |
| 216450_x_at | heat shock protein 90kDa beta (Grp94), member 1 | HSP90B1 | -2.2 | -2.4 | -2.3 |
| 217921_at | mannosidase, alpha, class 1A, member 2 | MAN1A2 | -2.4 | -2.2 | -2.3 |
| 220199_s_at | axin interactor, dorsalization associated | AIDA | -2.4 | -2.3 | -2.3 |
| 221215_s_at | receptor-interacting serine-threonine kinase 4 | RIPK4 | -2.8 | -1.9 | -2.3 |
| 222011_s_at | t-complex 1 | TCP1 | -2.7 | -1.9 | -2.3 |
| 222016_s_at | zinc finger protein 323 | ZNF323 | -2.1 | -2.5 | -2.3 |
| 223047_at | CKLF-like MARVEL transmembrane domain containing 6 | CMTM6 | -2.3 | -2.4 | -2.3 |
| 223712_at | pterin-4 alpha-carbinolamine dehydratase/dimerization cofactor of hepatocyte nuclear factor 1 alpha (TCF1) 2 | PCBD2 | -2.5 | -2.1 | -2.3 |
| 223894_s_at | AKT interacting protein | AKTIP | -2.9 | -1.8 | -2.3 |
| 223961_s_at | cytokine inducible SH2-containing protein | CISH | -2.6 | -2.0 | -2.3 |
| 224151_s_at | adenylate kinase 3 | AK3 | -3.0 | -1.7 | -2.3 |
| 224744_at | inositol monophosphatase domain containing 1 | IMPAD1 | -2.3 | -2.3 | -2.3 |
| 225619_at | SLAIN motif family, member 1 | SLAIN1 | -2.1 | -2.4 | -2.3 |
| 227059_at | glypican 6 | GPC6 | -2.0 | -2.6 | -2.3 |
| 227481_at | CNKSR family member 3 | CNKSR3 | -2.4 | -2.2 | -2.3 |
| 227728_at | protein phosphatase, Mg2+/Mn2+ dependent, 1A | PPM1A | -2.1 | -2.5 | -2.3 |
| 228087_at | coiled-coil domain containing 126 | CCDC126 | -2.0 | -2.5 | -2.3 |
| 228168_at | ATP synthase, H+ transporting, mitochondrial Fo complex, subunit C3 (subunit 9) | ATP5G3 | -2.5 | -2.1 | -2.3 |
| 232098_at | dystonin | DST | -1.9 | -2.7 | -2.3 |
| 236244_at | Heterogeneous nuclear ribonucleoprotein U (scaffold attachment factor A) | HNRNPU | -2.2 | -2.3 | -2.3 |
| 236263_at | sonic hedgehog | SHH | -2.5 | -2.2 | -2.3 |
| 237439_at | ubiquitin specific peptidase 43 | USP43 | -2.5 | -2.1 | -2.3 |
| 239492_at | SEC14-like 4 (S. cerevisiae) | SEC14L4 | -3.0 | -1.8 | -2.3 |
| 239654_at | Chromodomain helicase DNA binding protein 9 | CHD9 | -1.9 | -2.8 | -2.3 |
| 1557953_at | zinc finger with KRAB and SCAN domains 1 | ZKSCAN1 | -2.1 | -2.4 | -2.2 |
| 1558523_at | family with sequence similarity 184, member A | FAM184A | -2.4 | -1.9 | -2.2 |
| 200856_x_at | nuclear receptor corepressor 1 | NCOR1 | -2.2 | -2.2 | -2.2 |
| 201825_s_at | saccharopine dehydrogenase (putative) | SCCPDH | -3.7 | -1.4 | -2.2 |
| 201830_s_at | neuroepithelial cell transforming 1 | NET1 | -2.2 | -2.3 | -2.2 |
| 202447_at | 2,4-dienoyl CoA reductase 1, mitochondrial | DECR1 | -2.9 | -1.7 | -2.2 |
| 202527_s_at | SMAD family member 4 | SMAD4 | -2.0 | -2.3 | -2.2 |
| 203199_s_at | 5-methyltetrahydrofolate-homocysteine methyltransferase reductase | MTRR | -2.2 | -2.1 | -2.2 |
| 203874_s_at | SWI/SNF related, matrix associated, actin dependent regulator of chromatin, subfamily a, member 1 | SMARCA1 | -2.9 | -1.6 | -2.2 |
| 204967_at | shroom family member 2 | SHROOM2 | -2.0 | -2.3 | -2.2 |
| 205189_s_at | Fanconi anemia, complementation group C | FANCC | -2.5 | -2.0 | -2.2 |
| 205657_at | 3-hydroxyanthranilate 3,4-dioxygenase | HAAO | -2.8 | -1.7 | -2.2 |
| 205976_at | FAST kinase domains 2 | FASTKD2 | -2.4 | -2.0 | -2.2 |
| 208991_at | signal transducer and activator of transcription 3 (acute-phase response factor) | STAT3 | -2.6 | -1.9 | -2.2 |
| 209389_x_at | diazepam binding inhibitor (GABA receptor modulator, acyl-CoA binding protein) | DBI | -2.7 | -1.9 | -2.2 |
| 209531_at | glutathione transferase zeta 1 | GSTZ1 | -3.4 | -1.4 | -2.2 |
| 210692_s_at | solute carrier family 43, member 3 | SLC43A3 | -2.6 | -1.8 | -2.2 |
| 212952_at | hypothetical LOC100507328 | LOC100507328 | -2.2 | -2.1 | -2.2 |
| 213140_s_at | synovial sarcoma translocation gene on chromosome 18-like 1 | SS18L1 | -2.9 | -1.7 | -2.2 |
| 218212_s_at | molybdenum cofactor synthesis 2 | MOCS2 | -2.7 | -1.7 | -2.2 |
| 219244_s_at | mitochondrial ribosomal protein L46 | MRPL46 | -2.8 | -1.7 | -2.2 |
| 219355_at | chromosome X open reading frame 57 | CXorf57 | -2.3 | -2.1 | -2.2 |
| 219655_at | chromosome 7 open reading frame 10 | C7orf10 | -2.9 | -1.7 | -2.2 |
| 222354_at | F11 receptor | F11R | -2.7 | -1.9 | -2.2 |
| 222549_at | claudin 1 | CLDN1 | -2.4 | -2.0 | -2.2 |
| 223168_at | ras homolog gene family, member U | RHOU | -1.9 | -2.6 | -2.2 |
| 223290_at | pyridoxal (pyridoxine, vitamin B6) phosphatase  SH3-domain binding protein 1 | PDXP  SH3BP1 | -2.7 | -1.7 | -2.2 |
| 223402_at | dual specificity phosphatase 23 | DUSP23 | -2.5 | -1.8 | -2.2 |
| 223729_at | cat eye syndrome chromosome region, candidate 2 | CECR2 | -2.4 | -2.1 | -2.2 |
| 224779_s_at | family with sequence similarity 96, member A | FAM96A | -2.3 | -2.1 | -2.2 |
| 224881_at | vitamin K epoxide reductase complex, subunit 1-like 1 | VKORC1L1 | -1.9 | -2.5 | -2.2 |
| 225534_at | chromosome 8 open reading frame 40 | C8orf40 | -2.2 | -2.1 | -2.2 |
| 226756_at | --- | --- | -1.9 | -2.7 | -2.2 |
| 226939_at | cytoplasmic polyadenylation element binding protein 2 | CPEB2 | -2.1 | -2.2 | -2.2 |
| 227407_at | transmembrane anterior posterior transformation 1 | TAPT1 | -2.3 | -2.1 | -2.2 |
| 228196_s_at | La ribonucleoprotein domain family, member 4B | LARP4B | -2.0 | -2.3 | -2.2 |
| 229581_at | extracellular leucine-rich repeat and fibronectin type III domain containing 1 | ELFN1 | -2.2 | -2.3 | -2.2 |
| 231517_at | zyg-11 homolog A (C. elegans) | ZYG11A | -2.7 | -1.8 | -2.2 |
| 232244_at | KIAA1161 | KIAA1161 | -3.3 | -1.5 | -2.2 |
| 234092_s_at | transmembrane 6 superfamily member 2 | TM6SF2 | -3.2 | -1.6 | -2.2 |
| 237327_at | anterior pharynx defective 1 homolog A (C. elegans) | APH1A | -2.6 | -1.9 | -2.2 |
| 242214_at | ribosomal protein S27a | RPS27A | -2.4 | -2.0 | -2.2 |
| 243278_at | forkhead box P2 | FOXP2 | -2.4 | -1.9 | -2.2 |
| 244758_at | SCAN domain containing 3 | SCAND3 | -2.4 | -1.9 | -2.2 |
| 1555334_s_at | solute carrier family 30 (zinc transporter), member 5 | SLC30A5 | -2.3 | -2.0 | -2.1 |
| 1558703_at | solute carrier family 46 (folate transporter), member 1 | SLC46A1 | -1.9 | -2.4 | -2.1 |
| 1559096_x_at | F-box protein 9 | FBXO9 | -2.3 | -2.0 | -2.1 |
| 1560935_s_at | hypothetical protein LOC284669 | LOC284669 | -2.3 | -2.0 | -2.1 |
| 1563512_at | Nitric oxide synthase 1 (neuronal) adaptor protein | NOS1AP | -2.2 | -2.1 | -2.1 |
| 201170_s_at | basic helix-loop-helix family, member e40 | BHLHE40 | -2.5 | -1.8 | -2.1 |
| 201490_s_at | peptidylprolyl isomerase F | PPIF | -2.8 | -1.7 | -2.1 |
| 201831_s_at | USO1 vesicle docking protein homolog (yeast) | USO1 | -2.6 | -1.6 | -2.1 |
| 202675_at | succinate dehydrogenase complex, subunit B, iron sulfur (Ip) | SDHB | -2.6 | -1.7 | -2.1 |
| 203465_at | mitochondrial ribosomal protein L19 | MRPL19 | -2.9 | -1.4 | -2.1 |
| 203504_s_at | ATP-binding cassette, sub-family A (ABC1), member 1 | ABCA1 | -1.9 | -2.4 | -2.1 |
| 203733_at | Dexi homolog (mouse) | DEXI | -2.3 | -2.0 | -2.1 |
| 205384_at | FXYD domain containing ion transport regulator 1 | FXYD1 | -1.9 | -2.3 | -2.1 |
| 206453_s_at | NDRG family member 2 | NDRG2 | -1.7 | -2.6 | -2.1 |
| 207071_s_at | aconitase 1, soluble | ACO1 | -3.1 | -1.5 | -2.1 |
| 209706_at | NK3 homeobox 1 | NKX3-1 | -2.4 | -1.9 | -2.1 |
| 211026_s_at | monoglyceride lipase | MGLL | -2.3 | -1.9 | -2.1 |
| 212185_x_at | metallothionein 2A | MT2A | -3.4 | -1.3 | -2.1 |
| 212423_at | zinc finger, CCHC domain containing 24 | ZCCHC24 | -1.7 | -2.7 | -2.1 |
| 213549_at | PDZ domain containing 8 | PDZD8 | -2.6 | -1.7 | -2.1 |
| 214764_at | ribosomal RNA processing 15 homolog (S. cerevisiae) | RRP15 | -2.2 | -2.0 | -2.1 |
| 215898_at | tubulin tyrosine ligase-like family, member 5 | TTLL5 | -2.1 | -2.1 | -2.1 |
| 216396_s_at | etoposide induced 2.4 mRNA | EI24 | -2.6 | -1.7 | -2.1 |
| 216595_at | family with sequence similarity 186, member A | FAM186A | -2.4 | -1.8 | -2.1 |
| 216640_s_at | protein disulfide isomerase family A, member 6 | PDIA6 | -2.4 | -1.8 | -2.1 |
| 217803_at | golgi phosphoprotein 3 (coat-protein) | GOLPH3 | -2.3 | -1.9 | -2.1 |
| 218124_at | retinol saturase (all-trans-retinol 13,14-reductase) | RETSAT | -2.9 | -1.5 | -2.1 |
| 218789_s_at | chromosome 11 open reading frame 71 | C11orf71 | -2.5 | -1.9 | -2.1 |
| 218948_at | glutaminyl-tRNA synthase (glutamine-hydrolyzing)-like 1 | QRSL1 | -2.0 | -2.2 | -2.1 |
| 219499_at | Sec61 alpha 2 subunit (S. cerevisiae) | SEC61A2 | -2.4 | -1.8 | -2.1 |
| 220233_at | F-box protein 17  seryl-tRNA synthetase 2, mitochondrial | FBXO17 SARS2 | -2.8 | -1.6 | -2.1 |
| 222209_s_at | transmembrane protein 135 | TMEM135 | -2.3 | -1.9 | -2.1 |
| 222291_at | family with sequence similarity 149, member A | FAM149A | -1.7 | -2.5 | -2.1 |
| 225334_at | chromosome 10 open reading frame 32 | C10orf32 | -2.3 | -2.0 | -2.1 |
| 225337_at | abhydrolase domain containing 2 | ABHD2 | -3.1 | -1.4 | -2.1 |
| 225550_at | consortin, connexin sorting protein | CNST | -2.0 | -2.3 | -2.1 |
| 225863_s_at | chromosome 19 open reading frame 12 | C19orf12 | -2.2 | -2.0 | -2.1 |
| 226360_at | zinc and ring finger 3 | ZNRF3 | -2.7 | -1.6 | -2.1 |
| 228005_at | zinc finger, X-linked, duplicated B | ZXDB | -2.7 | -1.6 | -2.1 |
| 228341_at | nudix (nucleoside diphosphate linked moiety X)-type motif 16 | NUDT16 | -2.0 | -2.2 | -2.1 |
| 228418_at | exocyst complex component 5 | EXOC5 | -2.3 | -1.9 | -2.1 |
| 229128_s_at | Acidic (leucine-rich) nuclear phosphoprotein 32 family, member E | ANP32E | -2.4 | -1.9 | -2.1 |
| 229334_at | RUN and FYVE domain containing 3 | RUFY3 | -2.0 | -2.1 | -2.1 |
| 229394_s_at | glucocorticoid receptor DNA binding factor 1 | GRLF1 | -2.0 | -2.2 | -2.1 |
| 229691_at | zinc finger and BTB domain containing 42 | ZBTB42 | -2.1 | -2.1 | -2.1 |
| 229905_at | RAP1, GTP-GDP dissociation stimulator 1 | RAP1GDS1 | -2.3 | -1.9 | -2.1 |
| 231042_s_at | --- | --- | -1.9 | -2.4 | -2.1 |
| 232810_at | androgen-induced 1 | AIG1 | -2.4 | -1.9 | -2.1 |
| 233520_s_at | cardiomyopathy associated 5 | CMYA5 | -2.2 | -2.0 | -2.1 |
| 235022_at | chromosome 18 open reading frame 19 | C18orf19 | -2.8 | -1.6 | -2.1 |
| 235420_at | hyaluronan and proteoglycan link protein 4 | HAPLN4 | -2.2 | -2.0 | -2.1 |
| 235653_s_at | THAP domain containing 6 | THAP6 | -1.5 | -2.9 | -2.1 |
| 239576_at | --- | --- | -2.3 | -1.9 | -2.1 |
| 241949_at | acyl-CoA thioesterase 6 | ACOT6 | -2.3 | -1.9 | -2.1 |
| 1552477_a_at | interferon regulatory factor 6 | IRF6 | -1.9 | -2.1 | -2.0 |
| 1555679_a_at | reticulon 4 interacting protein 1 | RTN4IP1 | -2.5 | -1.7 | -2.0 |
| 201689_s_at | tumor protein D52 | TPD52 | -2.4 | -1.7 | -2.0 |
| 202762_at | Rho-associated, coiled-coil containing protein kinase 2 | ROCK2 | -2.1 | -1.9 | -2.0 |
| 203991_s_at | lysine (K)-specific demethylase 6A | KDM6A | -1.9 | -2.2 | -2.0 |
| 204161_s_at | ectonucleotide pyrophosphatase/phosphodiesterase 4 (putative) | ENPP4 | -1.9 | -2.2 | -2.0 |
| 209581_at | phospholipase A2, group XVI | PLA2G16 | -2.3 | -1.9 | -2.0 |
| 210131_x_at | succinate dehydrogenase complex, subunit C, integral membrane protein, 15kDa | SDHC | -2.4 | -1.7 | -2.0 |
| 211689_s_at | transmembrane protease, serine 2 | TMPRSS2 | -2.4 | -1.7 | -2.0 |
| 212289_at | ankyrin repeat domain 12 | ANKRD12 | -1.7 | -2.4 | -2.0 |
| 213333_at | malate dehydrogenase 2, NAD (mitochondrial) | MDH2 | -2.6 | -1.6 | -2.0 |
| 213435_at | SATB homeobox 2 | SATB2 | -2.3 | -1.8 | -2.0 |
| 213851_at | transmembrane protein 110 | TMEM110 | -2.2 | -1.9 | -2.0 |
| 214155_s_at | La ribonucleoprotein domain family, member 4 | LARP4 | -2.7 | -1.5 | -2.0 |
| 214545_s_at | proline synthetase co-transcribed homolog (bacterial) | PROSC | -2.2 | -1.9 | -2.0 |
| 219521_at | beta-1,3-glucuronyltransferase 1 (glucuronosyltransferase P) | B3GAT1 | -2.1 | -2.0 | -2.0 |
| 219648_at | melanoregulin | MREG | -1.9 | -2.1 | -2.0 |
| 221960_s_at | RAB2A, member RAS oncogene family | RAB2A | -2.1 | -1.9 | -2.0 |
| 221965_at | M-phase phosphoprotein 9 | MPHOSPH9 | -2.3 | -1.8 | -2.0 |
| 222957_at | sialidase 4 | NEU4 | -2.7 | -1.5 | -2.0 |
| 223218_s_at | nuclear factor of kappa light polypeptide gene enhancer in B-cells inhibitor, zeta | NFKBIZ | -2.2 | -1.9 | -2.0 |
| 224654_at | DEAD (Asp-Glu-Ala-Asp) box polypeptide 21 | DDX21 | -2.6 | -1.6 | -2.0 |
| 225272_at | spermidine/spermine N1-acetyltransferase family member 2 | SAT2 | -2.3 | -1.8 | -2.0 |
| 226464_at | chromosome 3 open reading frame 58 | C3orf58 | -1.9 | -2.1 | -2.0 |
| 226807_at | zinc finger protein 1 homolog (mouse) | ZFP1 | -1.9 | -2.2 | -2.0 |
| 227256_at | ubiquitin specific peptidase 31 | USP31 | -2.3 | -1.8 | -2.0 |
| 227554_at | hypothetical LOC100505881 | LOC100505881 | -1.8 | -2.2 | -2.0 |
| 228770_at | G protein-coupled receptor 146 | GPR146 | -2.6 | -1.5 | -2.0 |
| 230238_at | ankyrin repeat domain 43 | ANKRD43 | -2.5 | -1.6 | -2.0 |
| 230243_at | RNA (guanine-9-) methyltransferase domain containing 2 | RG9MTD2 | -2.5 | -1.7 | -2.0 |
| 230774_at | prostaglandin reductase 2 | PTGR2 | -2.5 | -1.6 | -2.0 |
| 235611_at | splicing regulatory glutamine/lysine-rich protein 1 | SREK1 | -1.7 | -2.4 | -2.0 |
| 240757_at | Cytoplasmic linker associated protein 1 | CLASP1 | -2.0 | -2.0 | -2.0 |

* Transcript name based on probe target sequence. MHN denotes massive hepatic necrosis. SHN denotes submassive hepatic necrosis. LD denotes liver donors. ALF denotes acute liver failure.
